# A Simple Biomimetic Receptor Selectively Recognizing the GlcNAc_2_ Disaccharide in Water

**DOI:** 10.1002/anie.202100560

**Published:** 2021-04-06

**Authors:** Oscar Francesconi, Francesco Milanesi, Cristina Nativi, Stefano Roelens

**Affiliations:** ^1^ Department of Chemistry “Ugo Schiff” and INSTM University of Florence, Polo Scientifico e Tecnologico 50019 Sesto Fiorentino Firenze Italy; ^2^ Magnetic Resonance Center CERM Via L. Sacconi 6 50019 Sesto Fiorentino Firenze Italy

**Keywords:** carbohydrates, chitobiose, hydrogen bonds, molecular recognition, receptors

## Abstract

GlcNAc_2_ is the core disaccharide fragment present in *N*‐glycans exposed on the surface of enveloped viruses of high health concern, such as coronaviruses. Because *N*‐glycans are directly involved in the docking of viruses to host cells, recognition of GlcNAc_2_ by a biomimetic receptor may be a convenient alternative to the use of lectins to interfere with viral entry and infection. Herein, we describe a simple biomimetic receptor recognizing the methyl‐β‐glycoside of GlcNAc_2_ in water with an unprecedented affinity of 160 μM, exceeding that of more structurally complex receptors reported in the literature. The tweezers‐shaped acyclic structure exhibits marked selectivity among structurally related disaccharides, and complete discrimination between mono‐ and disaccharides. Molecular modelling calculations supported by NOE data provided a three‐dimensional description of the binding mode, shedding light on the origin of the affinities and selectivities exhibited by the receptor.

## Graphical abstract

Think simple. In the development of biomimetic receptors for carbohydrates, an approach that focuses on simple acyclic architectures can be successfully exploited for disaccharide recognition, provided that the correct binding geometry can be achieved. Here we present a simple tweezers‐shaped receptor that selectively recognizes in water GlcNAc_2_, the core fragment of *N*‐glycans exposed on the surface of enveloped viruses, such as coronaviruses.

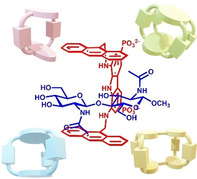

Enveloped viruses are a broad class of highly glycosylated viroids of high health concern, including coronaviruses (SARS‐CoV‐2, SARS‐CoV and MERS), retroviruses (HIV and hepatitis B), orthomyxoviruses (influenza A‐C), flaviviruses (dengue, hepatitis C, yellow fever, Zika) and filoviruses (Ebola and Marburg fever), among others.^[1]^ Viral adhesion to host cells is often mediated by specific carbohydrate‐protein interactions, which occur through the glycans exposed on the surface of the viral envelope.^[2]^ Biomimetic receptors for carbohydrates targeting these saccharides may inhibit virus‐cell interaction, thereby preventing viral entry and infection.[^3^, ^4^] In this context, among biologically relevant oligosaccharides, *N*,*N*′‐diacetylchitobiose (GlcNAc_2_) holds a pivotal role, because is part of the highly conserved GlcNAc_2_Man_3_ core fragment of *N*‐glycans present on the surface of enveloped viruses, constituting the disaccharidic unit N‐linked to membrane proteins through an asparagine residue, which often get exposed by mannoside deletions due to virus mutations.[^5^, ^6^] Unsurprisingly, GlcNAc‐binding lectins, such as NICTABA from *Nicotiana tabacum* and *Urtica dioica* Agglutinin (UDA), which target GlcNAc_2_ at the stem of N‐glycosilation sites, possess a broad‐spectrum activity against several families of enveloped viruses,^[7]^ Thus, effective molecular recognition of GlcNAc_2_ in water by a simple and easily accessible biomimetic receptor can potentially represent a convenient alternative to natural lectins, because of advantages in terms of availability, molecular weight, purity, stability and immunogenicity.

Selective recognition of neutral glycans by biomimetic receptors in physiological medium represents a major challenge of current research,[^4^, ^8^] because water is a strong competitor for recognition of polar molecules such as carbohydrates.^[9]^ Nevertheless, in the last few years, significant steps forward have been made in the design of receptors for mono‐ and oligosaccharides, mainly developing appropriately sized macrocyclic architectures.[^8^, ^10^] The main drawback of the latter strategy is that macrocyclic architectures must be precisely tailored on specific saccharidic targets and often require lengthy multistep syntheses with relatively low overall yields, due to the critical macrocyclization step.

We have recently developed a new generation biomimetic receptor for monosaccharides in water, by assembling into a macrocyclic architecture a tridentate hydrogen binding motif (1,8‐diaminocarbazole endowed with two hydrosolubilizing phosphonate groups) with anthracene moieties providing extended CH‐π interactions with the saccharidic backbone.[^11^, ^12^] Interestingly, the corresponding adaptive tweezers‐shaped liposoluble receptor proved to effectively recognize biologically relevant xanthines in organic solvents.^[13]^ Following the idea that an acyclic adaptive structure may accommodate disaccharides more effectively than its macrocyclic counterpart, we tested receptor **1**, the hydrosoluble version of the parent receptor **2**,^[13]^ vs. a set of mono‐ and disaccharides in water, in the belief that effective recognition of disaccharides may be achieved by a simple, easily available structure.^[14]^ We report here that this is true indeed, and that **1** is not only a simple and easily accessible receptor for disaccharides, but also the most effective biomimetic receptor for GlcNAc_2_ in the literature up to date.

Compound **1** was easily obtained by hydrolysis of the previously reported ester **2** (Scheme 1).^[13]^ **1** is freely soluble in water under both, mild alkaline (pH 11) and physiological (pH 7.4) conditions, whereas precipitates at acidic pH, due to high degree of protonation of phosphonate groups. Receptor **1** shows sharp ^1^H NMR signals at low concentration values (5×10^4^ M), broadening at higher values at pH 7.4, but not at pH 11, suggesting concentration‐dependent self‐association, supported by chemical shift changes.

**Scheme 1:**
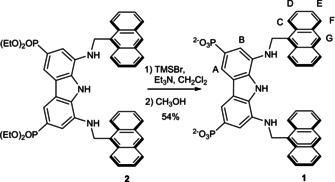
Synthesis of receptor **1** and proton labels.

The binding properties of receptor **1** were qualitatively screened by ^1^H NMR spectroscopy toward a set of monosaccharides, including glucose, rhamnose, fucose, xylose, sialic acid, α and β methyl glucosides, galactosides, mannosides, and *N*‐acetylglucosamine (Figure 1 a), together with a set of disaccharides, including sucrose (Suc), trehalose (Tre), cellobiose (CeB), maltose (Mal) and lactose (Lac) (Figure 1 b) by monitoring the shifts of the proton signals of the sugar upon addition of an equimolar amount of **1**. Surprisingly, little (Δ*δ*≤0.03 ppm) or no variations were observed for all the investigated monosaccharides and for Suc and Tre, whereas a marked upfield shift was observed for CeB, Mal and Lac, which were larger for the β than for the α anomer (Figures S3–S5), reasonably due to the shielding effect of the anthracene moieties in the binding cavity. A concomitant broadening of signals, larger for the CeB, indicated slow chemical exchange, most likely due to strong binding, suggesting a preference for all‐equatorial disaccharides.

**Figure 1:**
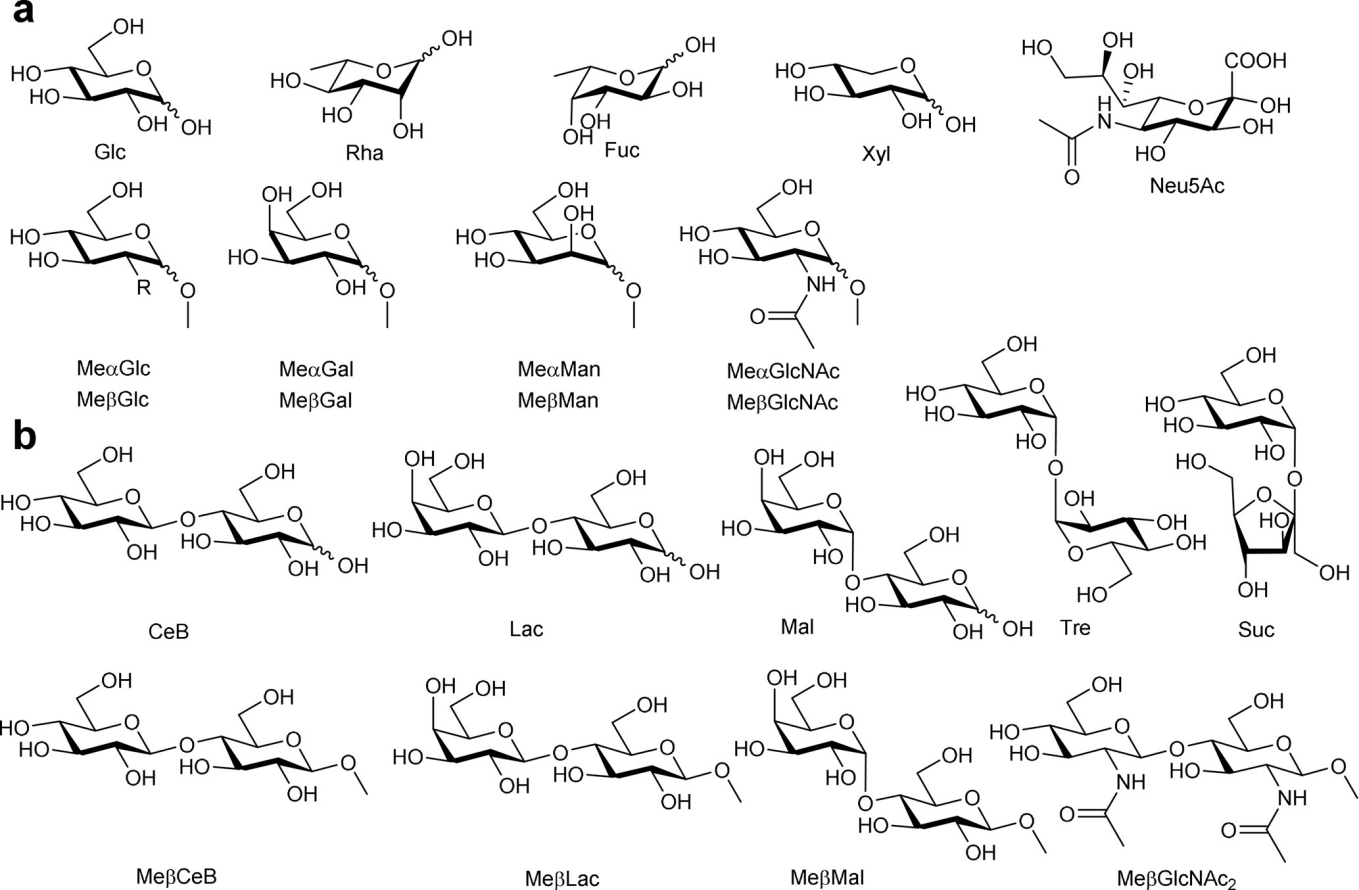
Structure of the investigated a) monosaccharides and b) disaccharides and their abbreviations.

A quantitative investigation was then carried out by NMR spectroscopy, extending the study to the all‐equatorial GlcNAc_2_. Because in *N*‐glycans the GlcNAc_2_ glycoside unit is present as the β anomer, methyl‐β‐glycosides of GlcNAc_2_ (MeβGlcNAc_2_), cellobiose (MeβCeB), maltose (MeβMal) and lactose (MeβLac) (Figure 1 a) were employed, to avoid interconversion equilibria between anomers. Dilution experiments of receptor **1** were preliminary carried out at pH 7.4, fitting a self‐association model featuring two clusters, in which the dimer was the predominant species at low concentrations. The fit gave a dimerization constant of log*β*_dim_=2.65±0.07, most likely due to π stacking of the aromatic moieties, which was set invariant in the nonlinear regression analysis of the glycoside binding experiments. The cumulative association constants, reported in Table 1, were measured by ^1^H NMR titrations in D_2_O (pD 7.4) at 298 K, simultaneously fitting the complexation induced shifts of all the available signals to the appropriate association model by non‐ linear regression analysis. Because multiple binding constants were measured, affinities were assessed through the intrinsic median binding concentration parameter *BC*_50_^0^,^[15]^ calculated from the measured binding constants and reported in Table 1. Amazingly, results show that receptor **1** binds MeβGlcNAc_2_ with an affinity of 160 μM which is unprecedented in the literature for a synthetic receptor. Indeed, to the best of our knowledge, the highest affinity reported to date for MeβGlcNAc_2_ by a biomimetic receptor is that observed by Davis and co‐workers with a bicyclic polyamidic receptor, showing a 3‐fold lower affinity than **1** (*BC*_50_^0^=455 μM, *K*_a_=2200 M^−1^).^[16]^ The affinity of **1** for MeβGlcNAc_2_ exceeds that of some lectin‐like proteins, such as hevein from *Hevea brasiliensis*, which shows for GlcNAc_2_ an affinity one order of magnitude smaller (*BC*_50_^0^=1.61 mM, *K*_a_=620±50).^[17]^ Moreover, receptor **1** exhibits a marked selectivity, showing an affinity for MeβCeB nearly one order of magnitude smaller, and a 200‐fold drop of affinity for MeβMal and MeβLac. ^1^H NMR titrations with MeβCeB were also duplicated at pD 11 (Table S1, Supporting Information) and fitted to the association model obtained at pD 7.4. The closely comparable affinities confirmed that the degree of protonation of the phosphonate groups does not affect the binding ability of receptor **1**. Most remarkably, recognition of monosaccharides appears completely depleted, as appreciated from the preliminary screening and from the titration of MeβGlcNAc, in which no significant variation of chemical shifts was observed (Figure S13). Somewhat counterintuitively, a flexible acyclic structure exhibits excellent affinities and selectivities, overriding those of more structurally complicated macrocyclic architectures.^[8]^

**Table 1:** Cumulative formation constants (log *β*_n_)^[a]^ and intrinsic median binding concentration (*BC*_50_^0^, mM)^[b]^ for receptor to glycoside (R:G) complexes of **1** with methyl glycosides, measured at 298 K from NMR data in D_2_O at pD 7.4 and from ITC data in H_2_O at pH 7.4.^[c]^

		NMR		ITC	
Glycoside	R:G	log *β*	*BC*_50_^0^	log *β*	*BC*_50_^0^
MeβCeB	1:1	2.53±0.07	0.94±0.10	2.58±0.03	3.52±2.35
	2:1	6.33±0.06		5.28±0.29	
MeβMal	1:1	2.27±0.01	31.0±4.4	2.24±0.01	34.4±4.9
MeβLac	1:1	2.27±0.02	30.8±4.7	2.31±0.02	26.1±4.1
MeβGlcNAc_2_	1:1	3.55±0.04	0.16±0.01	3.49±0.07	0.12±0.03
	2:1	7.35±0.09		7.71±0.22

[a] Formation constants were obtained by nonlinear least‐square regression analysis of NMR and ITC data. [b] Calculated from the log *β* values using the “BC50 Calculator” program.^[15]^ [c] Receptor **1** dimerization constant (log *β*_dim_=2.65±0.07) was set invariant in the nonlinear regression analysis of NMR and ITC data.

The binding affinities obtained by NMR were further confirmed by ITC in H_2_O at physiological pH. Data from two to three independent titrations run at different reactant concentrations were combined and simultaneously fitted to remove ambiguities in the definition of the binding model. The dimerization constant obtained by NMR dilution experiments at pD 7.4 was set invariant in the nonlinear regression analysis of ITC data. Cumulative binding constants, together with affinity values, were reported in Table 1 for a direct comparison with NMR results. The good agreement between the two independent techniques confirmed the observed affinities. Unfortunately, because of the strong self‐association of receptor **1**, ITC measurements did not provide reliable thermodynamic parameters.

To shed light on the origin of the affinities and selectivities exhibited by receptor **1**, a three‐dimensional description of the binding mode was attempted for the 1:1 complex of **1** with MeβGlcNAc_2_, using molecular modelling calculations supported by NOE data from NMR spectroscopy. NOESY spectra carried out on an equimolar mixture of **1** and MeβGlcNAc_2_ showed unambiguous intermolecular NOE contacts between the H‐2 and H′−6′ protons, belonging to the two monosaccharide units, and the H‐C protons of the anthracenes (Figure S20). In addition, NOE contacts were found between the methyl protons of the *N*‐acetyl group of the methylglycosidic unit with the H‐C protons of the anthracene and the H‐A and H‐B protons of the carbazole. In contrast, no NOE contacts could be found for the second *N*‐acetyl group.

A conformational search carried out on the 1:1 complex returned a single family of minimum energy conformers within 10.0 kJ mol^−1^ from the global minimum. The minimum energy structure depicted in Figure 2 shows the disaccharide entirely located inside the binding cleft, within the two anthracene faces, with the H‐2 and H′−6′ protons pointing toward the H‐C protons of the anthracenes, in agreement with the proximities inferred by NOE contacts (Figure 2 a). In addition, the *N*‐acetyl group of the methylglycosidic unit faces the diaminocarbazole moiety, pointing the methyl protons toward the H‐A and H‐B protons, and to one of the H‐C protons of the anthracene. From the above model, all O⋅⋅⋅H interatomic distances shorter than the sum of the van der Waals radii and compliant with hydrogen bonding criteria were calculated and four hydrogen bonding interactions were found between **1** and MeβGlcNAc_2_ (Figure 2 b): the carbazole NH and one of the aminic NH behave as donors toward OH‐3 of the methylglycosidic unit, whereas the other aminic NH of the receptor acts as both, donor to the OH‐3 and acceptor from the amidic NH of the *N*‐acetyl group. In addition to CH‐π interactions with the anthracene units,^[18]^ the *N*‐acetyl group contributes to stabilize the complex through CH‐π interactions with the carbazole unit. Most likely, the substantial participation to binding of the N‐acetyl group may account for the observed selectivity over other disaccharides.

**Figure 2:**
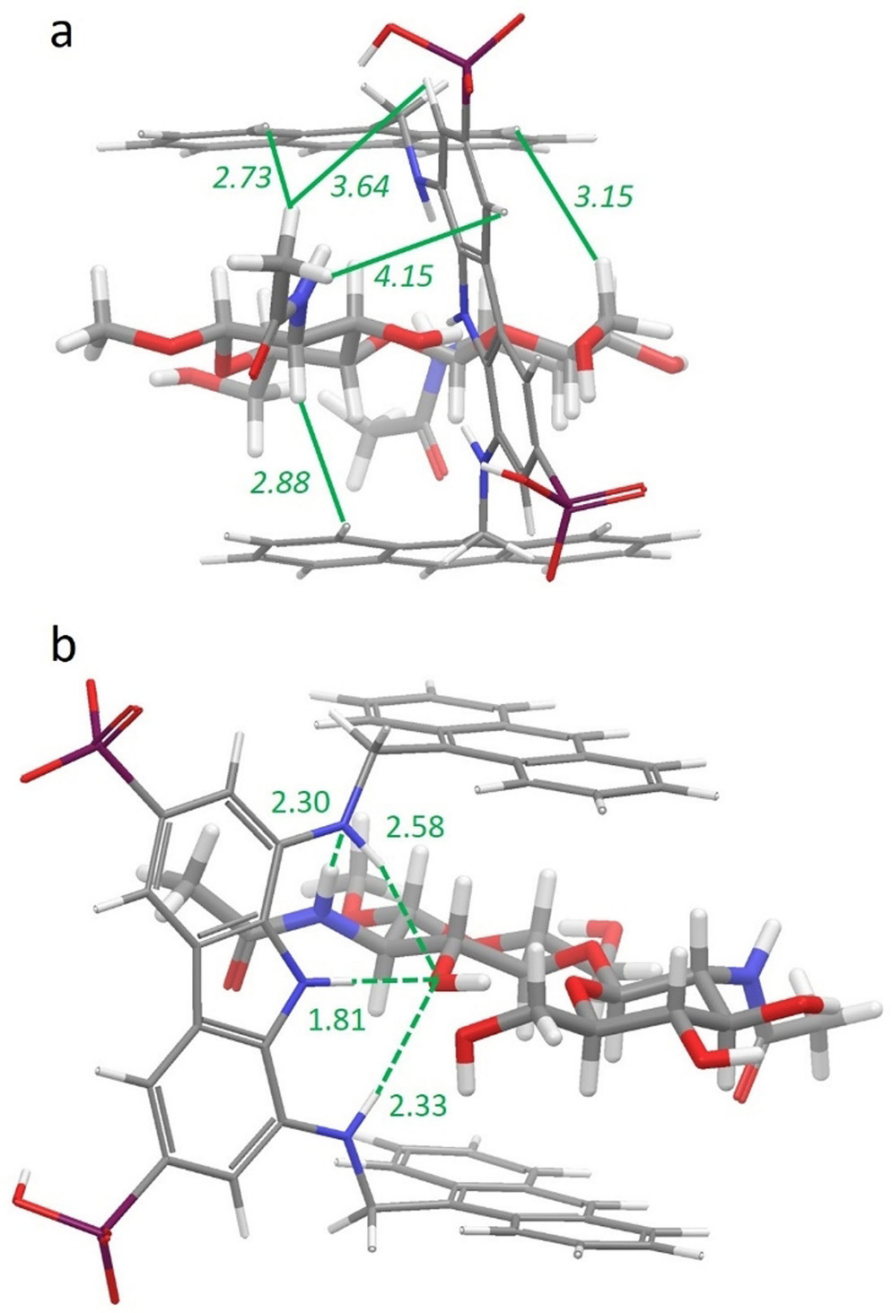
Global minimum structure of the **1** MeβGlcNAc_2_ complex in two different projections. a) The strongest intermolecular NOEs found between **1** and MeβGlcNAc_2_ are indicated as solid lines with corresponding distances [Å] calculated from the lowest energy conformer. b) Intermolecular hydrogen‐bonding interactions found in the calculated structure are indicated as dashed lines with corresponding oxygen/hydrogen and nitrogen/hydrogen distances [Å].

In this context, it is worth mentioning that, interestingly, the binding mode of **1** with MeβGlcNAc_2_ is reminiscent of that between hevein and the corresponding trisaccharide chitotriose, as reported by the group of J. Jiménez‐Barbero (Figure 3).[^17^, ^19^] A close similarity can be appreciated between the binding mode of chitotriose to Tyr30 of hevein and of chitobiose to the aminocarbazole of **1**. Indeed, both receptors engage hydrogen‐bonding with the saccharidic OH‐3 and with the acetamidic NH, while in both cases the methyl group stabilizes the complex through CH‐π interactions with the aromatic ring.

**Figure 3:**
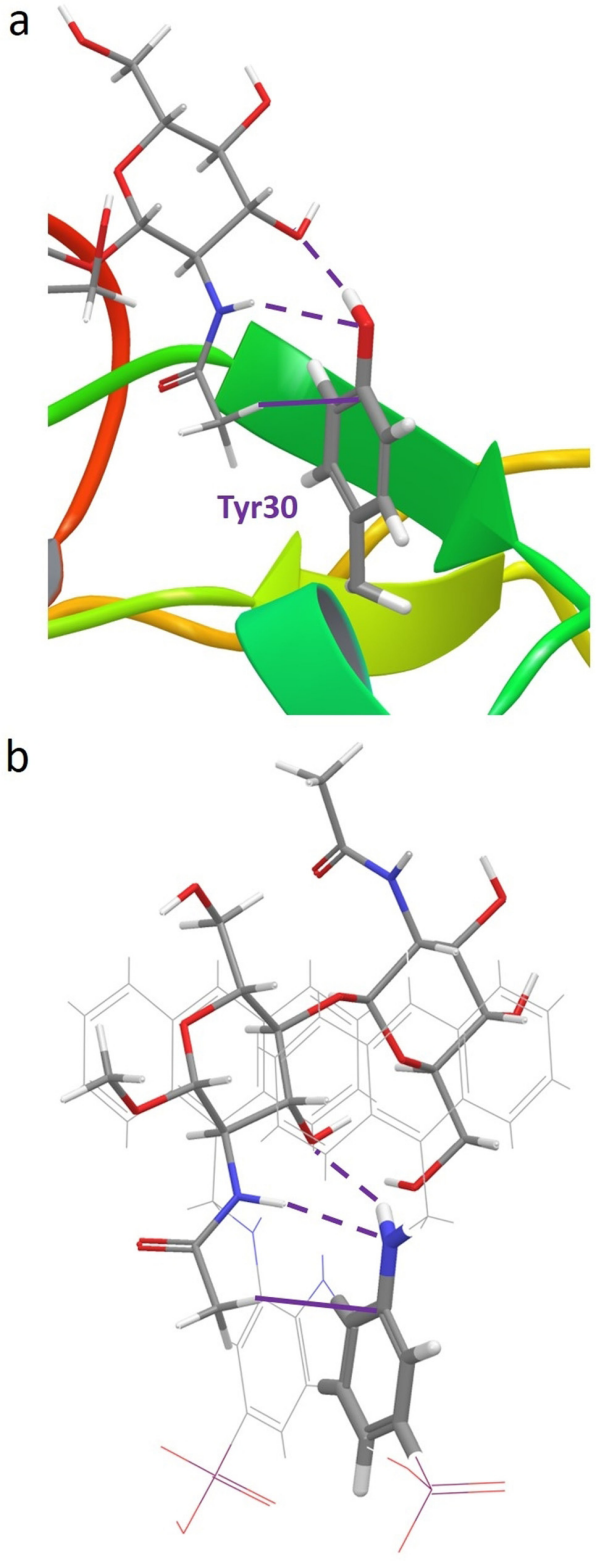
Comparison of the global minimum structures of a) hevein⋅chitotriose complex and b) **1** ^.^ MeβGlcNAc_2_ complex. Intermolecular hydrogen‐bonding and CH–π interactions involving Tyr30 and the arylamine moiety of **1** are indicated as dashed and solid lines, respectively. Protein backbone was drawn as a ribbon for clarity.

Altogether, the present work shows that molecular recognition of a disaccharide can effectively and selectively be achieved with a well‐designed acyclic host featuring an adaptive architecture. The tweezers‐shaped receptor **1** fully discriminates disaccharides from monosaccharides, selectively recognizes all‐equatorial from non all‐equatorial disaccharides, and shows an unprecedented affinity for GlcNAc_2_, the core glycosidic fragment of viral *N*‐glycans. The hydrogen‐bonding and CH‐π interactions established by receptor **1** with the *N*‐acetyl group most likely account for the selectivity observed for MeβGlcNAc_2_ over other all‐equatorial disaccharides. Because of simple structure, easy synthetic availability, and accessible structure modifications, receptor **1** stands as a promising tool for saccharide recognition.

## Conflict of interest

The authors declare no conflict of interest.

## Supporting information

As a service to our authors and readers, this journal provides supporting information supplied by the authors. Such materials are peer reviewed and may be re‐organized for online delivery, but are not copy‐edited or typeset. Technical support issues arising from supporting information (other than missing files) should be addressed to the authors.

Supplementary
