# Supplementary material for: A Simple Biomimetic Receptor Selectively Recognizing the GlcNAc2 Disaccharide in Water
Source: Angew Chem Int Ed Engl. 2021 Apr 6;60(20):11168–72. doi: 10.1002/anie.202100560 (PMC8252438; doi:10.1002/anie.202100560)
Supplement: Supplementary file 1 — Supplementary [file ANIE-60-11168-s001.pdf]

Angewandte Chemie
Eine Zeitschrift der Gesellschaft Deutscher Chemiker

Supporting Information

A Simple Biomimetic Receptor Selectively Recognizing the $GlcNAc_2$ Disaccharide in Water

Oscar Francesconi, Francesco Milanesi, Cristina Nativi, and Stefano Roelens**

anie_202100560_sm_miscellaneous_information.pdf

SUPPORTING INFORMATION

**Synthesis and characterization of chemical materials**	**p. S2**
**General**	**p. S2**
**Materials**	**p. S2**
**NMR Spectra**	**p. S4**
**Binding studies**	**p. S6**
**NMR preliminary screening**	**p. S6**
**NMR titrations and data analysis**	**p. S8**
**Calorimetric titrations and data analysis**	**p. S42**
**Structural studies**	**p. S63**
**NMR methods**	**p. S63**
**Molecular modeling methods**	**p. S66**
**References**	**p. S67**

Synthesis and characterization of chemical materials.

General. ESI-MS analyses were performed in negative ion mode and were recorded on an LCQ-Fleet Ion Trap equipped with a standard Ionspray interface. HRMS were performed on a Triple-TOF with a resolution of 35000 (FWHM). Chemical shifts are reported in part per million (δ) relative to 4,4-Dimethyl-4-silapentane-1-sulfonic acid (DSS) for D_2O, using the residual solvent line as secondary internal reference (4.79 ppm for spectra run in D_2O). ^{13}C NMR spectra were obtained at 125 MHz in D_2O. Chemical shifts are reported in δ relative to DSS for D_2O.

Materials. Reagents were purchased from commercial suppliers and used without purification. Compound **2** was prepared according to known methods[13]

Receptor 1.

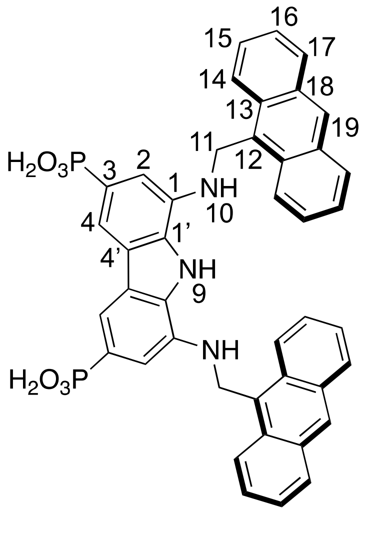

To a solution of **2** (809 mg, 0.950 mmol) in 6.4 mL of dry CH_2Cl_2, Et_3N (784 mg, 7.62 mmol) was added under a nitrogen atmosphere. The solution was cooled to 0 °C and TMSBr (1.17 g, 7.62 mmol) was slowly added. The solution was stirred at room temperature for 22 h, then diluted with 25 mL of CH_2Cl_2 and cooled to 0 °C. After addition of 50 mL of MeOH, the solution was stirred at room temperature for 30 minutes and the solvent evaporated to give 1.84 g of crude **2** as a pale brown solid. The solid was dissolved in 50 mL of NaOH 0.3 M under an ice bath and the aqueous phase washed with AcOEt (4 x 30 mL). EtOH (120 mL) was then added to the aqueous phase and the resulting suspension was centrifugated at 3000 rpm for 20 minutes. The liquids were removed, the solid was dissolved in water (40 mL), then, NaOH 0.5 M was added under an ice bath until pH = 12 was reached. Then, HCl 0.5 M was added (160 mL) and the resulting suspension was centrifugated at 3000 rpm for 20 minutes. The liquids were removed and the solid was suspended in

160 mL of HCl 0.1 M: The suspension was centrifugated at 3000 rpm for 20 minutes, the liquids were removed and the solid was suspended in MeOH (2 x 40 mL) and the resulting suspension centrifugated at 3000 rpm for 20 minutes. The liquids were removed, and the yellow solid was dried in vacuo to obtain pure **2** (380 mg, 54%). Mp: 145.2 (dec) ºC; ^{1}H NMR (500 MHz, D_2O, DSS as internal reference, ∂ = 0.0): ∂ 8.02 (d, *J* = 11.9 Hz, 2H, C*H*-4); 7.86 (d, *J* = 9.0 Hz, 2H, C*H*-14); 7.53 (d, *J* = 11.6 Hz, 2H, C*H*-2); 7.16-7.13 (m, 6H, C*H*-15, C*H*-19); 7.00 (bs, 8H, C*H*-16, C*H*-17); 4.93 (s, 4H, CH_2-11) ; ^{13}C NMR (125 MHz, D_2O, DSS as internal reference, ∂ = 0.0): ∂ 135.93 (d, , *J* = 143.2 Hz); 135.2 (d, *J* = 10.7 Hz); 133.8; 133.29; 132.35; 132.28, 131.31 (*C*H-17); 129.99 (*C*H-19); 128.95 (*C*H-15); 127.60 (*C*H-16); 126.33 (*C*H-14); 125.93 (d, *J* = 16.3 Hz); 117.81 (*C*H-4); 117.73 (*C*H-2); 45.05 (CH_2-11); ^{31}P NMR (202 MHz, D_2O): ∂ 12.73; ESI-MS m/z (%): 367.67 (100%) $[M-2H]^{2-}$, 736.25 (86%) $[M-1H]^{-}$; HRMS (m/z): $[M-2H]^{2-}$ calcd. for $C_{42}H_{33}N_3O_6P_2$, 367.58496; found, 367.58727.

NMR spectra.

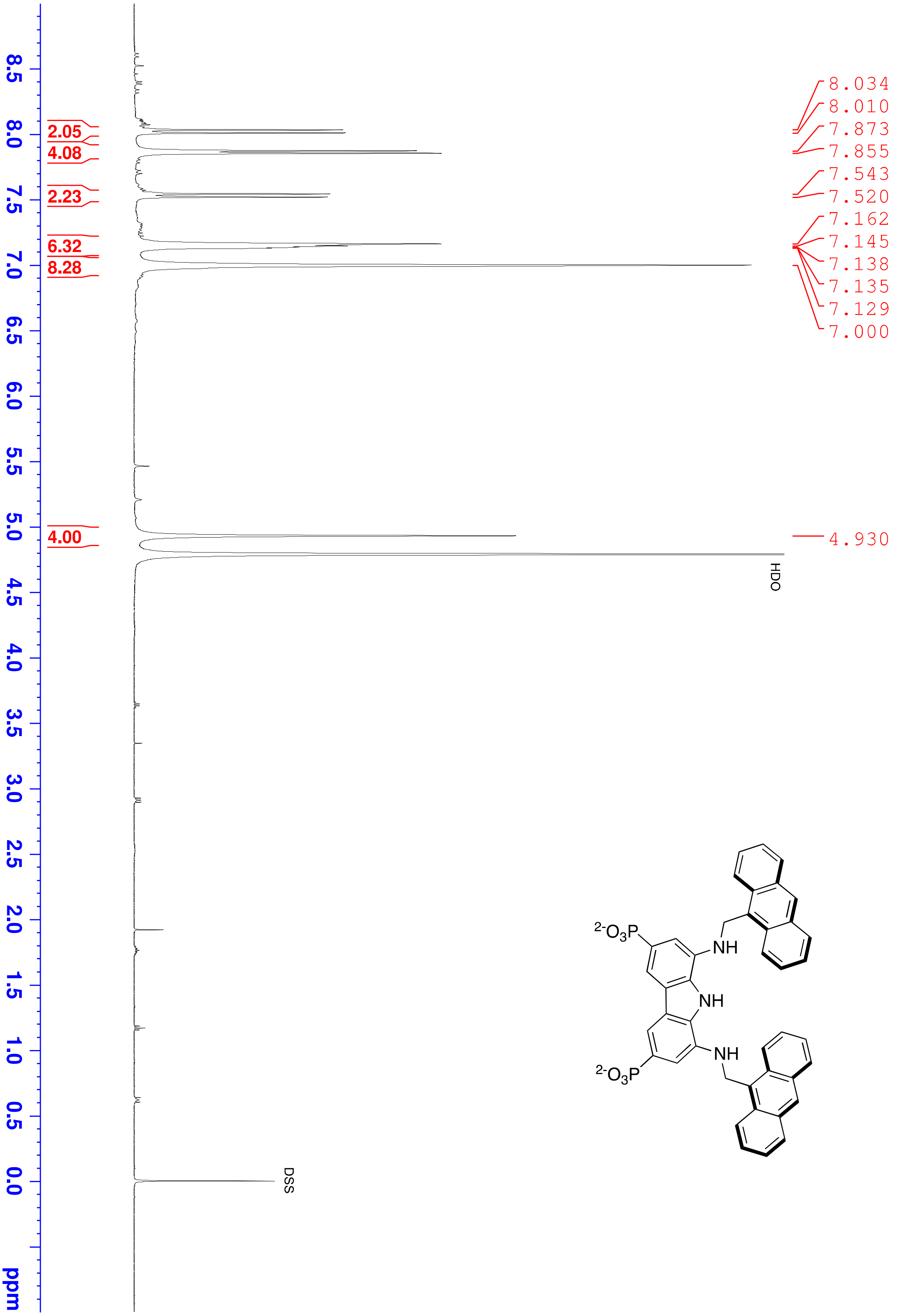

Figure S1. 1H NMR spectrum of receptor **1** (500 MHz, D_2O + DSS).

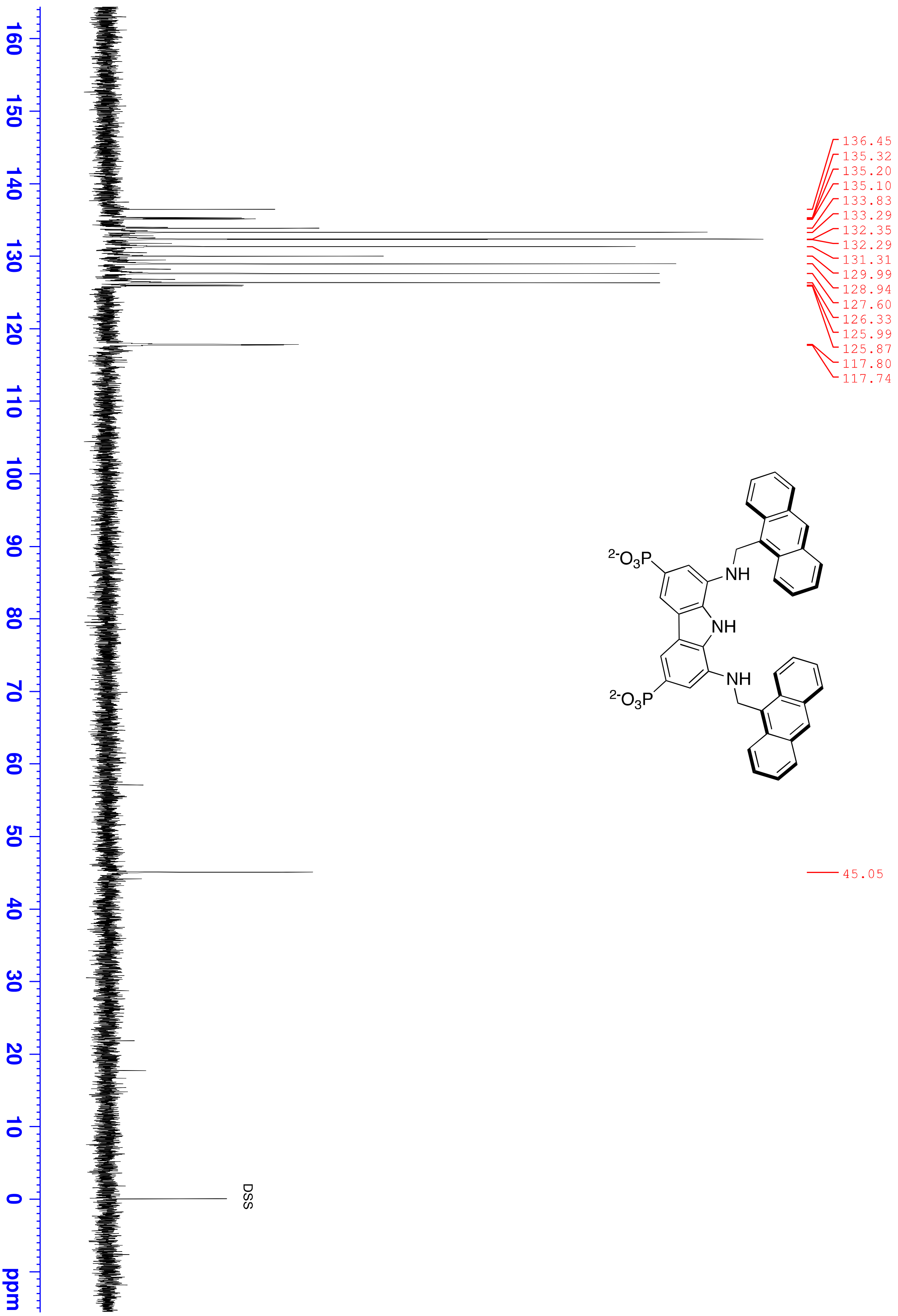

Figure S2. ^{13}C NMR spectrum of receptor **1** (125 MHz, D_2O + DSS).

Binding studies.

NMR preliminary screening. Preliminary screenings (298 K, 500 MHz) were performed in D_2O at pD 11 in presence of DSS as internal reference. Solution of reducing carbohydrates were prepared in D_2O and kept overnight at room temperature before the screening experiments, to ensure equilibration of the anomers. The spectra of the free sugars at 1 mM concentration were compared to the spectra of the equimolar mixture of sugars with receptor **1** (1 mM each) and chemical shift differences were evaluated.

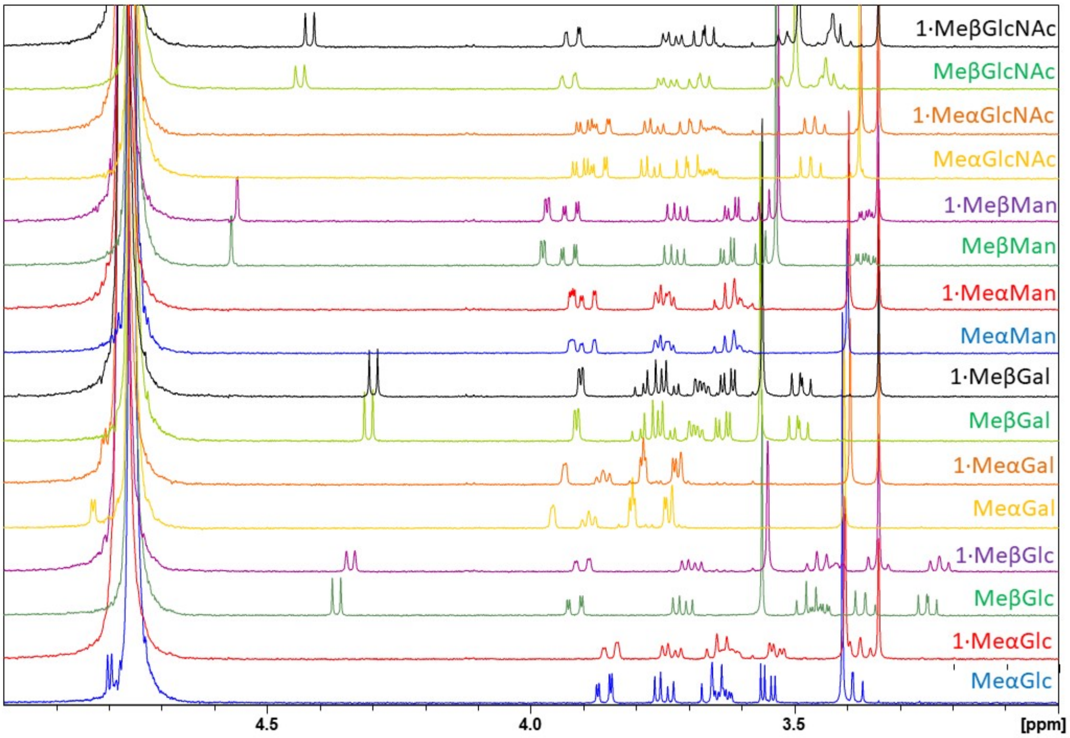

Figure S3. ^{1}H NMR spectra (500 MHz, D_2O) of a 1 mM solution of methyl glycosides and an equimolar mixture of methyl glycosides and **1** (1 mM each) at pD 11.

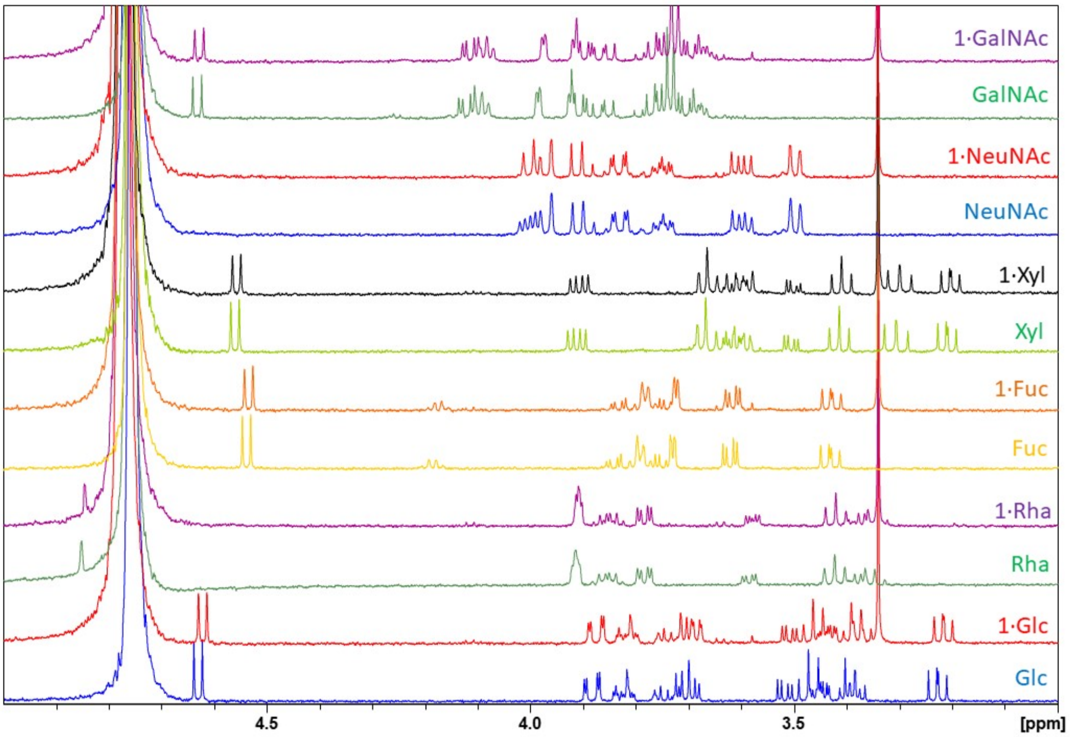

Figure S4. ^{1}H NMR spectra (500 MHz, D_2O) of a 1 mM solution of monosaccharides and an equimolar mixture of monosaccharides and **1** (1 mM each) at pD 11.

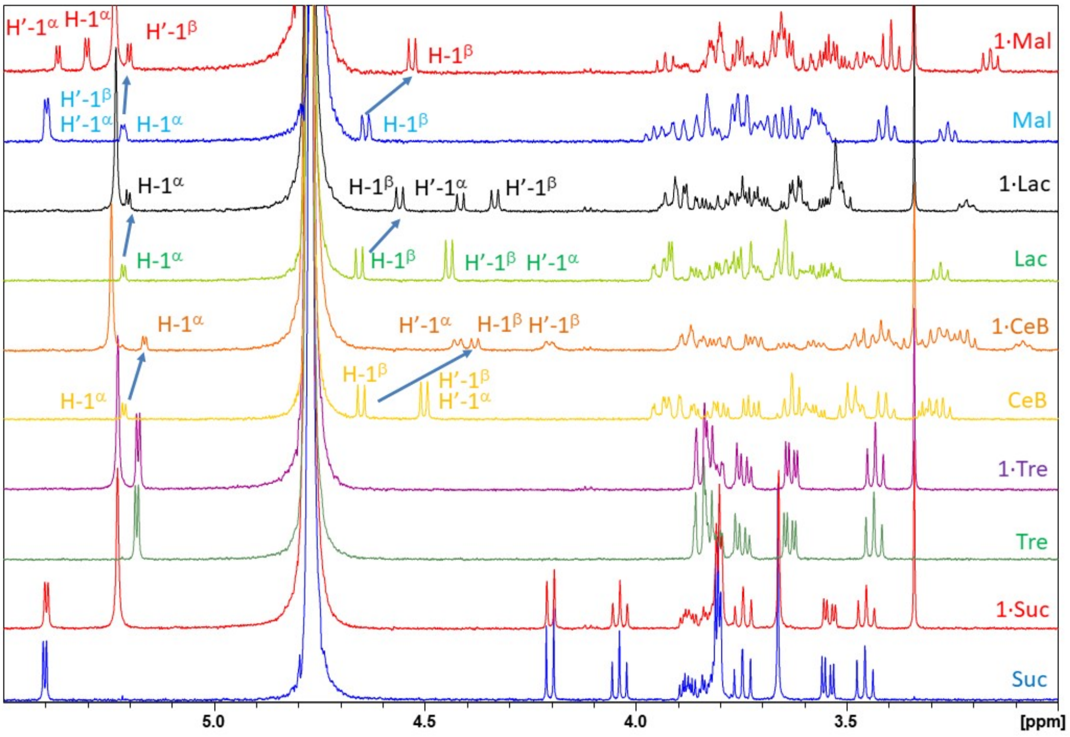

Figure S5. ^{1}H NMR spectra (500 MHz, D_2O) of a 1 mM solution of disaccharides and an equimolar mixture of disaccharides and **1** (1 mM each) at pD 11. Variations of the H-1 proton signal shifts of CeB, Lac, Mal are $\Delta\delta$ = 0.05, 0.01, 0.03 ppm for the α and $\Delta\delta$ = 0.23, 0.10, 0.11 ppm for the β anomer respectively.

NMR titrations and data analysis. Titrations were performed at 298 K, 500 MHz in 5 mm NMR tubes using microsyringes, following a previously described technique.[S1] Concentration of the receptor was measured using an internal standard ($DMSO_2$) preliminarily to each binding measurement and maintained constant during the titrations with glycosides to avoid changes in ionic strength. The stock solutions of **1** were prepared in D_2O adjusting the pD with a diluted NaOH solution in D_2O. A correction factor of +0.4 was applied to the pH values measured by the pH meter to determine the pD values (pD = pH + 0.4). DSS was used as internal reference. The alkaline stock solution of **1** was stored during the titrations under nitrogen atmosphere to avoid acid/base reactions with atmospheric CO_2. Following this strategy, constant values of pD were observed during titrations and dilution experiments. DSS was used as internal reference. Dimerization constants of receptor **1** at pD 7.4 and at pD 11, were set invariant in the non-linear regression analysis of receptor-glycosides binding data measured at pD 7.4 and pD 11, respectively. Mathematical analysis of data and graphic presentation of results was performed using the HypNMR 2006 program.[S2] BC_{50} Calculator, the utility program for computing BC_{50}^{0},[15] is available for free at the corresponding author's e-mail address. Results pages and Plots of experimental and calculated shifts are reported hereafter.

Dilution of receptor 1 (D_2O, pD 7.4, 298 K, 500 MHz).

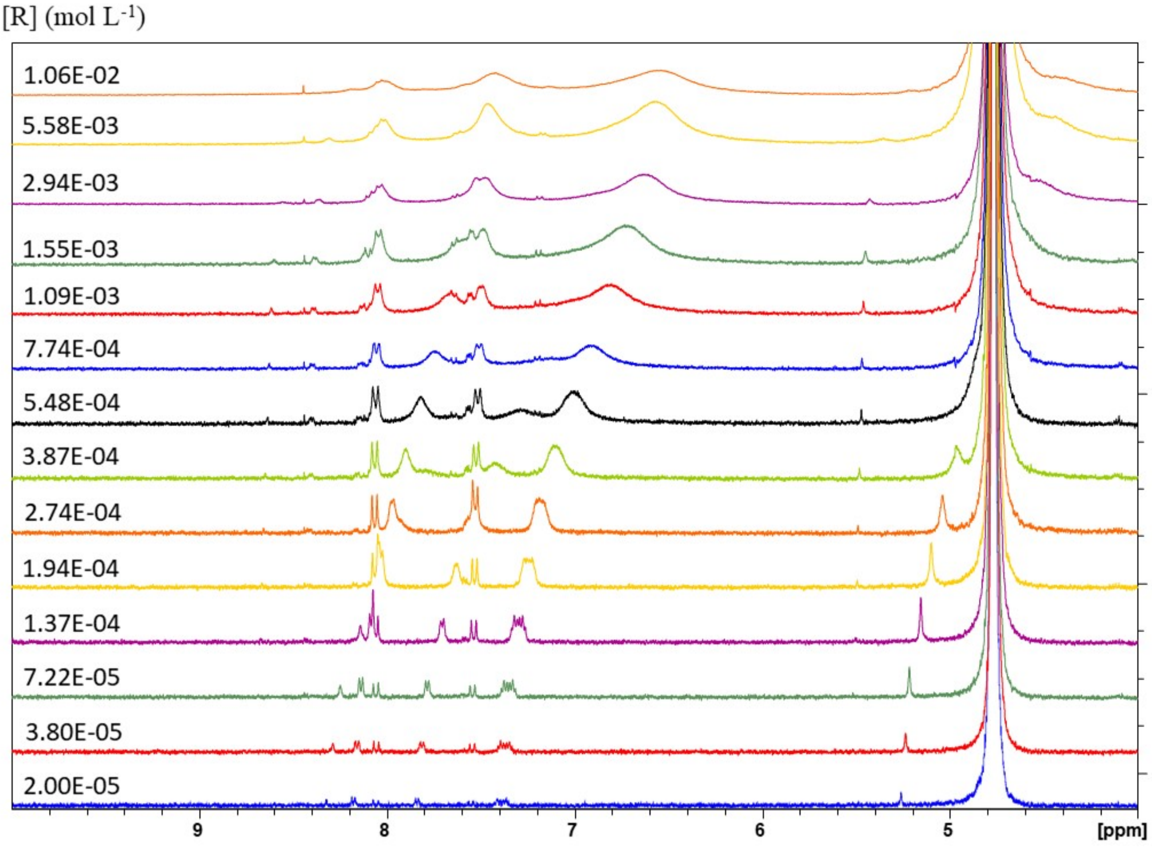

Figure S6. ^{1}H NMR spectroscopic spectra (500 MHz, D_2O, pD 7.4, 298 K) of receptor **1** (R) at different concentrations in dilution expertiment.

Data Table

δ (ppm) *vs*. [R] (mol L^{-1})

[R]	CH-G R	CH-C R	CH-F R	CH-B R	CH-2 R
2.00E-05	8.3231	8.1791	7.8391	7.5525	5.2589
3.80E-05	8.2915	8.1632	7.8166	7.5496	5.2409
7.22E-05	8.2501	8.1404	7.7874	7.5459	5.2171
1.37E-04	8.1445	8.0865	7.7081	7.5396	5.1577
1.94E-04	-	8.0357	7.6338	7.5355	5.1051
2.74E-04	7.9351	7.9737	7.5398	7.5307	5.0411
3.87E-04	7.7970	7.9014	7.4248	7.5259	4.9638
5.48E-04	-	7.8232	7.2940	7.5189	-
7.74E-04	-	7.7493	7.1330	7.5106	-
1.09E-03	-	7.6710	-	7.5008	-
1.55E-03	-	7.5981	-	7.4939	-
2.94E-03	-	-	-	7.4748	4.5290
5.58E-03	-	7.4669	-	7.4655	4.4540
1.06E-02	-	7.4177	-	7.4247	4.4120

Results page

```
 no. of spectra            14
 no. of resonance values   52
 no. of resonant nuclei     5

Chi-squared =   7.38

   sigma =   0.00472549283          RMS weighted residual =   0.00387685411

      stoich          value   relative    log    standard
      coeff                   std devn    beta   deviation

 Beta  2 refined  4.4211E+002  0.1557    2.6455   0.0676  (   R2  )
 Beta  4 refined  2.5653E+009  0.2599    9.4091   0.1129  (   R4  )

 Individual chemical shifts

                   R                       2
 =========================================================
       +       value    error         value    error
CH-G   +      8.3574   0.0047        6.4205   0.3035
CH-C   +      8.2006   0.0039        7.1043   0.1514
CH-F   +      7.8633   0.0045        6.4857   0.2426
CH-B   +      7.5491   0.0032        7.5032   0.0267
CH-2   +      5.2787   0.0039        4.1586   0.1697
       +
                   4
 =================================
       +       value    error
CH-G   +      6.6141   0.1198
CH-C   +      7.3655   0.0109
CH-F   +      6.3413   0.0454
CH-B   +      7.4446   0.0051
CH-2   +      4.3405   0.0119

 Correlation coefficients*1000


      1    2
  1
  2  834

 Parameters are numbered as follows
    1 beta 2
    2 beta 4
```

Titration Plots

Chemical shifts (δ, ppm) *vs*. concentration of R (mol L^{-1})

experimental (symbols) and calculated (lines) values

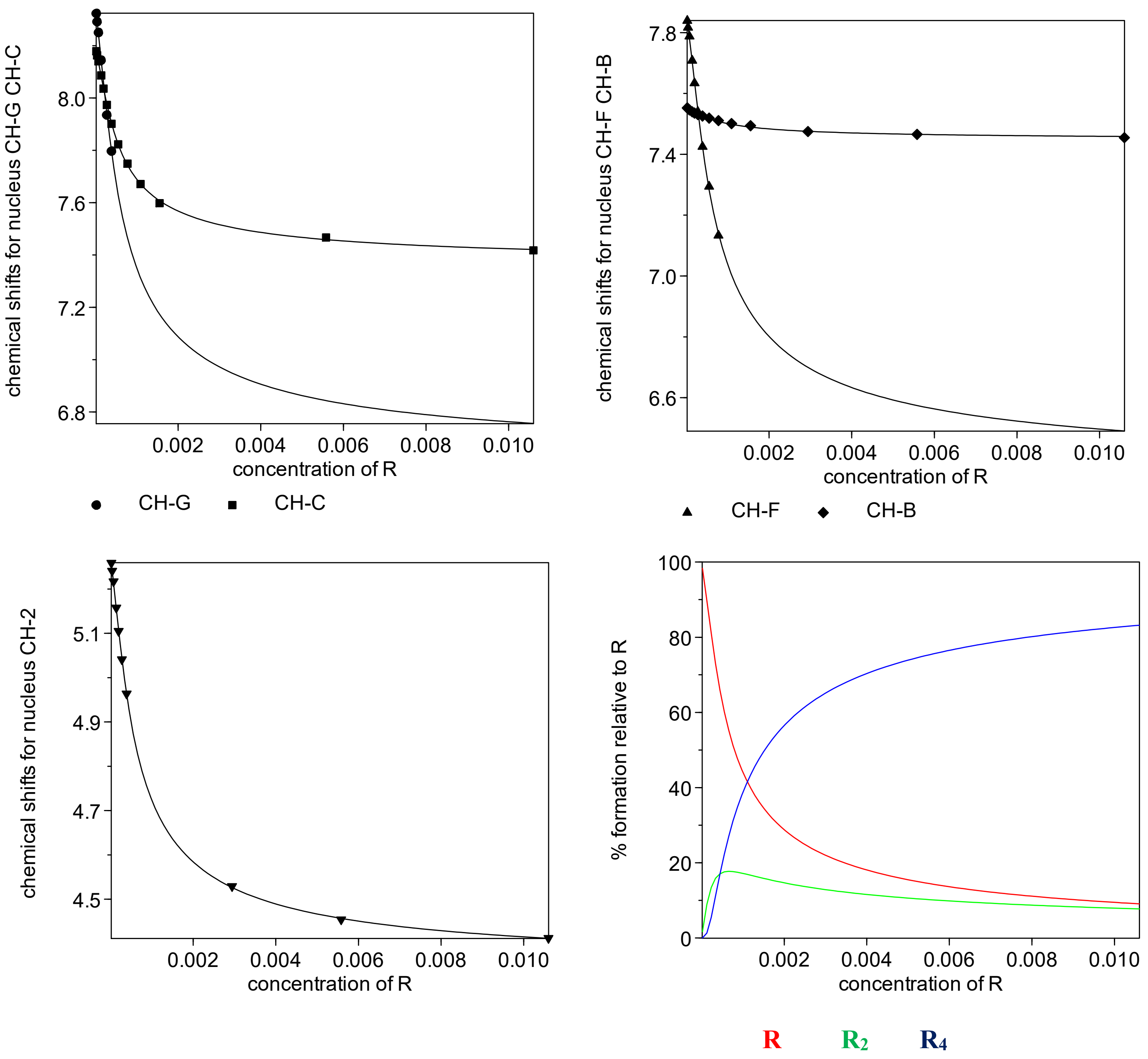

Dilution of receptor 1 (D_2O, pD 11, 298 K, 500 MHz).

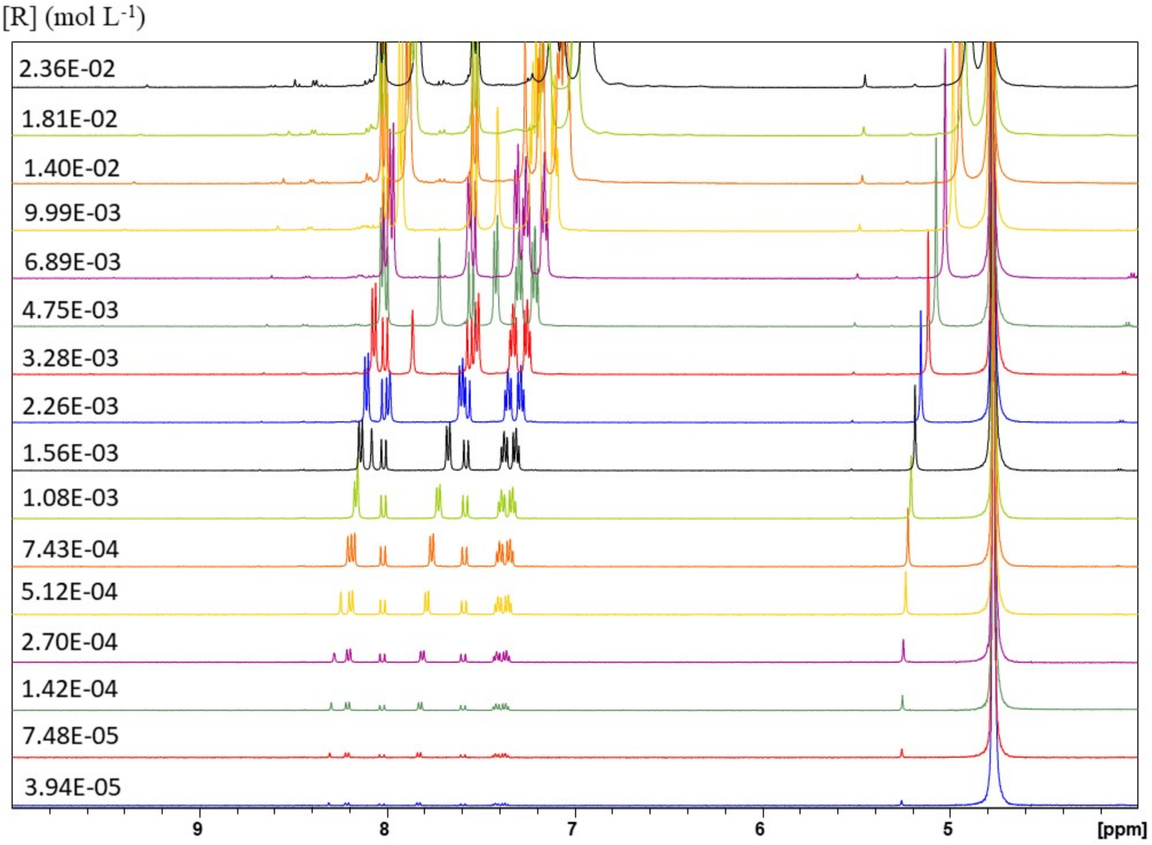

Figure S7. ^{1}H NMR spectroscopic spectra (500 MHz, D_2O, pD 11, 298 K) of receptor **1** (R) at different concentrations in dilution expertiment.

Data Table

R = **1**

δ (ppm) *vs*. [R] (mol L^{-1})

[R]	CH-G R	CH-C R	CH-A R	CH-F R	CH-B R
3.94E-05	8.3117	8.2243	8.0424	7.8428	7.6089
7.48E-05	8.3070	8.2235	8.0419	7.8400	7.6096
1.42E-04	8.2989	8.2207	8.0405	7.8337	7.6088
2.70E-04	8.2829	8.2154	8.0395	7.8226	7.6078
5.12E-04	8.2488	8.2045	8.0380	7.7990	7.6052
7.43E-04	8.2116	8.1925	8.0364	7.7728	7.6021
1.08E-03	8.1598	8.1758	8.0342	7.7381	7.5978
1.56E-03	8.0836	8.1506	8.0313	7.6835	-
2.26E-03	7.9856	8.1188	8.0280	7.6144	7.5837
3.28E-03	7.8659	8.0800	8.0245	7.5308	7.5746
4.75E-03	7.7224	8.0340	-	7.4291	7.5645
6.89E-03	7.5706	7.9863	8.0217	7.3208	7.5561
9.99E-03	7.4104	7.9382	8.0249	-	7.5493
1.40E-02	7.2670	7.8981	8.0305	7.0950	7.5449
1.81E-02	7.1583	7.8725	8.0362	-	7.5428
2.36E-02	7.0649	7.8495	8.0429	6.9289	7.5416

Results page

```
no. of spectra            16
no. of resonance values   92
no. of resonant nuclei     6

  sigma =   0.00105982572          RMS weighted residual =   0.00089083489

     stoich          value   relative    log    standard
     coeff                   std devn    beta   deviation

Beta  2 refined  1.4667E+003  0.1658    3.1663   0.0720  (   R2   )
Beta  4 refined  4.5958E+008  0.2472    8.6624   0.1074  (   R4   )
Beta  8 refined  2.0568E+019  0.4825   19.3132   0.2096  (   R8   )

Individual chemical shifts

                  R                       2
=========================================================
       +       value    error         value    error
CH-G   +      8.3177   0.0011        8.2536   0.0114
CH-C   +      8.2258   0.0010        8.2093   0.0038
CH-A   +      8.0434   0.0009        8.0333   0.0018
CH-F   +      7.8479   0.0010        7.7994   0.0084
CH-B   +      7.6101   0.0009        7.6054   0.0020
CH-2   +      5.2626   0.0009        5.2400   0.0039
       +
                  4                       8
=========================================================
       +       value    error         value    error
CH-G   +      6.3761   0.0719        6.4302   0.0307
CH-C   +      7.5794   0.0219        7.7824   0.0250
CH-A   +      7.9777   0.0046        8.1913   0.0221
CH-F   +      6.5057   0.0526        6.3138   0.0242
CH-B   +      7.4546   0.0057        7.6127   0.0181
CH-2   +      4.6419   0.0208        4.8604   0.0263

Correlation coefficients*1000


     1    2    3
  1
  2  990
  3  890  939

Parameters are numbered as follows
   1 beta 2
   2 beta 4
   3 beta 8
```

Titration Plots

Chemical shifts (δ, ppm) *vs*. concentration of R (mol L^{-1})

experimental (symbols) and calculated (lines) values

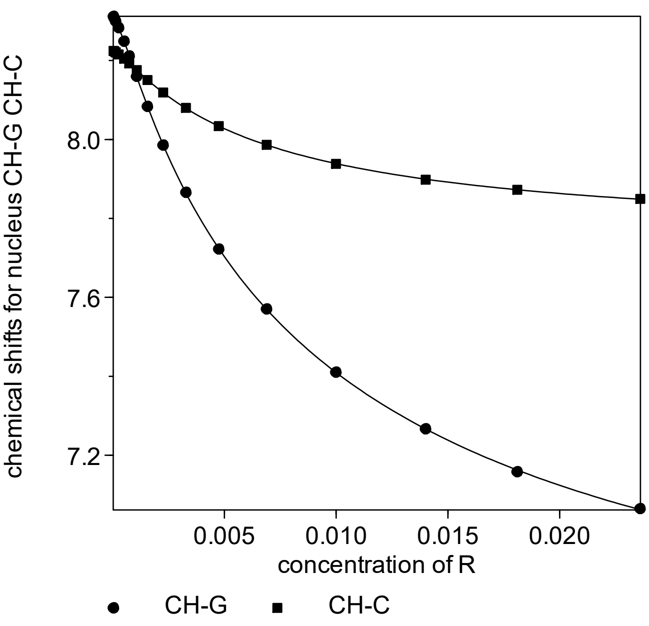

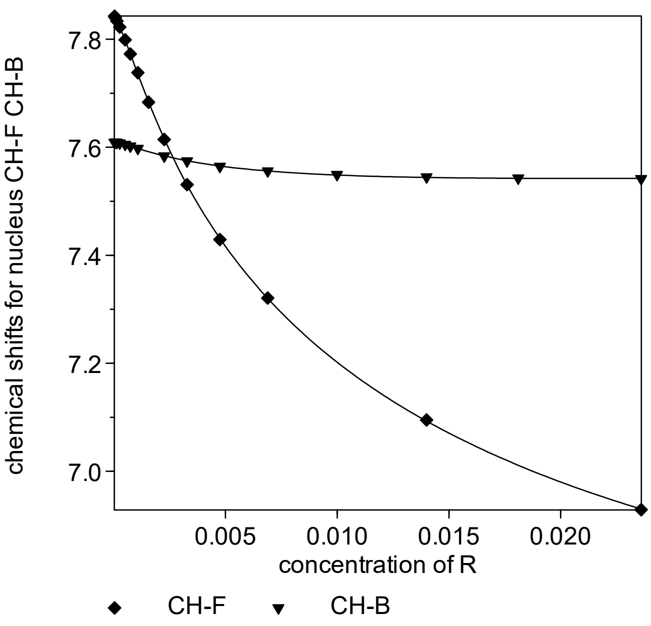

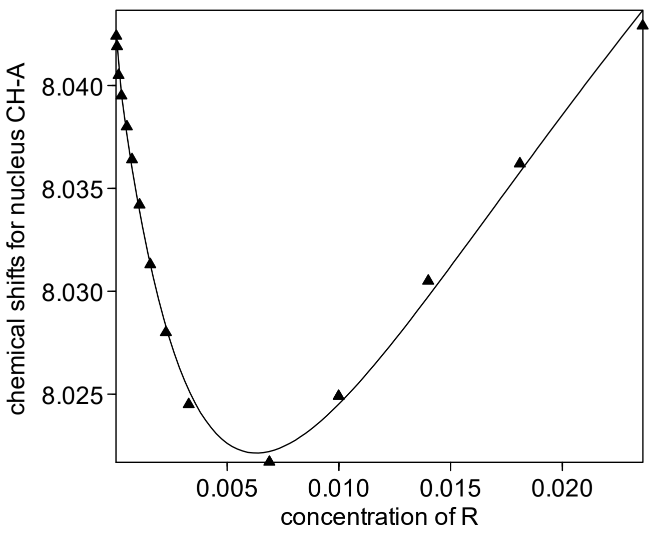

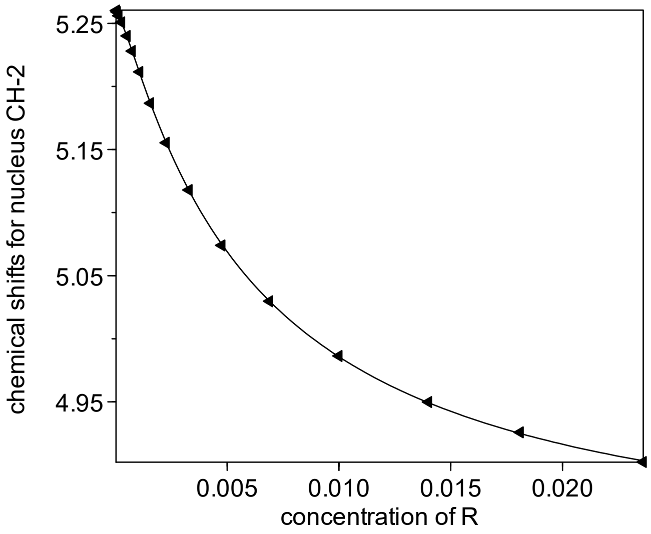

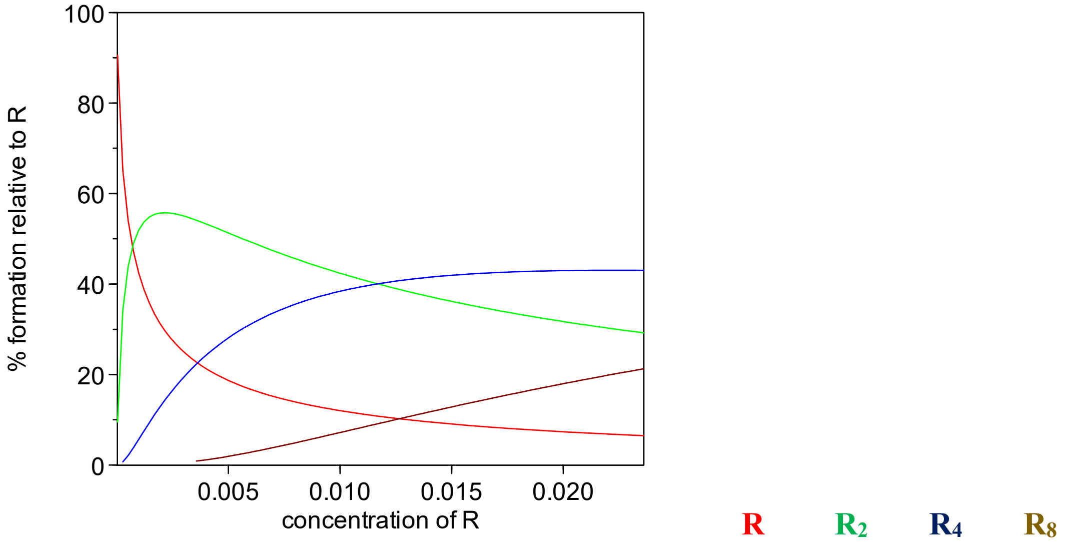

1 + MeβCeB (D_2O, pD 7.4, 298 K, 500 MHz).

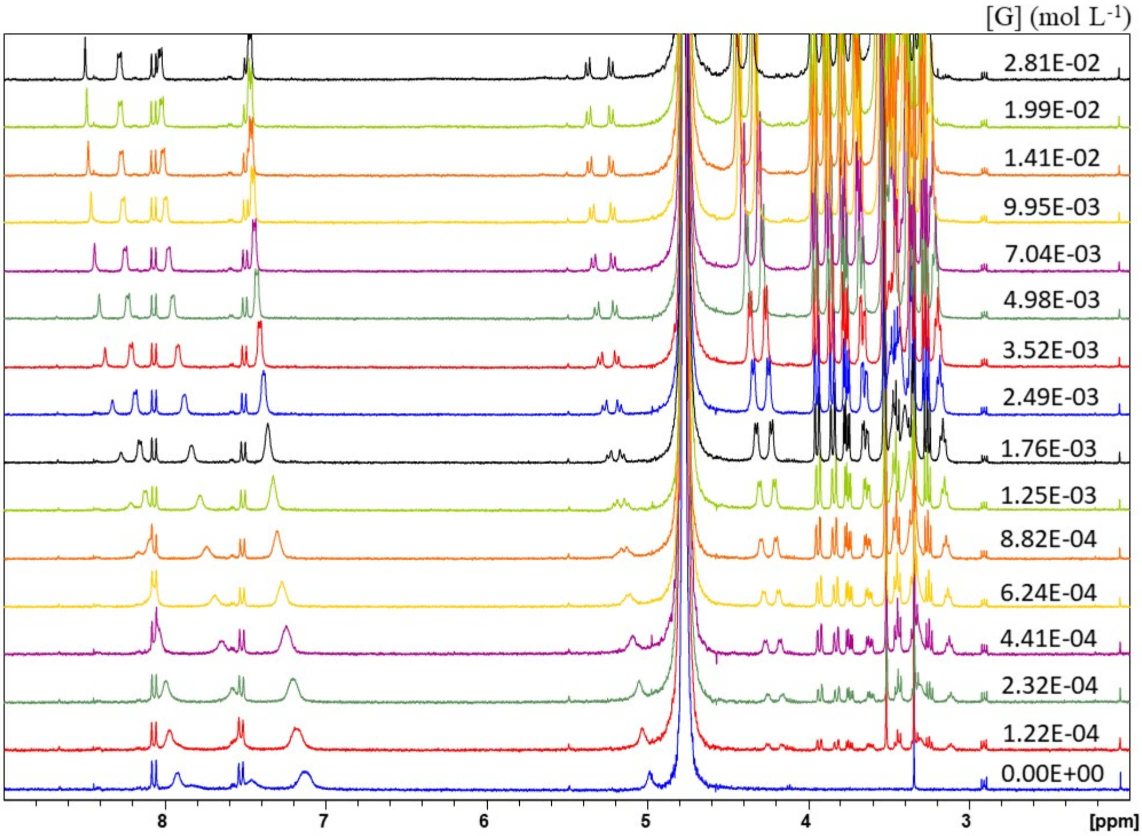

Figure S8. ^{1}H NMR spectroscopic titration (500 MHz, D_2O, pD 7.4, 298 K) of receptor **1** ($5.06\ 10^{-4}$ mol L^{-1}) with incremental concentrations of MeβCeB (G).

Data Table

R = **1**   G = MeβCeB

δ (ppm) *vs*. [G] (mol L^{-1})

Titration [R] = 5.06 10^{-4} mol L^{-1}

[G]	CH'-1 G	CH-1 G	CH-6 G	CH'-6 G	CH-6' G	CH'-6' G	CH-2 G	CH-G R	CH-C R	CH-F R
0.00E+00	-	-	-	-	-	-	-	7.8357	7.9226	7.4617
1.22E-04	4.2508	4.1612	3.9418	3.8358	3.7604	3.6318	3.1133	-	7.9705	-
2.32E-04	4.2530	4.1639	3.9418	3.8354	3.7599	3.6312	3.1139	-	7.9945	7.5777
4.41E-04	4.2692	4.1774	3.9444	3.8405	3.7638	3.6385	3.1248	-	8.0373	7.6477
6.24E-04	4.2794	4.1871	3.9469	3.8435	3.7664	3.6425	3.1326	-	8.0729	7.6892
8.82E-04	4.2924	4.1995	3.9502	3.8483	3.7695	3.6487	3.1425	8.1669	8.0976	7.7411
1.25E-03	4.3049	4.2120	3.9531	3.8530	3.7732	3.6545	3.1527	8.2124	8.1238	7.7821
1.76E-03	4.3234	4.2288	3.9570	3.8590	3.7776	3.6624	3.1661	8.2723	8.1561	7.8338
2.49E-03	4.3427	4.2468	3.9612	3.8651	3.7826	3.6708	3.1802	8.3257	8.1848	7.8790
3.52E-03	4.3638	4.2676	3.9659	3.8727	3.7883	3.6791	3.1965	8.3715	8.2095	7.9201
4.98E-03	4.3846	4.2869	3.9701	3.8797	3.7931	3.6904	3.2115	8.4085	8.2300	7.9512
7.04E-03	4.4044	4.3052	3.9746	3.8864	3.7975	3.6993	3.2262	8.4373	8.2470	7.9770
9.95E-03	4.4222	4.3221	3.9789	3.8926	3.8012	3.7077	3.2393	8.4594	8.2603	7.9972
1.41E-02	4.4380	4.3368	3.9820	3.8976	3.8041	3.7150	3.2509	8.4757	8.2704	8.0126
1.99E-02	4.4478	4.3460	3.9846	3.9010	3.8080	3.7195	3.2585	8.4863	8.2775	8.0219
2.81E-02	4.4597	4.3574	3.9867	3.9051	3.8122	3.7247	3.2678	8.4964	8.2828	8.0316

Results page

```
no. of spectra            16
no. of resonance values  148
no. of resonant nuclei    10

  sigma =   0.00181807032          RMS weighted residual =   0.00158861640
```

stoich coeff				value	relative std devn	log beta	standard deviation	
Beta	0	2	constant	4.4208E+002		2.6455		(R2)
Beta	1	1	refined	3.4165E+002	0.1680	2.5336	0.0730	(GR)
Beta	1	2	refined	2.1265E+006	0.1447	6.3277	0.0628	(GR2)

Individual chemical shifts

	+	G value	G error	R value	R error
CH'-1	+	4.5038	0.0044		
CH-1	+	4.3992	0.0042		
CH-6	+	3.9969	0.0019		
CH'-6	+	3.9205	0.0022		
CH-6'	+	3.8219	0.0020		
CH'-6'	+	3.7458	0.0026		
CH-2	+	3.3007	0.0035		
CH-G	+			7.5399	0.1320
CH-C	+			7.7988	0.0778
CH-F	+			7.2183	0.1163
	+				

	+	0,2 value	0,2 error	1,1 value	1,1 error
CH'-1	+			0.7438	0.6846
CH-1	+			0.8403	0.6484
CH-6	+			3.1554	0.1679
CH'-6	+			2.6206	0.2457
CH-6'	+			2.8986	0.1814
CH'-6'	+			1.9671	0.3298
CH-2	+			0.4787	0.5160
CH-G	+	8.7195	0.3943	8.4876	0.0163
CH-C	+	8.3034	0.2330	8.2915	0.0082
CH-F	+	8.1956	0.3475	8.0330	0.0134
	+				

	+	1,2 value	1,2 error
CH'-1	+	4.8811	0.0342
CH-1	+	4.7639	0.0344
CH-6	+	4.0873	0.0315
CH'-6	+	4.0621	0.0319
CH-6'	+	3.9142	0.0314
CH'-6'	+	3.9525	0.0331
CH-2	+	3.5928	0.0334
CH-G	+	8.7434	0.0238
CH-C	+	8.3830	0.0199
CH-F	+	8.2233	0.0227

Correlation coefficients*1000

	1	2
1		
2	-255	

Parameters are numbered as follows
1 beta 1,1
2 beta 1,2

Titration Plots

Chemical shifts (δ, ppm) *vs*. concentration of G (mol L^{-1})

experimental (symbols) and calculated (lines) values

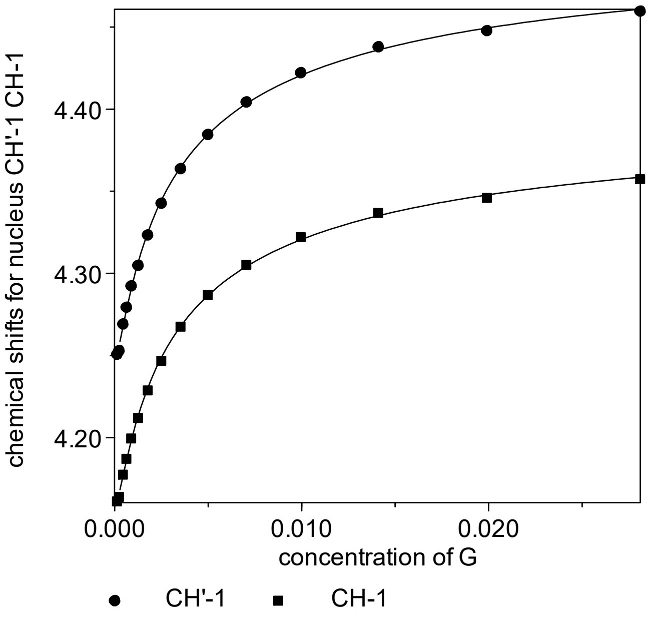

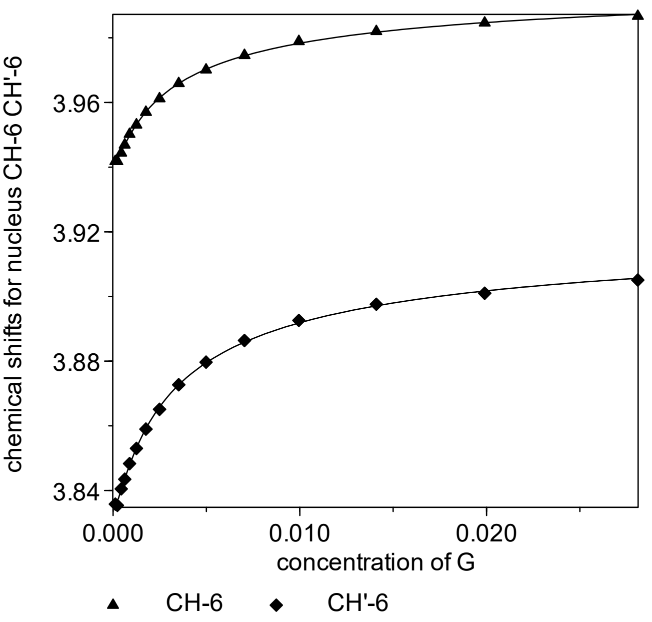

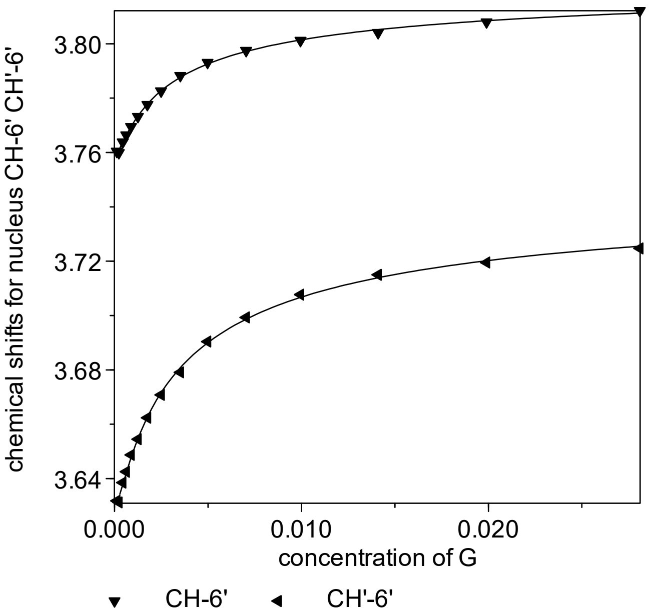

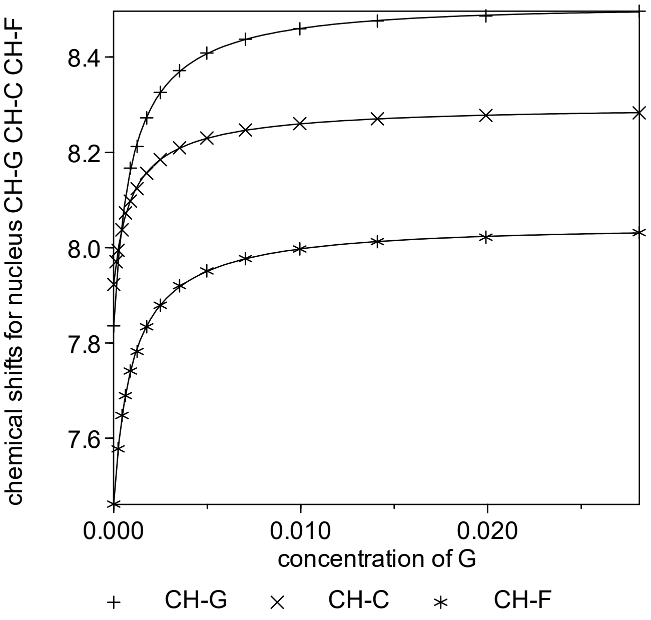

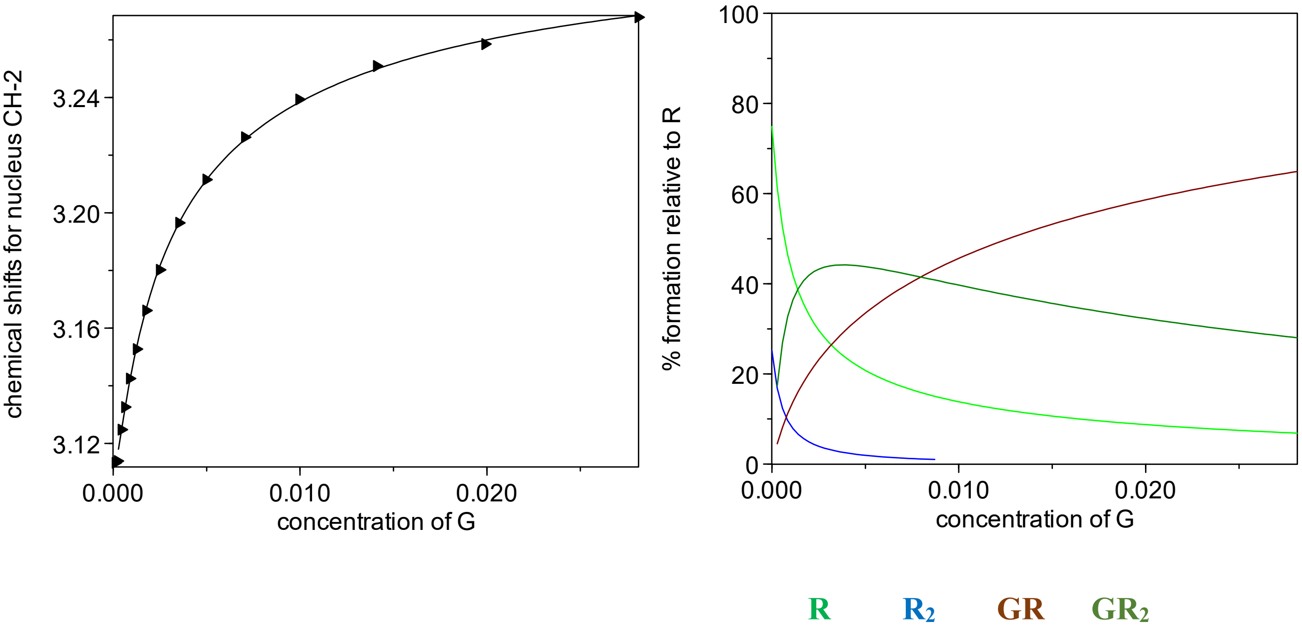
chemical shifts for nucleus CH-2
3.24
3.20
3.16
3.12
0.000
0.010
0.020
concentration of G
% formation relative to R
100
80
60
40
20
0
0.000
0.010
0.020
concentration of G
R
R2
GR
GR2

1 + MeβCeB (D_2O, pD 11, 298 K, 500 MHz).

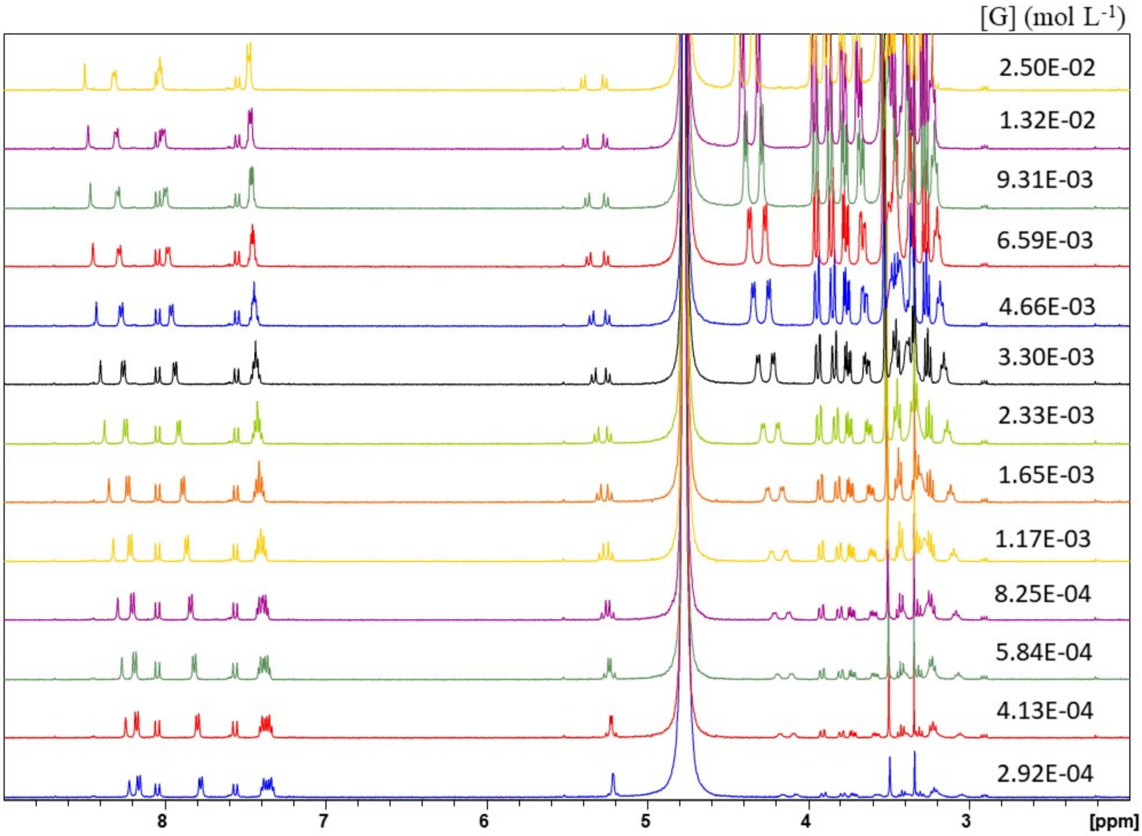

Figure S9. ^1H NMR spectroscopic titration (500 MHz, D_2O, pD 11, 298 K) of receptor **1** ($5.55\ 10^{-4}$ mol L^{-1}) with incremental concentrations of MeβCeB (G).

Data Table

R = **1** G = MeβCeB

δ (ppm) *vs*. [G] (mol L^{-1})

Titration [R] = 5.55 10^{-4} mol L^{-1}

[G]	CH'-1 G	CH-1 G	CH-6 G	CH'-6 G	CH-6' G	CH'-6' G	CH-2 G	CH-G R	CH-C R	CH-F R
2.92E-04	4.1655	4.0841	3.9193	3.7996	3.7347	3.5884	3.0446	8.2206	8.1711	7.7858
4.13E-04	4.1793	4.0969	3.9233	3.8054	3.7388	3.5960	3.0559	8.2429	8.1833	7.8045
5.84E-04	4.1946	4.1104	3.9266	3.8100	3.7428	3.6031	3.0667	8.2665	8.1965	7.8252
8.25E-04	4.2150	4.1277	3.9307	3.8170	3.7475	3.6110	3.0812	8.2929	8.2097	7.8477
1.17E-03	4.2372	4.1492	3.9356	3.8244	3.7534	3.6213	3.0975	8.3201	8.2245	7.8718
1.65E-03	4.2624	4.1724	3.9410	3.8331	3.7594	3.6325	3.1163	8.3476	8.2385	7.8966
2.33E-03	4.2902	4.1988	3.9476	3.8431	3.7666	3.6448	3.1360	8.3755	8.2532	7.9221
3.30E-03	4.3196	4.2261	3.9539	3.8529	3.7740	3.6576	3.1582	8.4005	8.2666	7.9459
4.66E-03	4.3494	4.2535	3.9604	3.8627	3.7820	3.6692	3.1796	8.4260	8.2806	7.9691
6.59E-03	4.3761	4.2791	3.9663	3.8722	3.7885	3.6808	3.1996	8.4464	8.2919	7.9893
9.31E-03	4.3999	4.3019	3.9714	3.8808	3.7942	3.6938	3.2177	8.4632	8.3006	8.0054
1.32E-02	4.4204	4.3210	3.9761	3.8880	3.7983	3.7033	3.2323	8.4768	8.3080	8.0186
2.50E-02	4.4529	4.3511	3.9829	3.8992	3.8054	3.7182	3.2563	8.4969	8.3209	8.0309

Results page

no. of spectra 14
no. of resonance values 130
no. of resonant nuclei 10

Chi-squared = 6.98

sigma = 0.00097912641 RMS weighted residual = 0.00083700662

	stoich coeff			value	relative std devn	log beta	standard deviation	
Beta	0	2	constant	1.4666E+003		3.1663		(R2)
Beta	1	1	refined	3.1598E+002	0.1775	2.4997	0.0771	(GR)
Beta	1	2	refined	2.8496E+006	0.2383	6.4548	0.1035	(GR2)

Individual chemical shifts

		G		R	
	+	value	error	value	error
CH'-1	+	4.5196	0.0058		
CH-1	+	4.4154	0.0055		
CH-6	+	3.9975	0.0019		
CH'-6	+	3.9225	0.0024		
CH-6'	+	3.8215	0.0020		
CH'-6'	+	3.7481	0.0029		
CH-2	+	3.3058	0.0044		
CH-G	+			7.9930	0.2024
CH-C	+			8.0909	0.1167
CH-F	+			7.3215	0.1554

		0,2		1,1	
	+	value	error	value	error
CH'-1	+			-0.9746	1.1777
CH-1	+			-0.8523	1.1225
CH-6	+			2.8082	0.2650
CH'-6	+			1.9944	0.4170
CH-6'	+			2.5673	0.2828
CH'-6'	+			1.2173	0.5421
CH-2	+			-0.7689	0.8729
CH-G	+	8.3548	0.2306	8.5330	0.0093
CH-C	+	8.1952	0.1337	8.3491	0.0071
CH-F	+	8.2206	0.1799	8.0364	0.0121

		1,2	
	+	value	error
CH'-1	+	4.5074	0.1530
CH-1	+	4.4573	0.1320
CH-6	+	3.9878	0.0435
CH'-6	+	3.9309	0.0559
CH-6'	+	3.7791	0.0527
CH'-6'	+	3.7742	0.0659
CH-2	+	3.3105	0.1110
CH-G	+	8.5420	0.0368
CH-C	+	8.3256	0.0253
CH-F	+	8.1530	0.0192

Correlation coefficients*1000

	1	2
1		
2	-79	

Parameters are numbered as follows
1 beta 1,1
2 beta 1,2

Titration Plots

Chemical shifts (δ, ppm) *vs*. concentration of G (mol L^{-1})

experimental (symbols) and calculated (lines) values

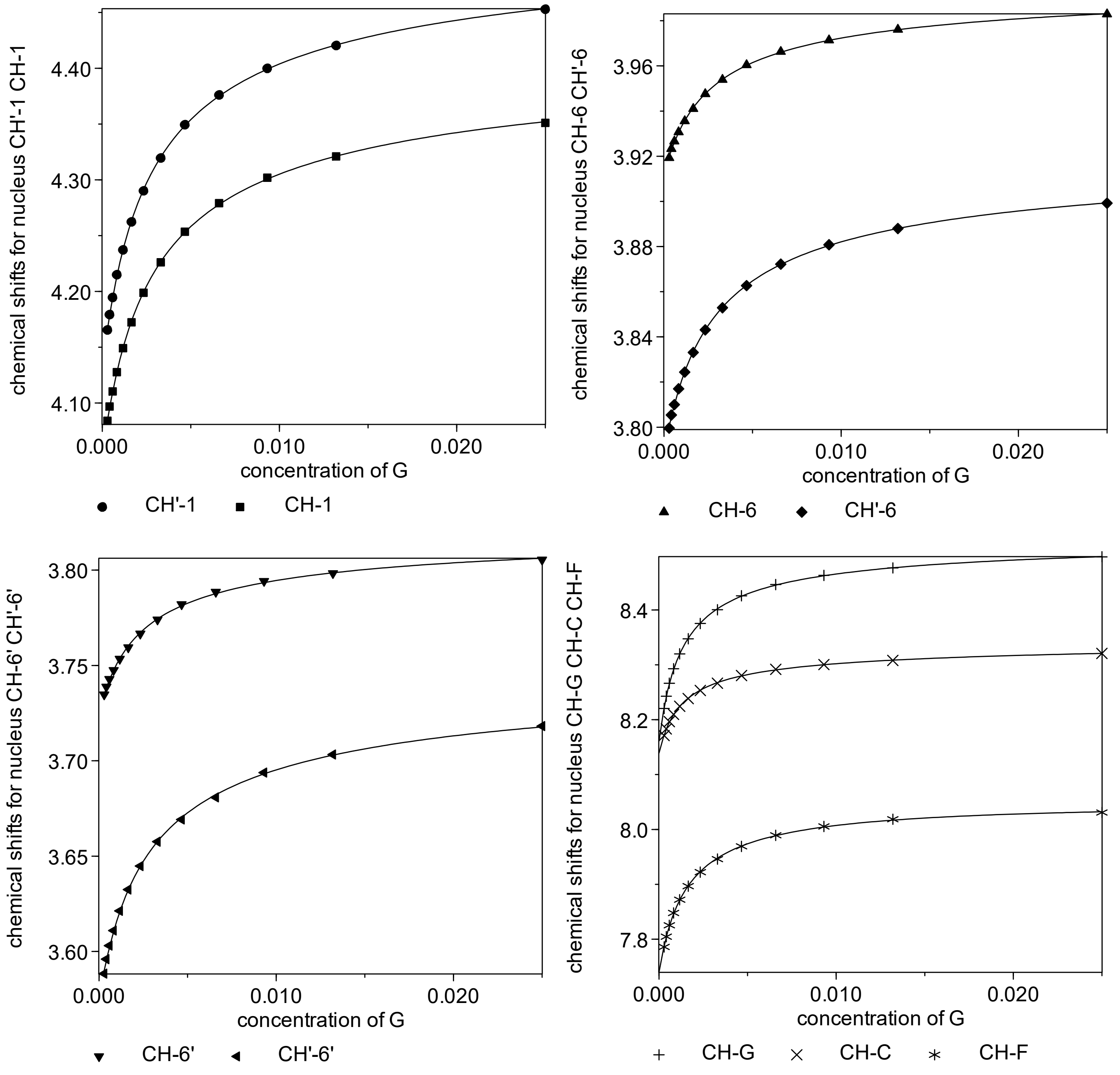

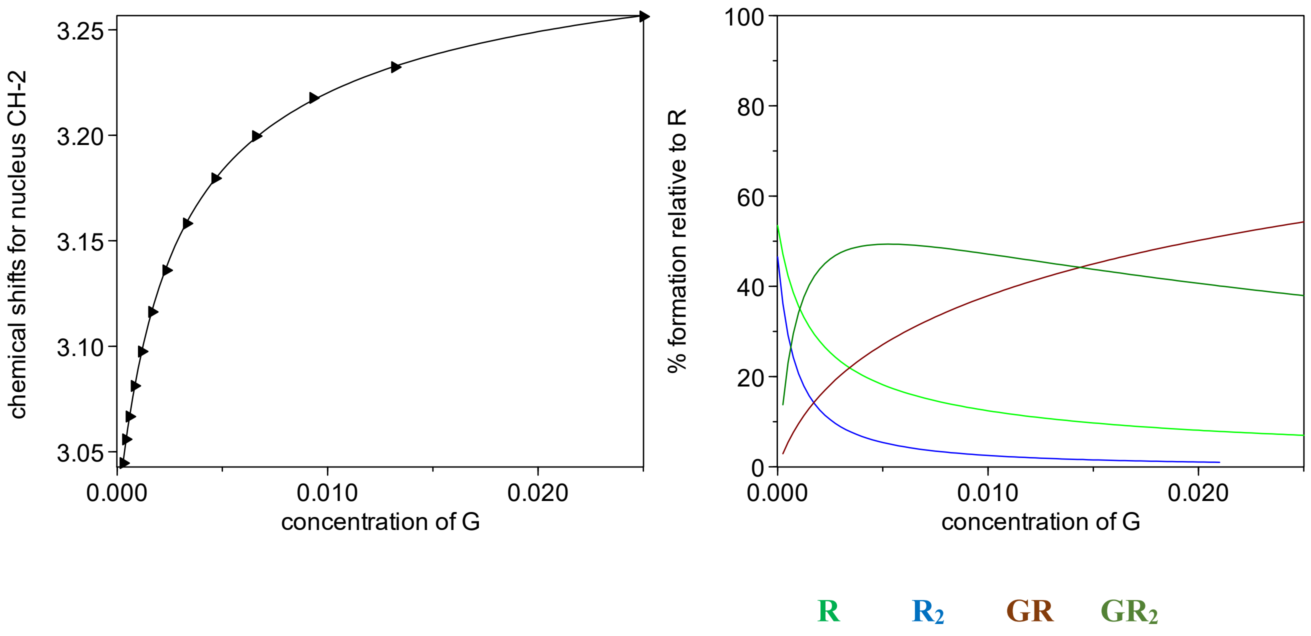
3.25
3.20
3.15
3.10
3.05
chemical shifts for nucleus CH-2
0.000
0.010
0.020
concentration of G
100
80
60
40
20
0
% formation relative to R
0.000
0.010
0.020
concentration of G
R
R2
GR
GR2

1 + MeβMal (D_2O, pD 7.4, 298 K, 500 MHz).

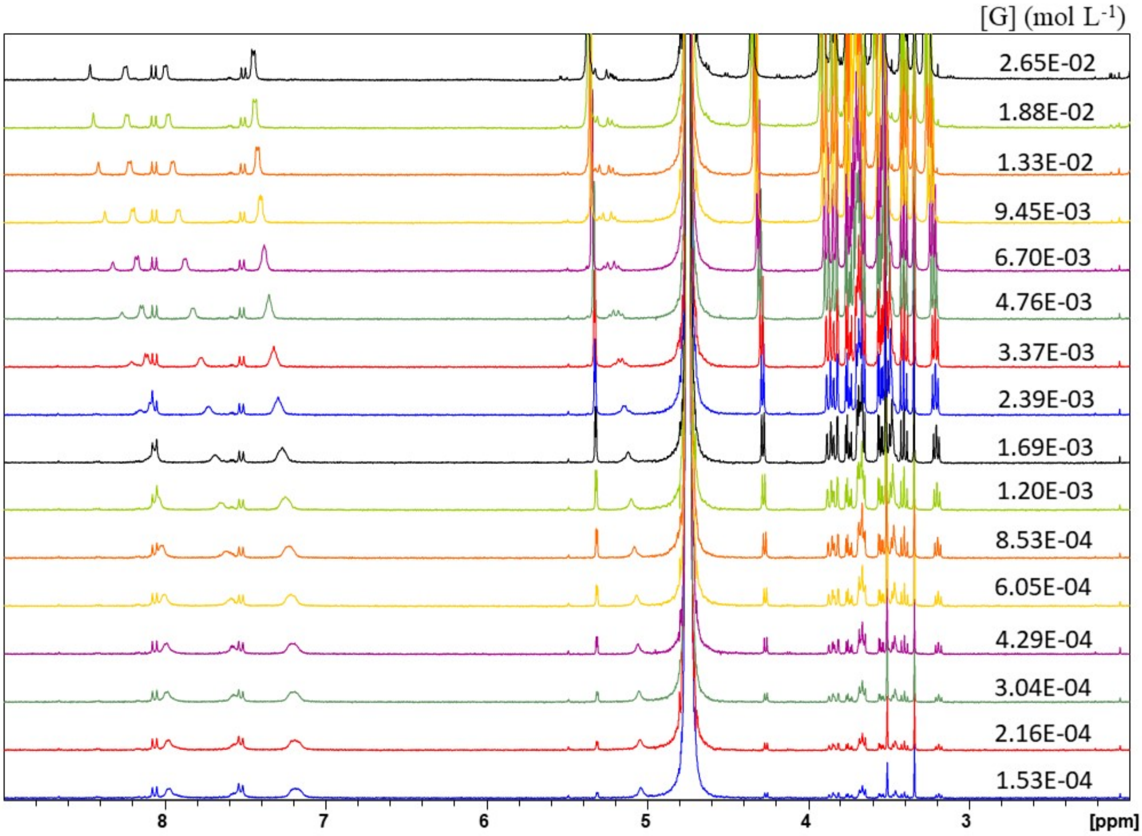

Figure S10. ^{1}H NMR spectroscopic titration (500 MHz, D_2O, pD 7.4, 298 K) of receptor **1** (4.01 10^{-4} mol L^{-1}) with incremental concentrations of MeβMal (G).

Data Table

R = **1** G = MeβMal

δ (ppm) *vs*. [G] (mol L^{-1})

Titration [R] = 4.01 10^{-4} mol L^{-1}

[G]	CH'-1 G	CH-1 G	CH-6 G	CH'-6 G	CH-6' G	CH'-6' G	CH-2 G	CH-G R	CH-C R	CH-F R
1.53E-04	5.3133	4.2729	3.8724	3.8149	3.5109	3.1897	7.9759	-	7.5625	7.1870
2.16E-04	5.3147	4.2739	3.8730	3.8150	3.5114	3.1903	7.9882	-	7.5692	7.1923
3.04E-04	5.3152	4.2746	3.8736	3.8154	3.5118	3.1911	7.9883	-	7.5738	7.2025
4.29E-04	5.3163	4.2761	3.8749	3.8156	3.5125	3.1927	7.9931	-	7.5810	7.2055
6.05E-04	5.3175	4.2778	3.8758	3.8157	3.5132	3.1939	8.0041	-	7.5882	7.2162
8.53E-04	5.3190	4.2801	3.8771	3.8162	3.5142	3.1963	8.0208	-	7.6147	7.2284
1.20E-03	5.3219	4.2844	3.8802	3.8168	3.5163	3.1996	-	-	7.6505	7.2498
1.69E-03	5.3251	4.2890	3.8831	3.8178	3.5184	3.2033	-	8.1146	7.6874	7.2702
2.39E-03	5.3290	4.2945	3.8867	3.8189	3.5210	3.2080	8.0880	8.1537	7.7284	7.2952
3.37E-03	5.3336	4.3013	3.8914	3.8199	3.5240	3.2139	8.1151	8.2074	7.7767	7.3240
4.76E-03	5.3396	4.3099	3.8967	3.8216	3.5279	3.2209	8.1452	8.2669	7.8258	7.3533
6.70E-03	5.3463	4.3196	3.9029	3.8232	3.5323	3.2289	8.1734	8.3227	7.8748	7.3815
9.45E-03	5.3539	4.3308	3.9103	3.8251	3.5373	3.2383	8.1988	8.3729	7.9173	7.4057
1.33E-02	5.3618	4.3419	3.9175	3.8274	3.5425	3.2474	8.2204	8.4142	7.9523	7.4251
1.88E-02	5.3685	4.3517	3.9236	3.8287	3.5467	3.2559	8.2355	8.4441	7.9787	7.4406
2.65E-02	5.3739	4.3597	3.9289	3.8303	3.5502	3.2623	8.2465	8.4650	7.9971	7.4501

Results page

```
no. of spectra             16
no. of resonance values   151
no. of resonant nuclei     10

  sigma =   0.00194509826          RMS weighted residual =   0.00177679918

      stoich            value   relative    log    standard
      coeff                     std devn    beta   deviation

Beta  1  1 refined  1.8579E+002  0.0334    2.2690   0.0145  (   GR   )
Beta  0  2 constant 4.4208E+002            2.6455           (   R2   )

Individual chemical shifts

                  G                        R
=========================================================
       +       value    error         value    error
CH'-1  +      5.3910   0.0017
CH-1   +      4.3842   0.0019
CH-6   +      3.9449   0.0017
CH'-6  +      3.8347   0.0015
CH3    +      3.5615   0.0016
CH-2   +      3.2830   0.0018
CH-C   +                              8.2716   0.0258
CH-G   +                              8.3829   0.0383
CH-F   +                              7.9889   0.0387
CH-B   +                              7.4950   0.0258
       +
                 1,1                       0,2
=========================================================
       +       value    error         value    error
CH'-1  +      3.9648   0.0662
CH-1   +      2.3238   0.0890
CH-6   +      2.6062   0.0632
CH'-6  +      3.4697   0.0365
CH3    +      2.6269   0.0502
CH-2   +      1.5599   0.0766
CH-C   +      8.2561   0.0048         6.8802   0.0934
CH-G   +      8.5083   0.0059         6.4217   0.1399
CH-F   +      8.0230   0.0061         5.9277   0.1386
CH-B   +      7.4560   0.0048         6.0455   0.0934
```

Titration Plots

Chemical shifts (δ, ppm) *vs*. concentration of G (mol L^{-1})

experimental (symbols) and calculated (lines) values

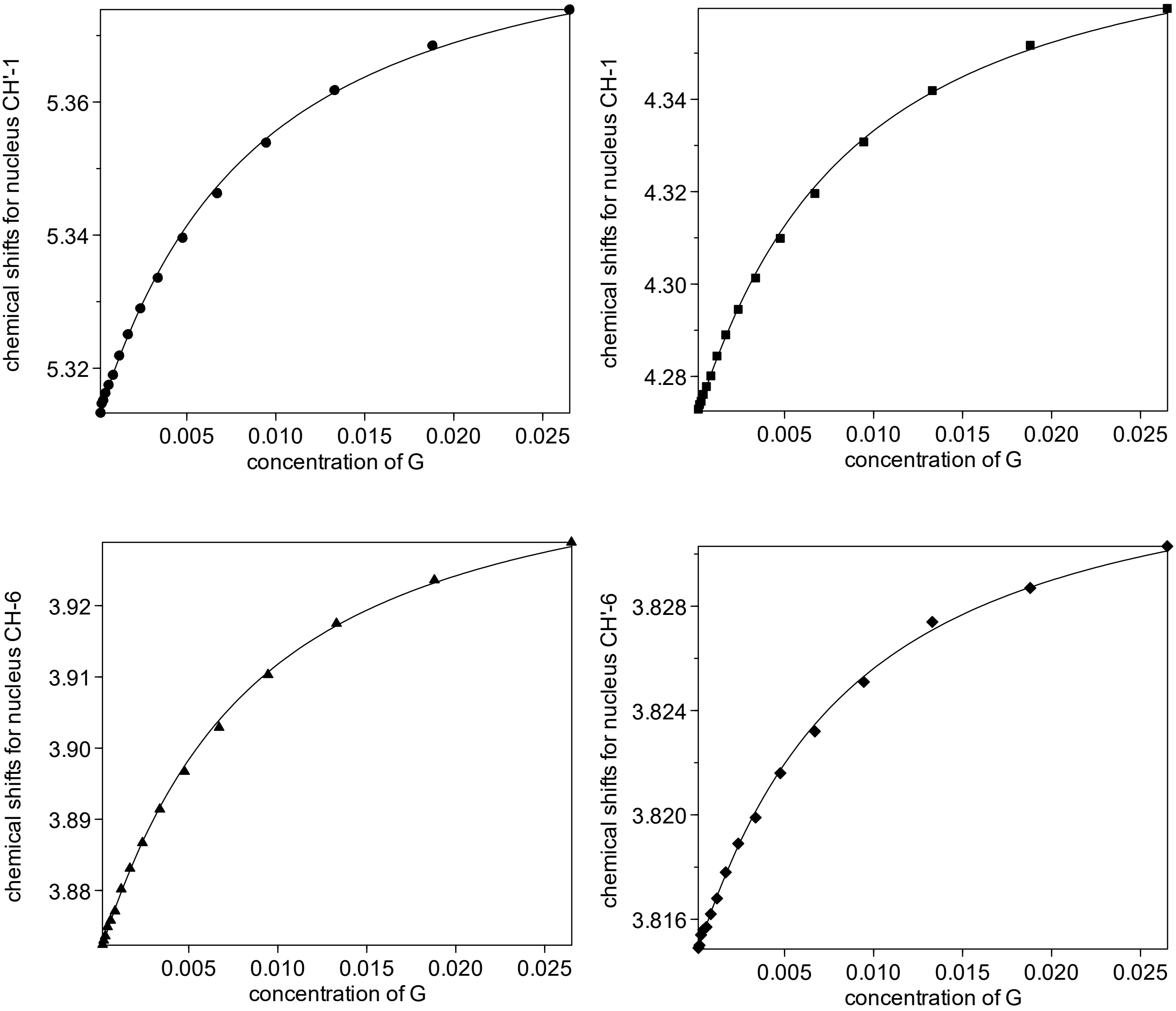

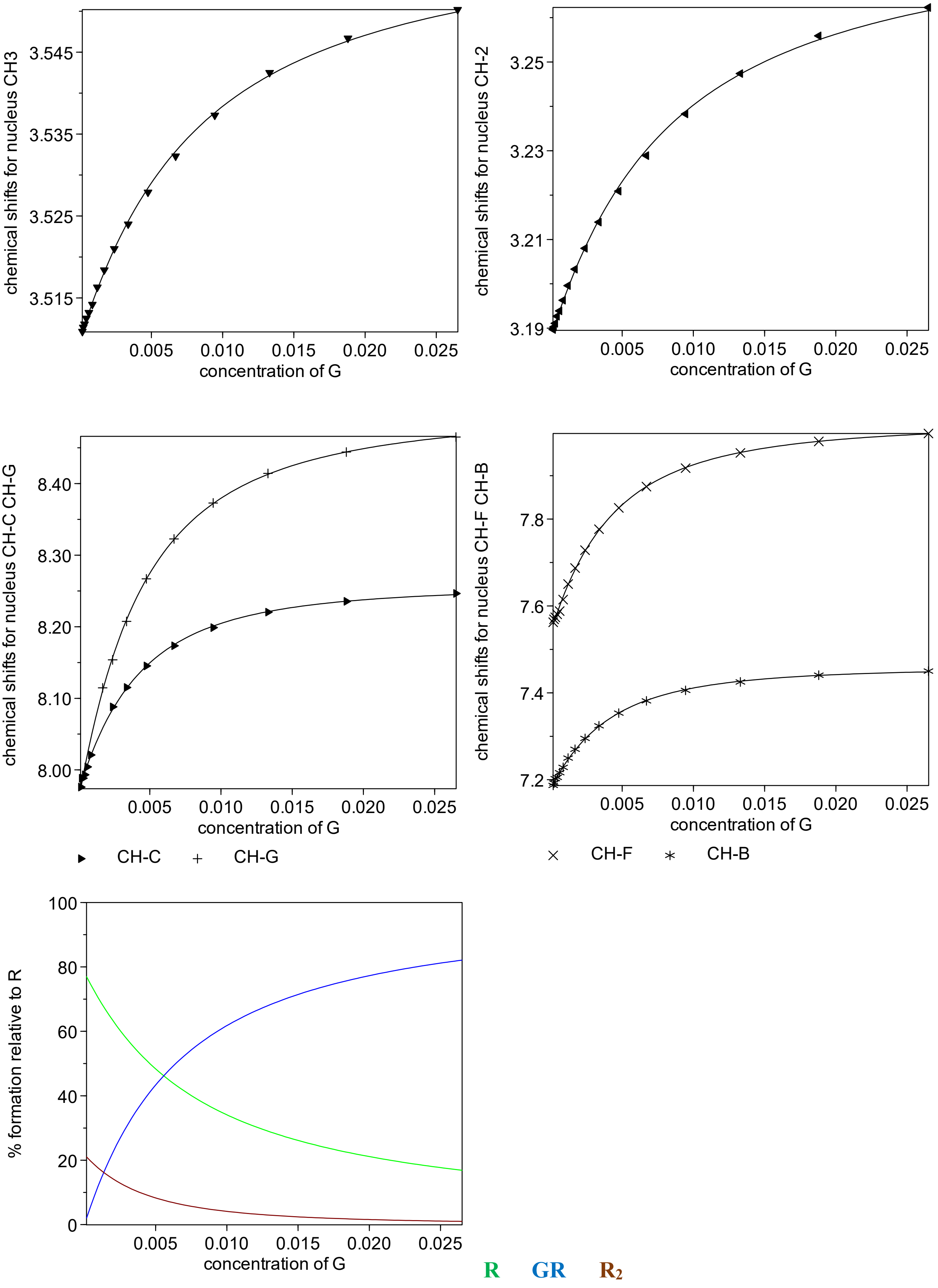
chemical shifts for nucleus CH3
concentration of G
chemical shifts for nucleus CH-2
concentration of G
chemical shifts for nucleus CH-C CH-G
concentration of G
CH-C
CH-G
chemical shifts for nucleus CH-F CH-B
concentration of G
CH-F
CH-B
% formation relative to R
concentration of G
R
GR
R2

1 + MeβLac (D_2O, pD 7.4, 298 K, 500 MHz).

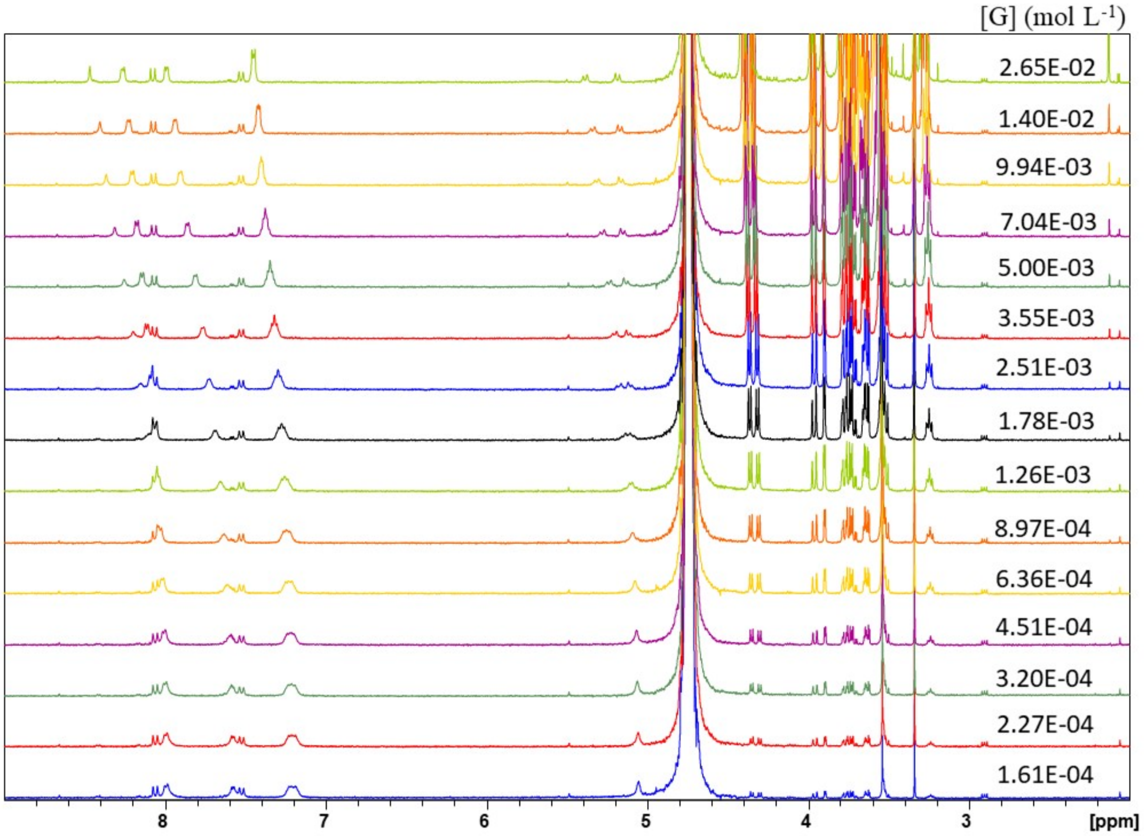

Figure S11. ^{1}H NMR spectroscopic titration (500 MHz, D_2O, pD 7.4, 298 K) of receptor **1** (4.22 10^{-4} mol L^{-1}) with incremental concentrations of MeβLac (G).

Data Table

R = **1**   G = MeβLac

δ (ppm) *vs*. [G] (mol L^{-1})

Titration [R] = 4.22 10^{-4} mol L^{-1}

[G]	CH'-1<br>G	CH-1<br>G	CH-6<br>G	CH'-4<br>G	CH3<br>G	CH-2<br>G	CH-C<br>R	CH-G<br>R	CH-F<br>R
1.61E-04	4.3631	4.3134	3.9732	3.8984	3.5420	3.2404	7.9943	-	7.5810
2.27E-04	4.3635	4.3139	3.9738	3.8986	3.5421	3.2412	7.9965	-	7.5811
3.20E-04	4.3639	4.3146	3.9740	3.8987	3.5423	3.2418	8.0020	-	7.5849
4.51E-04	4.3647	4.3155	3.9742	3.8989	3.5425	3.2424	8.0078	-	7.5916
6.36E-04	4.3661	4.3170	3.9745	3.8991	3.5429	3.2431	8.0178	-	7.6140
8.97E-04	4.3679	4.3189	3.9749	3.8994	3.5434	3.2443	8.0304	-	7.6329
1.26E-03	4.3698	4.3211	3.9753	3.9000	3.5440	3.2459	-	-	7.6567
1.78E-03	4.3727	4.3243	3.9760	3.9007	3.5449	3.2481	-	8.1005	7.6896
2.51E-03	4.3764	4.3284	3.9772	3.9015	3.5461	3.2509	8.0897	8.1544	7.7264
3.55E-03	4.3809	4.3333	3.9779	3.9026	3.5474	3.2539	8.1152	8.1998	7.7679
5.00E-03	4.3868	4.3401	3.9801	3.9040	3.5491	3.2586	8.1450	8.2565	7.8135
7.04E-03	4.3936	4.3477	3.9817	3.9055	3.5511	3.2635	8.1763	8.3132	7.8618
9.94E-03	4.4013	4.3561	3.9837	3.9071	3.5533	3.2690	8.2050	8.3671	7.9065
1.40E-02	4.4077	4.3633	3.9853	3.9086	3.5552	3.2737	8.2272	8.4065	7.9407
2.65E-02	4.4200	4.3770	3.9884	3.9112	3.5586	3.2828	8.2644	8.4694	7.9930

Results page

```
no. of spectra             15
no. of resonance values  126
no. of resonant nuclei     9

  sigma =   0.00148779121          RMS weighted residual =   0.00135167844
```

	stoich coeff			value	relative std devn	log beta	standard deviation	
Beta	1	1	refined	1.8642E+002	0.0450	2.2705	0.0195	(GR)
Beta	0	2	constant	4.4208E+002		2.6455		(R2)

Individual chemical shifts

		G value	G error	R value	R error
CH'-1	+	4.4349	0.0017		
CH-1	+	4.3936	0.0018		
CH-6	+	3.9924	0.0013		
CH'-4	+	3.9147	0.0013		
CH3	+	3.5632	0.0013		
CH-2	+	3.2938	0.0015		
CH-C	+			8.1092	0.0283
CH-G	+			8.2328	0.0459
CH-F	+			7.8012	0.0435
	+				

		1,1 value	1,1 error	0,2 value	0,2 error
CH'-1	+	3.1512	0.0752		
CH-1	+	2.9625	0.0828		
CH-6	+	3.6562	0.0326		
CH'-4	+	3.6244	0.0313		
CH3	+	3.1858	0.0339		
CH-2	+	2.3484	0.0583		
CH-C	+	8.3035	0.0038	7.5589	0.0978
CH-G	+	8.5352	0.0052	7.0000	0.1556
CH-F	+	8.0467	0.0052	6.7354	0.1496

Titration Plots

Chemical shifts (δ, ppm) *vs*. concentration of G (mol L^{-1})

experimental (symbols) and calculated (lines) values

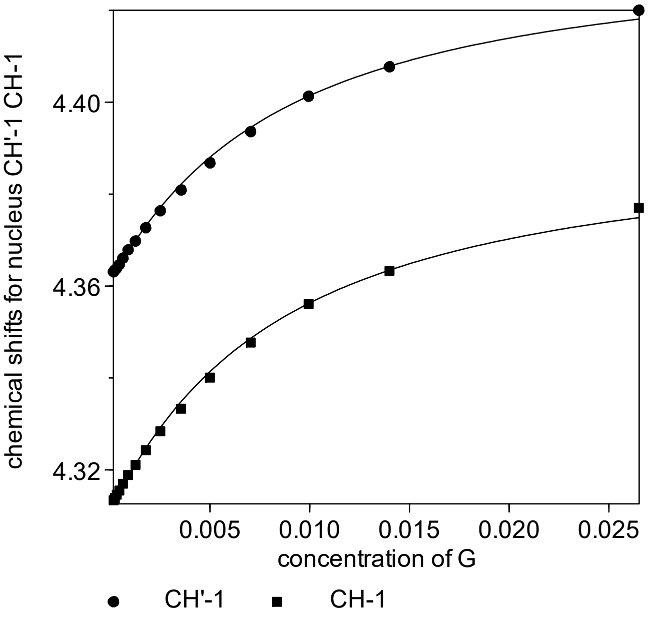

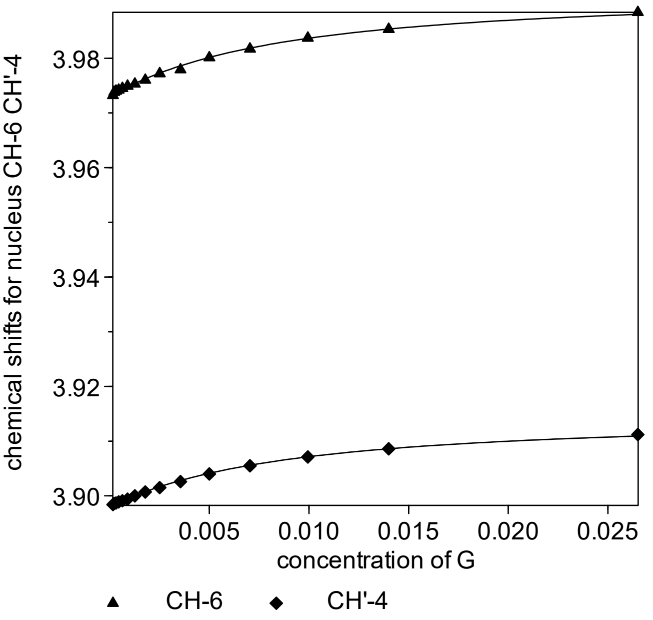

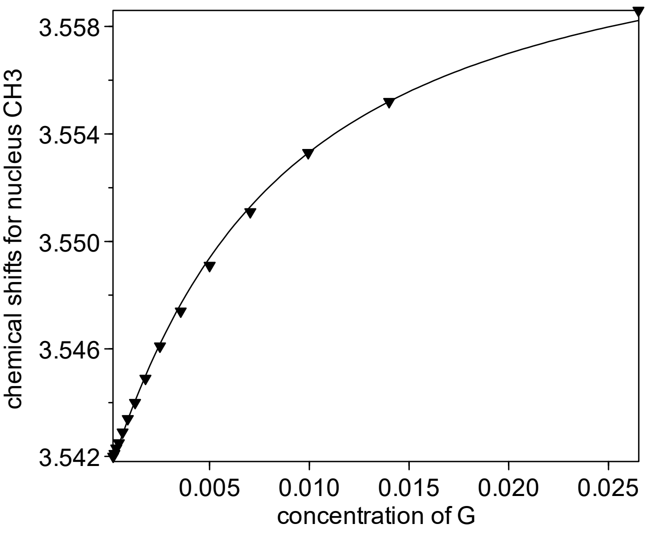

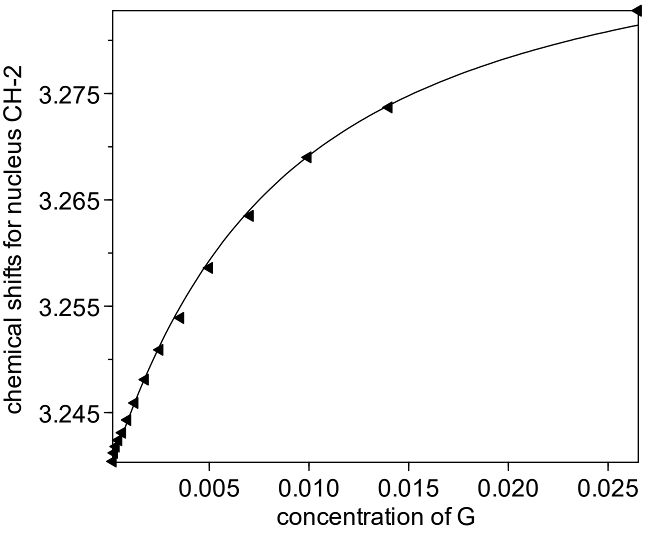

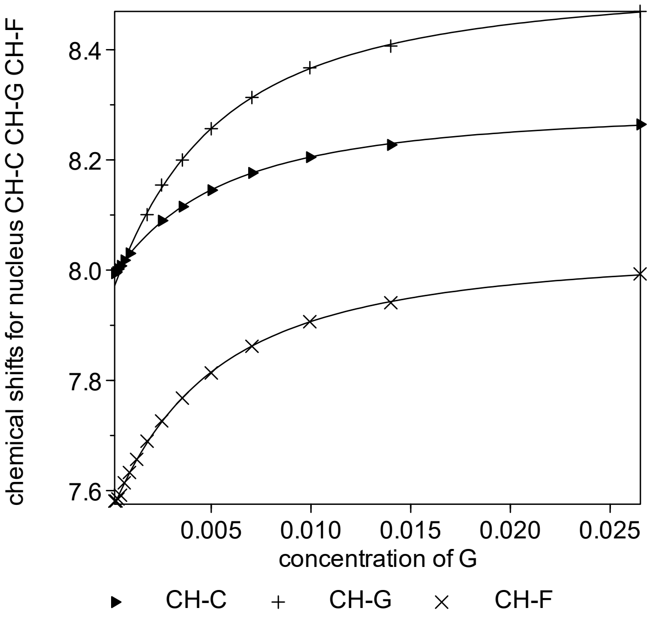
chemical shifts for nucleus CH-C CH-G CH-F
8.4
8.2
8.0
7.8
7.6
0.005
0.010
0.015
0.020
0.025
concentration of G
CH-C
CH-G
CH-F

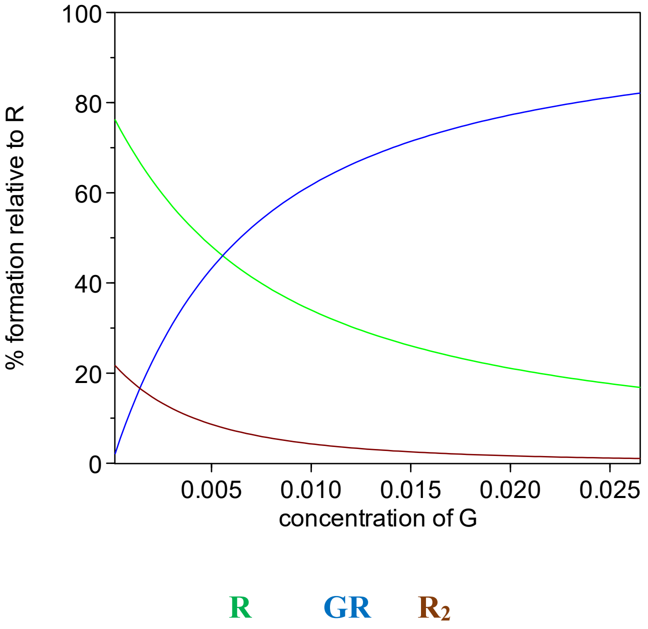
% formation relative to R
100
80
60
40
20
0
0.005
0.010
0.015
0.020
0.025
concentration of G
R
GR
R2

1 + MeβGlcNAc$_2$ (D_2O, pD 7.4, 298 K, 500 MHz).

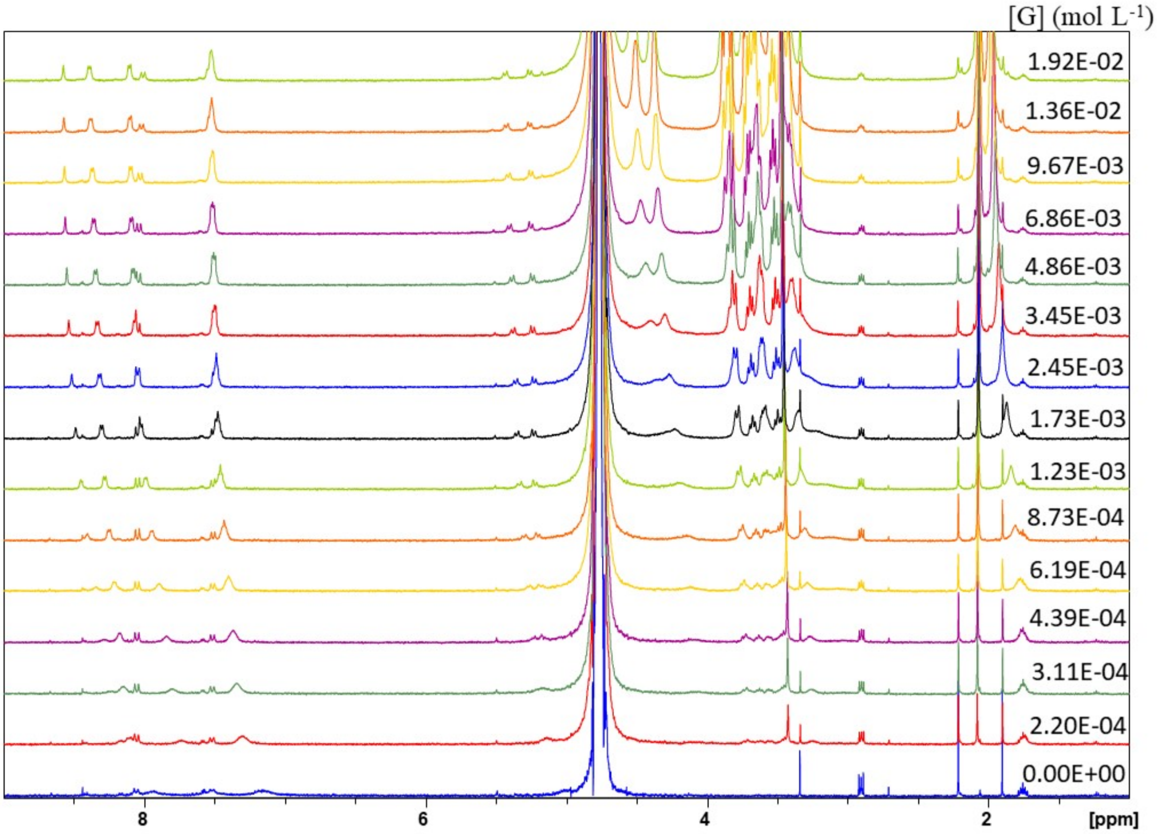

Figure S12. ^{1}H NMR spectroscopic titration (500 MHz, D_2O, pD 7.4, 298 K) of receptor **1** (5.46 10^{-4} mol L^{-1}) with incremental concentrations of MeβGlcNAc$_2$ (G).

Data Table

R = **1**   G = MeβGlcNAc$_2$

δ (ppm) *vs*. [G] (mol L^{-1})

Titration [R] = 5.46 10^{-4} mol L^{-1}

[G]	CH-1 G	CH-6' G	CH3 G	Ac' G	Ac G	CH-C R	CH-G R
0.00E+00	-	-	-	-	-	7.9463	7.5285
2.20E-04	4.0751	3.7183	3.4293	2.0829	-	8.1064	-
3.11E-04	4.0774	3.7234	3.4319	2.0821	-	8.1513	-
4.39E-04	4.0908	3.7287	3.4343	2.0812	-	8.1789	-
6.19E-04	4.1191	3.7374	3.4387	2.0799	1.7809	8.2139	7.8962
8.73E-04	-	3.7497	3.4441	2.0781	1.8123	8.2540	7.9495
1.23E-03	4.1978	3.7621	3.4503	2.0760	1.8419	8.2841	7.9918
1.73E-03	4.2313	3.7775	3.4570	2.0737	1.8736	8.3045	8.0259
2.45E-03	4.2733	3.7913	3.4635	2.0715	1.9043	8.3205	8.0514
3.45E-03	4.3004	3.8017	3.4688	2.0698	1.9284	8.3343	8.0706
4.86E-03	4.3302	3.8107	3.4731	2.0684	1.9493	8.3481	8.0840
6.86E-03	4.3504	3.8186	3.4767	2.0672	1.9660	8.3613	8.0938
9.67E-03	4.3684	3.8236	3.4796	2.0662	1.9787	8.3734	8.0996
1.36E-02	4.3819	3.8286	3.4815	2.0652	1.9894	8.3832	8.1042
1.92E-02	4.3919	3.8322	3.4831	2.0644	1.9971	8.3928	8.1078

Results page

```
 no. of spectra             15
 no. of resonance values    93
 no. of resonant nuclei      7

   sigma =   0.00208276193          RMS weighted residual =   0.00178095364

       stoich            value   relative    log    standard
       coeff                     std devn    beta   deviation
 Beta  1  1 refined  3.5677E+003  0.0963    3.5524   0.0418  (   GR   )
 Beta  1  2 refined  2.2306E+007  0.2059    7.3484   0.0894  (   GR2  )
 Beta  0  2 constant 4.4208E+002            2.6455           (   R2   )

 Individual chemical shifts
                  G                       R
 =========================================================
       +       value    error         value    error
CH-1   +      4.4214   0.0026
CH-6'  +      3.8422   0.0015
CH3    +      3.4880   0.0014
Ac'    +      2.0631   0.0013
Ac     +      2.0194   0.0022
CH-C   +                              7.9737   0.0781
CH-G   +                              6.7497   0.1138
       +
                 1,1                      1,2
 =========================================================
       +       value    error         value    error
CH-1   +      3.3006   0.0740         4.3181   0.0164
CH-6'  +      3.4590   0.0280         3.7986   0.0104
CH3    +      3.3064   0.0171         3.4678   0.0099
Ac'    +      2.1262   0.0130         2.0686   0.0098
Ac     +      1.1726   0.0543         1.8893   0.0340
CH-C   +      8.4080   0.0029         8.2519   0.0267
CH-G   +      8.1160   0.0023         8.2420   0.0203
       +
                 0,2
 =================================
       +       value    error
CH-1   +
CH-6'  +
CH3    +
Ac'    +
Ac     +
CH-C   +      7.8710   0.2208
CH-G   +      9.7161   0.3201

 Correlation coefficients*1000
      1    2
   1
   2  399

 Parameters are numbered as follows
    1 beta 1,1
    2 beta 1,2
```

Titration Plots

Chemical shifts (δ, ppm) *vs*. concentration of G (mol L^{-1})

experimental (symbols) and calculated (lines) values

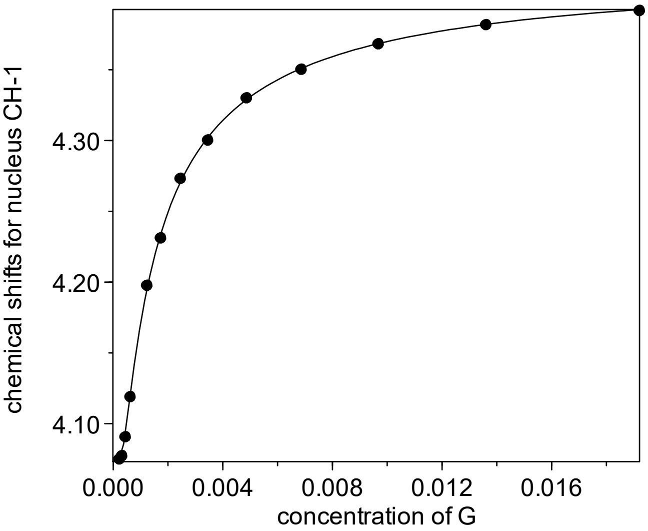

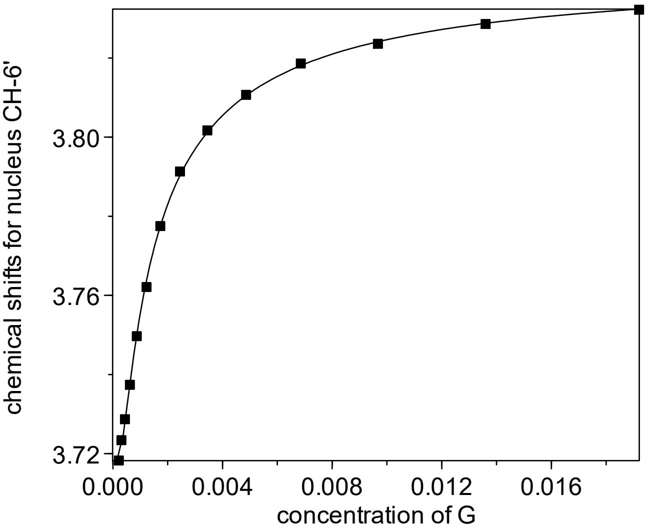

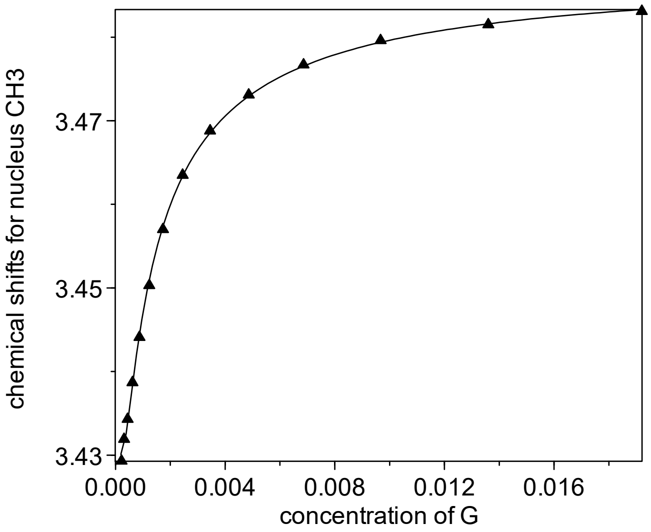

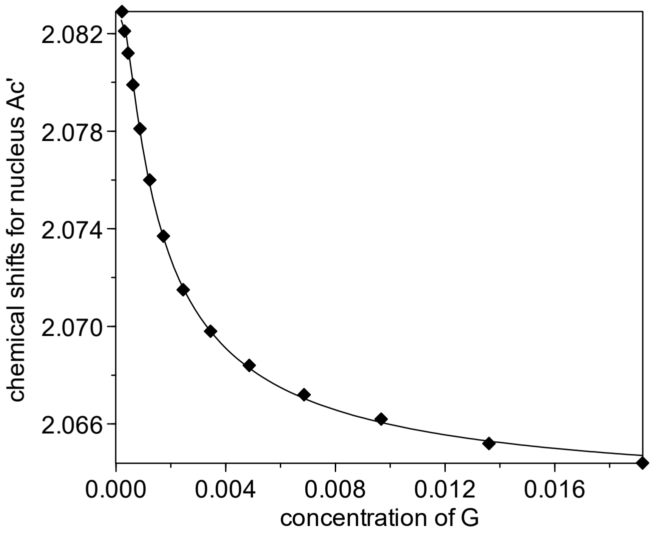

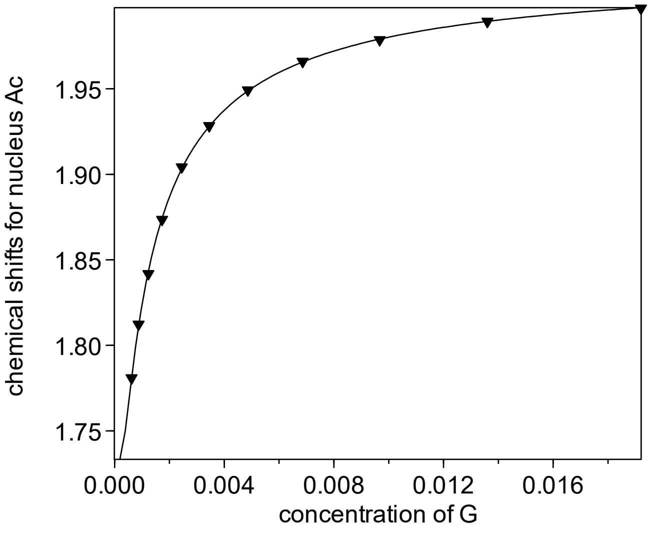

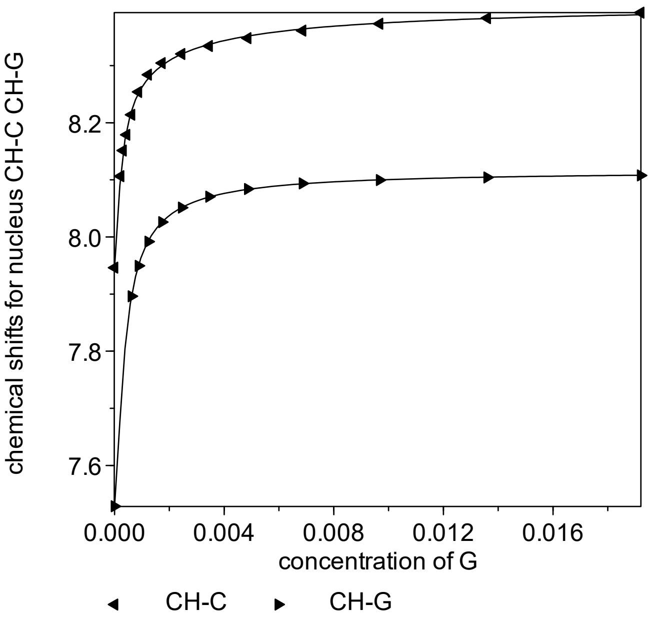
chemical shifts for nucleus CH-C CH-G
8.2
8.0
7.8
7.6
0.000
0.004
0.008
0.012
0.016
concentration of G
CH-C
CH-G

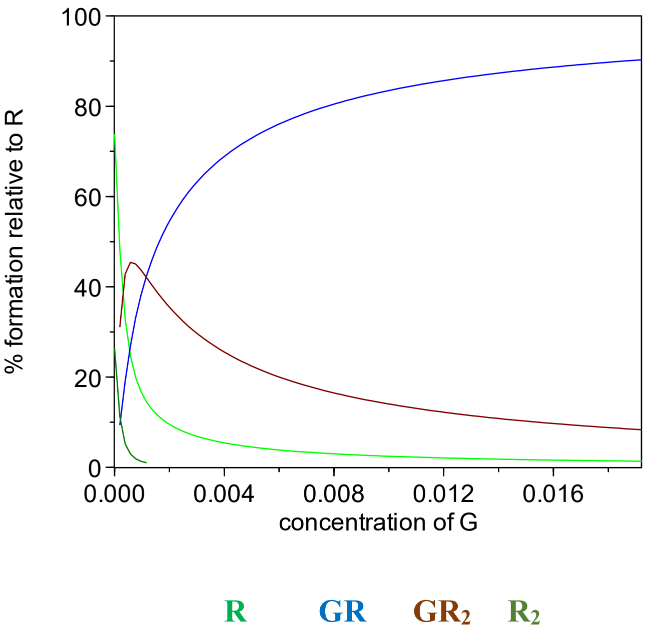
100
80
60
40
20
0
% formation relative to R
0.000
0.004
0.008
0.012
0.016
concentration of G
R
GR
GR2
R2

1 + MeβGlcNAc (D_2O, pD 7.4, 298 K, 500 MHz).

R = **1**   G = MeβGlcNAc

Titration

[R] = 4.97 10^{-4} mol L^{-}

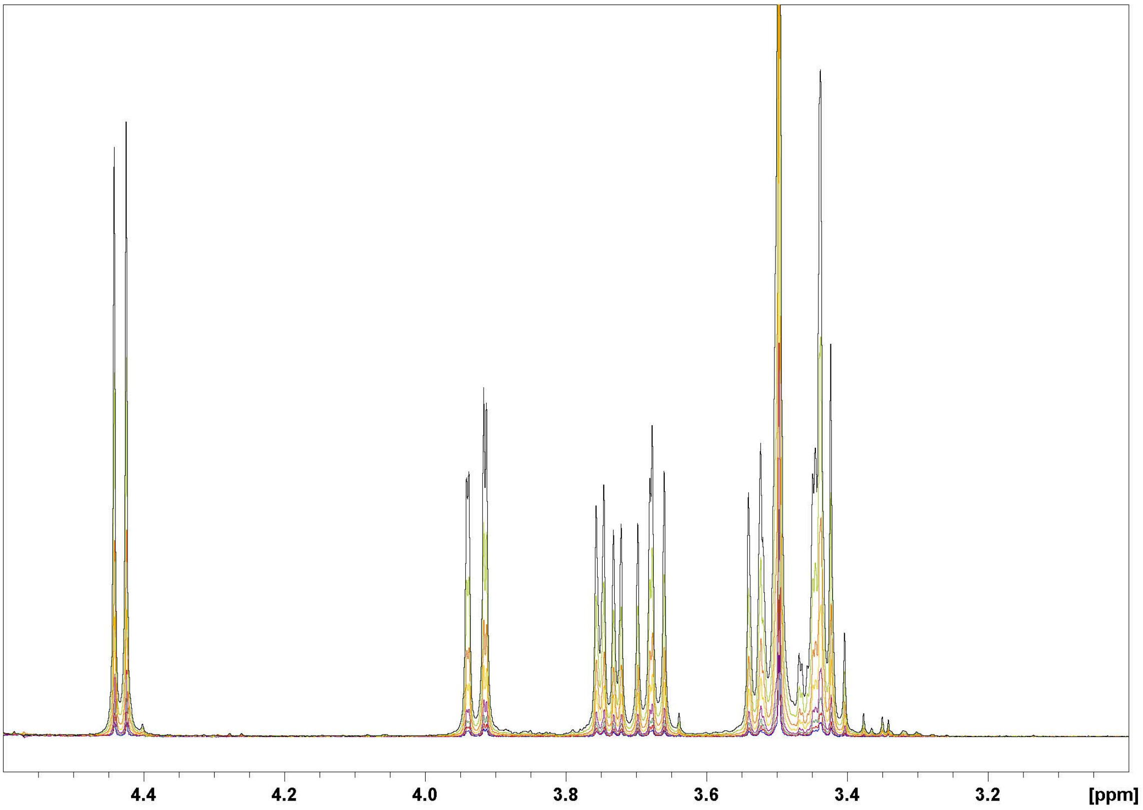

Figure S13. Superposition of ^{1}H NMR spectra registered at incremental concentrations of MeβGlcNAc (0.329 mM, 0.623 mM, 1.18 mM, 2.23 mM, 4.21 mM, 7.57 mM, 15.1 mM, 28.5 mM) in a 0.497 mM solution of **1**. Expansion of the saccharide region.

Table S1. Cumulative formation constants (log β_n)[a] and intrinsic median binding concentration (BC_{50}^0, mM)[b] for receptor **1** to MeβCeB (R:G) complexes, measured at 298 K from NMR data in D_2O at pD 11.[c]

R:G	log β_n	BC_{50}^0
1:1	2.50±0.08	1.39±0.29
2:1	6.46±0.10	

[a] Formation constants were obtained by nonlinear least-square regression analysis of NMR data. [b] Calculated from the log β values using the "BC50 Calculator" program.[15] [c] Receptor dimerization constant at pD 11 (**1**: log β_{dim} = 3.17±0.07) was set invariant in the nonlinear regression analysis of NMR data.

Calorimetric titrations and data analysis. Isothermal Titration Microcalorimetry experiments were performed at 298 K with a Nano-ITC instrument. After an initial injection of 3 μL, which was excluded from data analysis, aliquots of the titrant solution, containig the glycoside, were injected stepwise into the sample cell containing a solution of the titrate **1**. Titrate solutions containing **1** were prepared in H_2O adjusting the pH with a diluted NaOH solution. All experiments were performed in H_2O at pH 7.4. Heats of dilution were measured by injecting the titrant solution into neat H_2O and then subtracted from the binding heats. To remove ambiguities in the definition of binding models of receptor **1**, data from indipendent titrations performed at different concentrations of the titrate were simultaneously fitted to measure the cumulative association constants and the thermodynamic parameters using the HypCal software package.[S3] The dimerization constant log β_{dim} 2.65±0.07 of receptor **1** was set invariant in the non linear regression analysis of receptor-glycosides binding data.

1 + MeβCeB (H_2O, pH 7.4, 298 K).

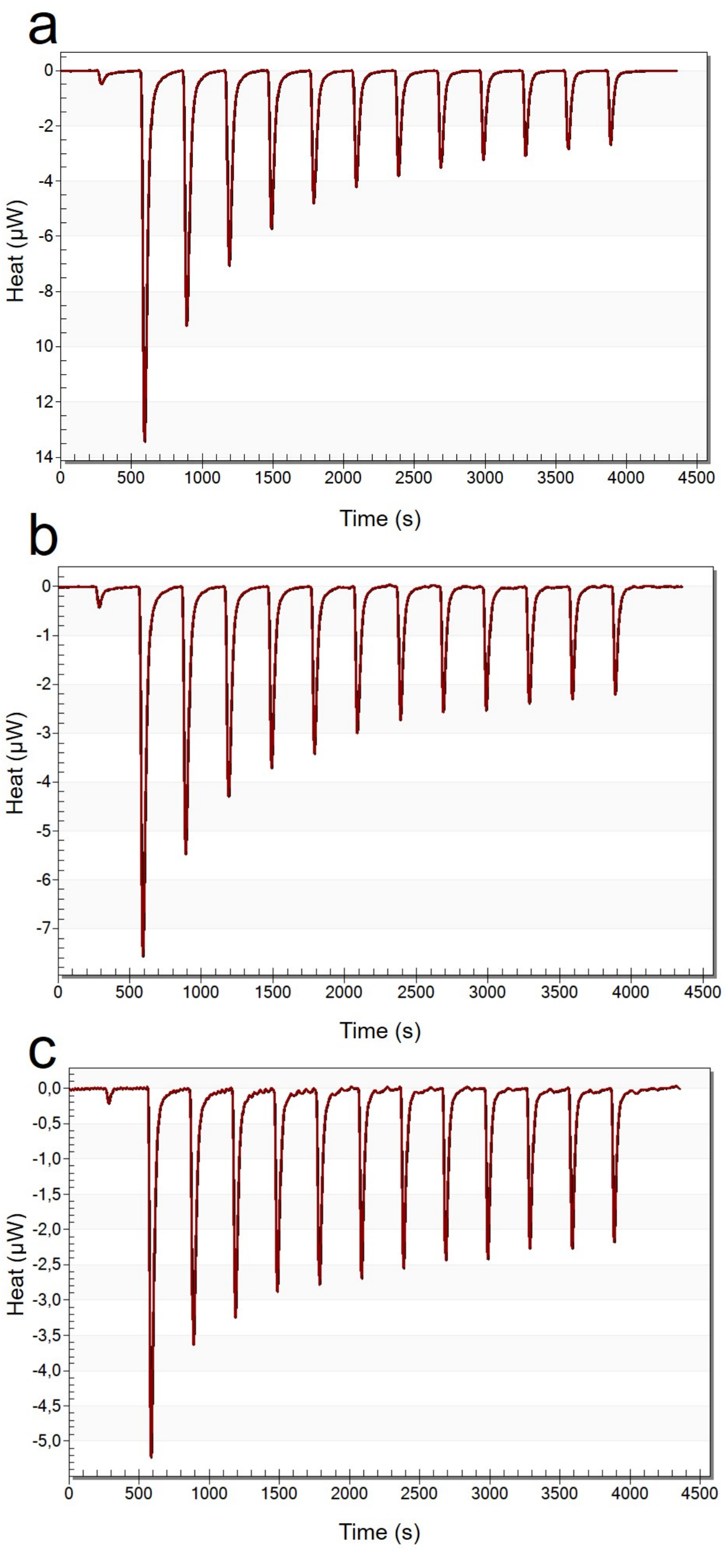

Figure S14. ITC results of MeβCeB with **1** in H_2O, pH 7.4, at 298 K: a) Titration of **1** ($1.25\ 10^{-4}$ mol L^{-1}) with MeβCeB ($4.04\ 10^{-2}$ mol L^{-1}); b) Titration of **1** ($6.25\ 10^{-5}$ mol L^{-1}) with MeβCeB ($4.04\ 10^{-2}$ mol L^{-1}); c) Titration of **1** ($3.13\ 10^{-5}$ mol L^{-1}) with MeβCeB ($4.04\ 10^{-2}$ mol L^{-1}).

Data Table

R = **1** G = MeβCeB

[G] = 4.04 10^{-2} mol L^{-1}

Titration 1: [R] = 1.25 10^{-4} mol L^{-1}

Injection	Q (μJ)	Corrected Q (μJ)	inj volume (μL)	mol G (mol)	mol R (mol)	mol G / mol R	total volume (μL)
1	-30.9766	-27.6163	3	1.21E-07	1.18E-07	1.029299	945
2	-582.334	-489.594	20	9.27E-07	1.15E-07	8.039663	945
3	-400.243	-314.737	20	1.72E-06	1.13E-07	15.2016	945
4	-299.109	-218.062	20	2.49E-06	1.10E-07	22.51839	945
5	-241.943	-162.067	20	3.24E-06	1.08E-07	29.99338	945
6	-199.557	-125.591	20	3.98E-06	1.06E-07	37.63	945
7	-171.782	-99.1094	20	4.71E-06	1.04E-07	45.43173	945
8	-153.049	-78.1583	20	5.41E-06	1.01E-07	53.40214	945
9	-135.395	-65.943	20	6.11E-06	9.92E-08	61.54489	945
10	-122.045	-50.2804	20	6.79E-06	9.71E-08	69.8637	945
11	-114.121	-45.7163	20	7.45E-06	9.51E-08	78.36238	945
12	-103.462	-35.4923	20	8.10E-06	9.31E-08	87.04481	945
13	-101.806	-36.1191	20	8.74E-06	9.11E-08	95.91496	945

Titration 2: [R] = 6.25 10^{-5} mol L^{-1}

Injection	Q (μJ)	Corrected Q (μJ)	inj volume (μL)	mol G (mol)	mol R (mol)	mol G / mol R	total volume (μL)
1	-21.4848	-18.1244	3	1.21E-07	5.89E-08	2.058599	945
2	-328.267	-235.527	20	9.27E-07	5.76E-08	16.07933	945
3	-231.113	-145.607	20	1.72E-06	5.64E-08	30.4032	945
4	-180.018	-98.9708	20	2.49E-06	5.52E-08	45.03679	945
5	-151.376	-71.4999	20	3.24E-06	5.40E-08	59.98677	945
6	-135.309	-61.343	20	3.98E-06	5.29E-08	75.26	945
7	-112.061	-39.3888	20	4.71E-06	5.18E-08	90.86346	945
8	-100.561	-25.6711	20	5.41E-06	5.07E-08	106.8043	945
9	-92.6911	-23.2389	20	6.11E-06	4.96E-08	123.0898	945
10	-94.6884	-22.9239	20	6.79E-06	4.86E-08	139.7274	945
11	-86.4853	-18.0804	20	7.45E-06	4.75E-08	156.7248	945
12	-80.3039	-12.3341	20	8.10E-06	4.65E-08	174.0896	945
13	-76.5588	-10.8719	20	8.74E-06	4.55E-08	191.8299	945

Titration 3: [R] = 3.13 10^{-5} mol L^{-1}

Injection	Q (μJ)	Corrected Q (μJ)	inj volume (μL)	mol G (mol)	mol R (mol)	mol G / mol R	total volume (μL)
1	-5.956317667	-2.596000333	3	1.21E-07	2.94E-08	4.123795525	945
2	-201.274375	-108.534725	20	9.27E-07	2.88E-08	32.21018667	945
3	-152.692475	-67.186525	20	1.72E-06	2.82E-08	60.90385113	945
4	-131.606875	-50.559875	20	2.49E-06	2.76E-08	90.21791916	945
5	-114.939075	-35.062675	20	3.24E-06	2.70E-08	120.1658049	945
6	-103.531325	-29.565625	20	3.98E-06	2.64E-08	150.7612124	945
7	-94.42565	-21.752975	20	4.71E-06	2.59E-08	182.0181423	945
8	-89.067975	-14.1778	20	5.41E-06	2.53E-08	213.9508977	945
9	-81.4659	-12.013775	20	6.11E-06	2.48E-08	246.5740911	945
10	-83.05675	-11.292275	20	6.79E-06	2.42E-08	279.9026508	945
11	-81.4706	-13.0657	20	7.45E-06	2.37E-08	313.951828	945
12	-80.8955	-12.925675	20	8.10E-06	2.32E-08	348.7372036	945
13	-77.3666	-11.6797	20	8.74E-06	2.27E-08	384.2746955	945

Results page

```
Reagent Reagent
  number   name
    1      R
    2      G

   sigma =   0.00363

 Formation constants   Value    relative    log    standard
                                std devn    beta   deviation
 Beta A refined     0.3790E+03  0.0722    2.5787    0.0314    1  1
 Beta B refined     0.1899E+06  0.6580    5.2786    0.2857    2  1
 Beta C constant    0.4421E+03            2.6455    0.0676    2  0


 Formation entalpies    Value   standard
                                deviation
-DeltaH A refined      15.5995   0.6640
-DeltaH B refined    -109.3421  46.6052
-DeltaH C refined    -108.9897  20.9161


+++++++++++++++++++++++++++++++++++++++++++++++++++++++++++++++++++++++++++++++++
 Thermodynamic Functions, kJ/mol


              - DeltaG°                - DeltaH°                 T DeltaS°
   A       14.7191    0.1790       15.5995    0.6640         -0.8803    0.8176
   B       30.1304    1.6311     -109.3421   46.6052        139.4725   47.5953
   C       15.1006    0.1055     -108.9897   20.9161        124.0903   20.9163


+++++++++++++++++++++++++++++++++++++++++++++++++++++++++++++++++++++++++++++++++


 Correlation coefficients*1000         Run timed at 14.31 on  4 Sep 2020

   2  588
   3 -823 -432
   4  260 -596 -380
   5  812  711 -910   69
       1    2    3    4

 Order of parameters:
   1       Beta   A
   2       Beta   B
   3      -DeltaH A
   4      -DeltaH B
   5      -DeltaH C
```

Results table

Addition (µL)	Qobs (mJ)	Qcalc (mJ)	residual (mJ)	QTobs (mJ)	QTcalc (mJ)
1.1300	-0.0276				
21.1300	-0.4896	-0.4898	2.0000e-4	-0.5172	-0.5174
41.1300	-0.3147	-0.3163	1.6000e-3	-0.8319	-0.8337
61.1300	-0.2181	-0.2200	1.9000e-3	-1.0500	-1.0537
81.1300	-0.1621	-0.1613	-8.0000e-4	-1.2121	-1.2150
101.1300	-0.1256	-0.1230	-2.6000e-3	-1.3377	-1.3380
121.1300	-0.0991	-0.0967	-2.4000e-3	-1.4368	-1.4347
141.1300	-0.0782	-0.0779	-3.0000e-4	-1.5149	-1.5126
161.1300	-0.0659	-0.0640	-2.0000e-3	-1.5809	-1.5765
181.1300	-0.0503	-0.0534	3.1000e-3	-1.6312	-1.6300
201.1300	-0.0457	-0.0452	-5.0000e-4	-1.6769	-1.6752
221.1300	-0.0355	-0.0387	3.2000e-3	-1.7124	-1.7139
241.1300	-0.0361	-0.0335	-2.6000e-3	-1.7485	-1.7474
1.5400	-0.0181				
21.5400	-0.2355	-0.2308	-4.8000e-3	-0.2537	-0.2489
41.5400	-0.1456	-0.1447	-9.0000e-4	-0.3993	-0.3936
61.5400	-0.0990	-0.0989	-1.0000e-4	-0.4982	-0.4925
81.5400	-0.0715	-0.0717	2.0000e-4	-0.5697	-0.5642
101.5400	-0.0613	-0.0543	-7.0000e-3	-0.6311	-0.6185
121.5400	-0.0394	-0.0425	3.1000e-3	-0.6705	-0.6610
141.5400	-0.0257	-0.0341	8.4000e-3	-0.6961	-0.6951
161.5400	-0.0232	-0.0280	4.7000e-3	-0.7194	-0.7231
181.5400	-0.0229	-0.0233	4.0000e-4	-0.7423	-0.7464
201.5400	-0.0181	-0.0197	1.6000e-3	-0.7604	-0.7661
221.5400	-0.0123	-0.0169	4.5000e-3	-0.7727	-0.7830
241.5400	-0.0109	-0.0146	3.7000e-3	-0.7836	-0.7976
0.4800	-2.6000e-3				
20.4800	-0.1085	-0.1146	6.0000e-3	-0.1111	-0.1172
40.4800	-0.0672	-0.0702	3.0000e-3	-0.1783	-0.1874
60.4800	-0.0506	-0.0473	-3.2000e-3	-0.2289	-0.2347
80.4800	-0.0351	-0.0340	-1.0000e-3	-0.2639	-0.2687
100.4800	-0.0296	-0.0256	-4.0000e-3	-0.2935	-0.2943
120.4800	-0.0218	-0.0200	-1.8000e-3	-0.3153	-0.3143
140.4800	-0.0142	-0.0160	1.8000e-3	-0.3294	-0.3302
160.4800	-0.0120	-0.0131	1.1000e-3	-0.3414	-0.3433
180.4800	-0.0113	-0.0109	-4.0000e-4	-0.3527	-0.3542
200.4800	-0.0131	-9.2000e-3	-3.9000e-3	-0.3658	-0.3634
220.4800	-0.0129	-7.9000e-3	-5.1000e-3	-0.3787	-0.3713
240.4800	-0.0117	-6.8000e-3	-4.9000e-3	-0.3904	-0.3781

Titration Plots

Experimental (symbols) and calculated (cross and lines) heats

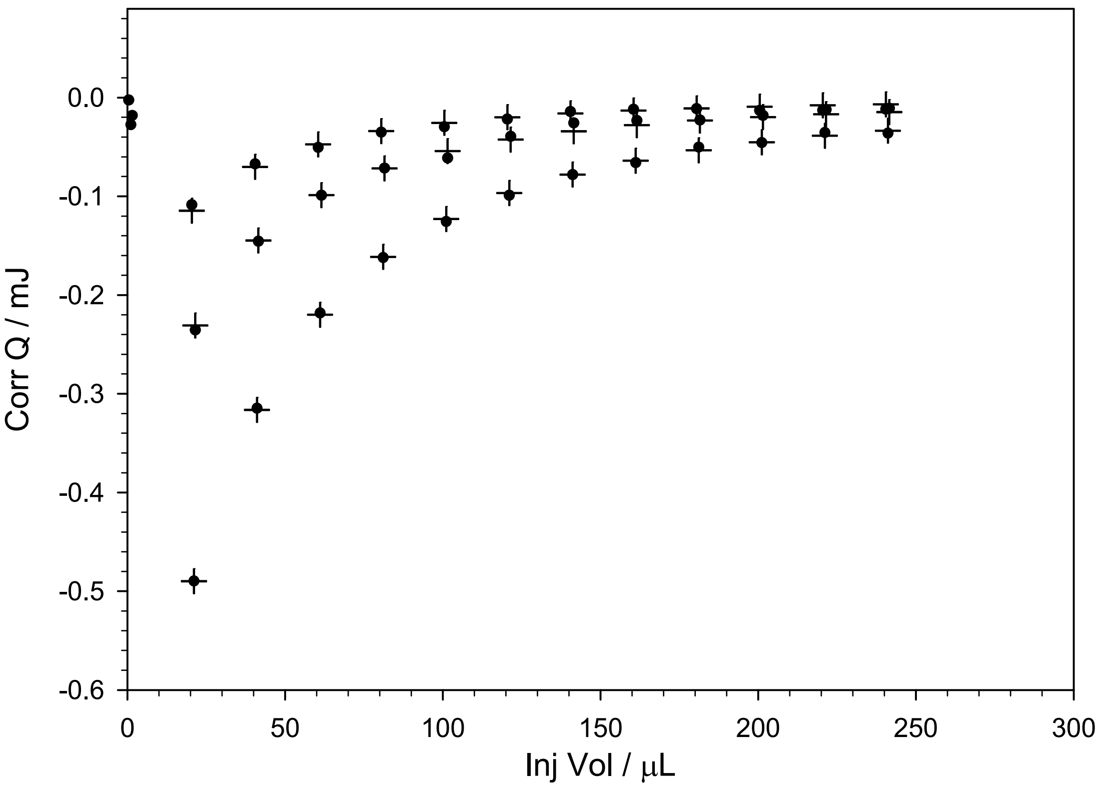

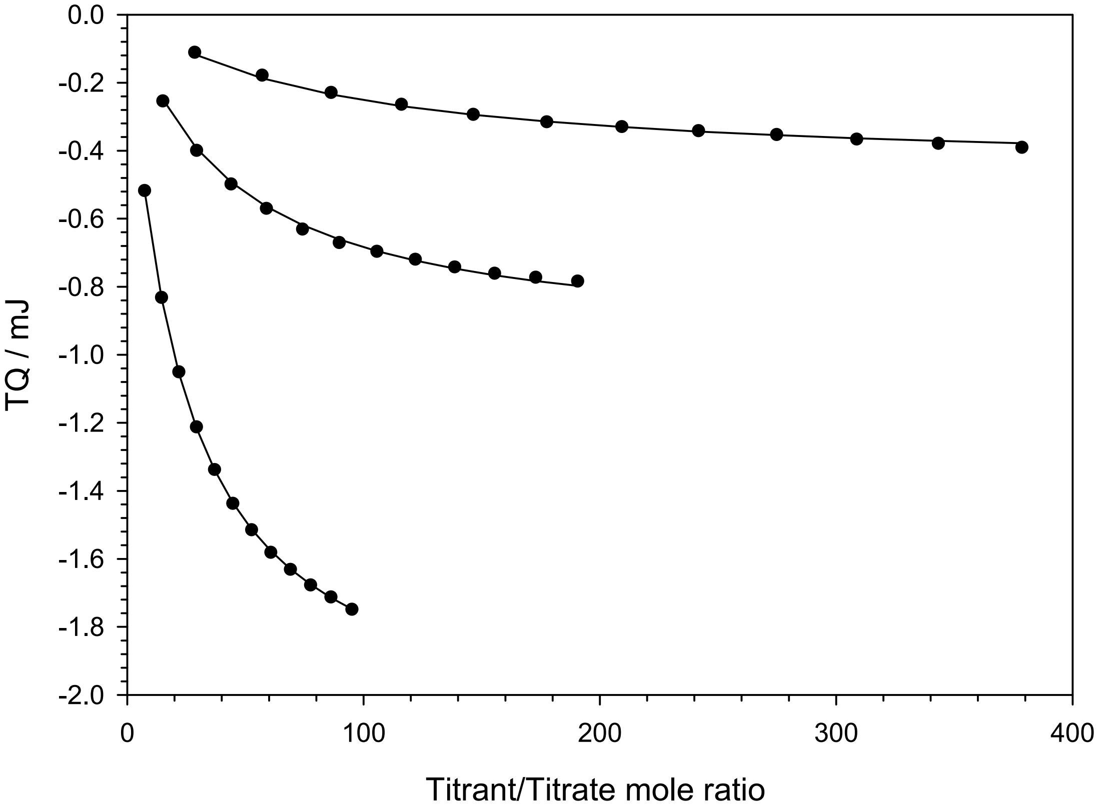

1 + MeβMal (H_2O, pH 7.4, 298 K).

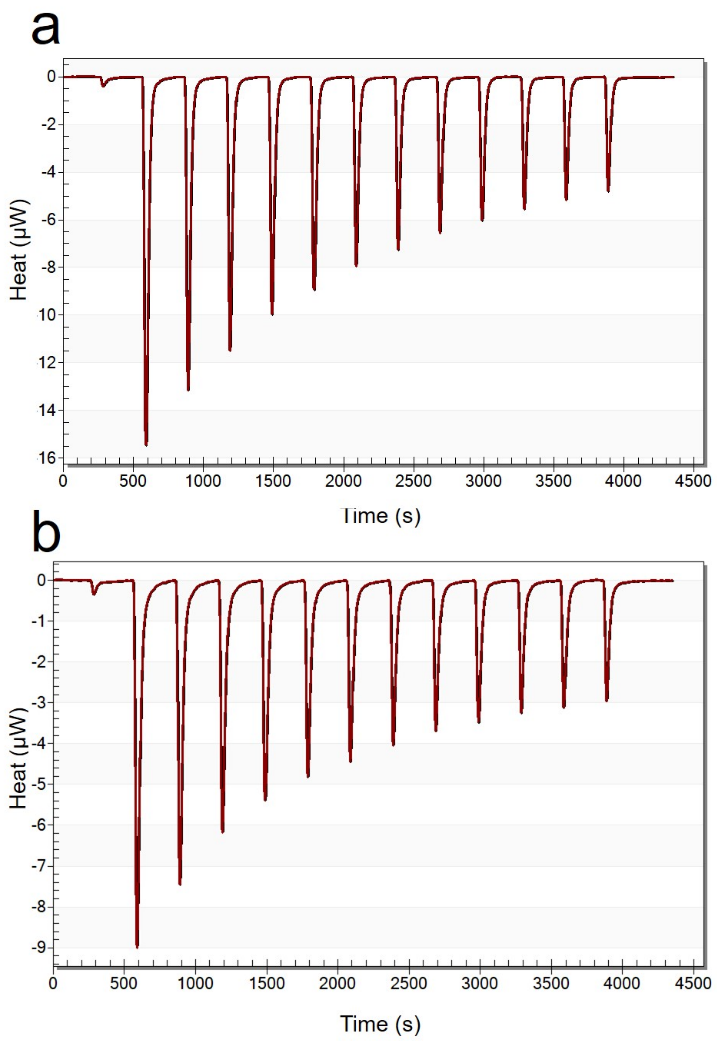

Figure S15. ITC results of MeβMal with **1** in H_2O, pH 7.4, at 298 K: a) Titration of **1** (2.50 10^{-4} mol L^{-1}) with MeβMal (4.04 10^{-2} mol L^{-1}); b) Titration of **1** (1.25 10^{-4} mol L^{-1}) with MeβMal (4.04 10^{-2} mol L^{-1})

Data Table

R = **1**   G = MeβMal

[G] = 4.04 10^{-2} mol L^{-1}

Titration 1: [R] = 2.50 10^{-4} mol L^{-1}

Injection	Q (μJ)	Corrected Q (μJ)	inj volume (μL)	mol G (mol)	mol R (mol)	mol G / mol R	total volume (μL)
1	-21.102	-11.5977	3	1.21E-07	2.36E-07	0.51465	945
2	-550.989	-443.994	20	9.27E-07	2.31E-07	4.019831	945
3	-470.059	-372.192	20	1.72E-06	2.26E-07	7.600801	945
4	-403.38	-308.473	20	2.49E-06	2.21E-07	11.2592	945
5	-349.554	-258.811	20	3.24E-06	2.16E-07	14.99669	945
6	-309.931	-223.443	20	3.98E-06	2.12E-07	18.815	945
7	-276.198	-192.986	20	4.71E-06	2.07E-07	22.71586	945
8	-250.262	-174.693	20	5.41E-06	2.03E-07	26.70107	945
9	-225.74	-143.412	20	6.11E-06	1.98E-07	30.77245	945
10	-199.48	-122.548	20	6.79E-06	1.94E-07	34.93185	945
11	-188.203	-113.932	20	7.45E-06	1.90E-07	39.18119	945
12	-172.74	-99.8081	20	8.10E-06	1.86E-07	43.5224	945
13	-164.805	-85.9617	20	8.74E-06	1.82E-07	47.95748	945

Titration 2: [R] = 1.25 10^{-4} mol L^{-1}

Injection	Q (μJ)	Corrected Q (μJ)	inj volume (μL)	mol G (mol)	mol R (mol)	mol G / mol R	total volume (μL)
1	-18.383	-8.87876	3	1.21E-07	1.18E-07	1.029299	945
2	-371.732	-264.736	20	9.27E-07	1.15E-07	8.039663	945
3	-309.987	-212.12	20	1.72E-06	1.13E-07	15.2016	945
4	-256.678	-161.771	20	2.49E-06	1.10E-07	22.51839	945
5	-222.233	-131.491	20	3.24E-06	1.08E-07	29.99338	945
6	-198.057	-111.569	20	3.98E-06	1.06E-07	37.63	945
7	-179.709	-96.4967	20	4.71E-06	1.04E-07	45.43173	945
8	-159.616	-84.048	20	5.41E-06	1.01E-07	53.40214	945
9	-146.976	-64.6484	20	6.11E-06	9.92E-08	61.54489	945
10	-130.566	-53.6335	20	6.79E-06	9.71E-08	69.8637	945
11	-125.921	-51.6502	20	7.45E-06	9.51E-08	78.36238	945
12	-113.831	-40.8998	20	8.10E-06	9.31E-08	87.04481	945
13	-113.2	-34.357	20	8.74E-06	9.11E-08	95.91496	945

Results page

```
Reagent Reagent
  number   name
    1      R
    2      G

   sigma =   0.00596

 Formation constants   Value    relative    log    standard
                                std devn    beta   deviation
 Beta A refined     0.1756E+03  0.0309    2.2444    0.0134    1  1
 Beta B constant    0.4421E+03            2.6455    0.0676    2  0


 Formation entalpies    Value   standard
                                deviation
-DeltaH A refined      20.7240   0.4795
-DeltaH B refined      23.7075   6.1583


++++++++++++++++++++++++++++++++++++++++++++++++++++++++++++++++++++++++++++++++
 Thermodynamic Functions, kJ/mol


              - DeltaG°                - DeltaH°                 T DeltaS°
   A        12.8113    0.0766        20.7240    0.4795        -7.9127    0.4721
   B        15.1006    0.0574        23.7075    6.1583        -8.6070    6.1585


++++++++++++++++++++++++++++++++++++++++++++++++++++++++++++++++++++++++++++++++


 Correlation coefficients*1000         Run timed at 13.42 on  1 Sep 2020

   2  175
   3  670  825
       1    2

 Order of parameters:
   1       Beta   A
   2      -DeltaH A
   3      -DeltaH B
```

Results table

Addition (µL)	Qobs (mJ)	Qcalc (mJ)	residual (mJ)	QTobs (mJ)	QTcalc (mJ)
0.5200	-0.0116				
20.5200	-0.4440	-0.4507	6.7000e-3	-0.4556	-0.4623
40.5200	-0.3722	-0.3708	-1.4000e-3	-0.8278	-0.8331
60.5200	-0.3085	-0.3082	-2.0000e-4	-1.1363	-1.1413
80.5200	-0.2588	-0.2590	2.0000e-4	-1.3951	-1.4003
100.5200	-0.2234	-0.2198	-3.7000e-3	-1.6185	-1.6201
120.5200	-0.1930	-0.1883	-4.7000e-3	-1.8115	-1.8084
140.5200	-0.1747	-0.1628	-0.0119	-1.9862	-1.9712
160.5200	-0.1434	-0.1418	-1.6000e-3	-2.1296	-2.1130
180.5200	-0.1225	-0.1245	2.0000e-3	-2.2522	-2.2375
200.5200	-0.1139	-0.1100	-3.9000e-3	-2.3661	-2.3475
220.5200	-0.0998	-0.0979	-1.9000e-3	-2.4659	-2.4454
240.5200	-0.0860	-0.0875	1.6000e-3	-2.5519	-2.5329
0.6700	-8.9000e-3				
20.6700	-0.2647	-0.2585	-6.2000e-3	-0.2736	-0.2674
40.6700	-0.2121	-0.2059	-6.2000e-3	-0.4857	-0.4733
60.6700	-0.1618	-0.1671	5.3000e-3	-0.6475	-0.6404
80.6700	-0.1315	-0.1378	6.3000e-3	-0.7790	-0.7782
100.6700	-0.1116	-0.1153	3.7000e-3	-0.8906	-0.8934
120.6700	-0.0965	-0.0977	1.2000e-3	-0.9871	-0.9911
140.6700	-0.0840	-0.0837	-4.0000e-4	-1.0711	-1.0748
160.6700	-0.0646	-0.0724	7.8000e-3	-1.1358	-1.1472
180.6700	-0.0536	-0.0632	9.6000e-3	-1.1894	-1.2105
200.6700	-0.0517	-0.0557	4.0000e-3	-1.2410	-1.2662
220.6700	-0.0409	-0.0493	8.4000e-3	-1.2819	-1.3155
240.6700	-0.0344	-0.0440	9.6000e-3	-1.3163	-1.3595

Titration Plots

Experimental (symbols) and calculated (cross and lines) heats

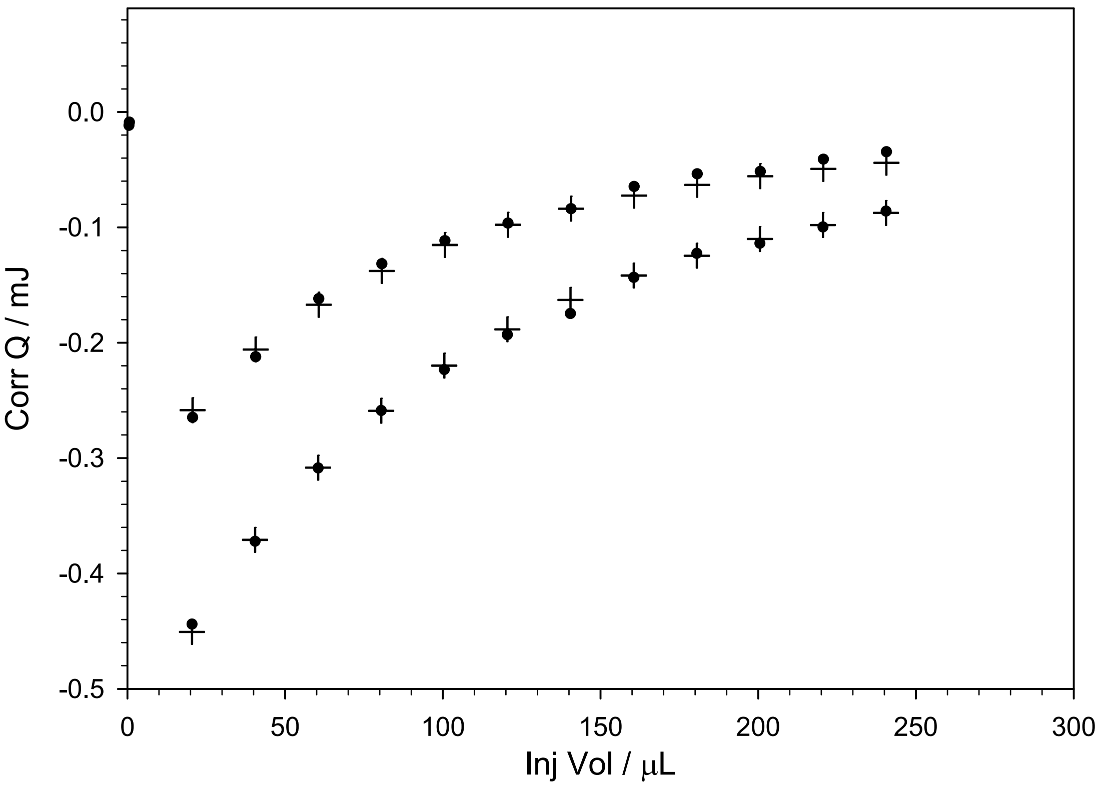

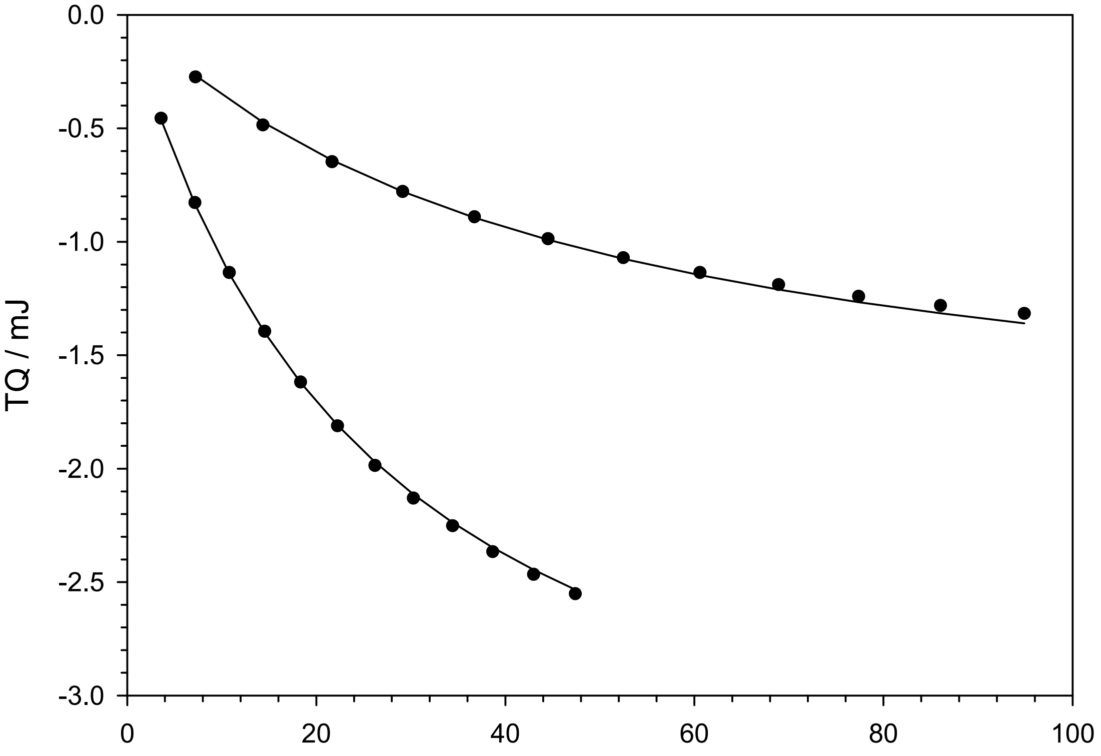

1 + MeβLac (H_2O, pH 7.4, 298 K).

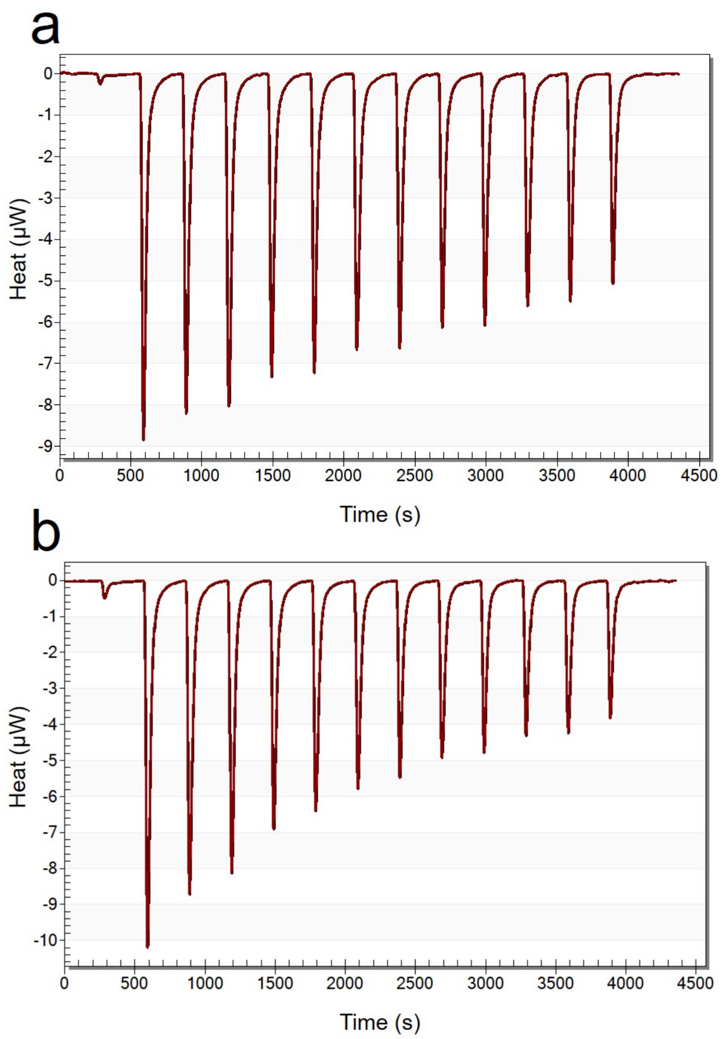

Figure S16. ITC results of MeβLac with **1** in H_2O, pH 7.4, at 298 K: a) Titration of **1** ($5.00\ 10^{-4}$ mol L^{-1}) with MeβLac ($4.05\ 10^{-2}$ mol L^{-1}); b) Titration of **1** ($2.50\ 10^{-4}$ mol L^{-1}) with MeβLac ($4.05\ 10^{-2}$ mol L^{-1})

Data Table

R = **1** G = MeβLac

[G] = 4.05 10^{-2} mol L^{-1}

Titration 1: [R] = 5.00 10^{-4} mol L^{-1}

Injection	Q (μJ)	Corrected Q (μJ)	inj volume (μL)	mol G (mol)	mol R (mol)	mol G / mol R	total volume (μL)
1	-12.6643	-10.3446	3	1.22E-07	4.71E-07	0.257962	945
2	-355.837	-255.389	20	9.29E-07	4.61E-07	2.014891	945
3	-337.515	-246.202	20	1.72E-06	4.51E-07	3.809807	945
4	-321.21	-231.573	20	2.49E-06	4.42E-07	5.643533	945
5	-307.439	-220.479	20	3.25E-06	4.32E-07	7.516906	945
6	-294.123	-207.986	20	3.99E-06	4.23E-07	9.430786	945
7	-278.541	-197.392	20	4.72E-06	4.14E-07	11.38605	945
8	-269.484	-193.411	20	5.43E-06	4.05E-07	13.38358	945
9	-254.601	-175.159	20	6.12E-06	3.97E-07	15.42431	945
10	-242.224	-165.482	20	6.80E-06	3.89E-07	17.50916	945
11	-229.656	-152.557	20	7.47E-06	3.80E-07	19.63909	945
12	-211.43	-136.949	20	8.12E-06	3.72E-07	21.81507	945
13	-203.921	-128.65	20	8.76E-06	3.64E-07	24.03809	945

Titration 2: [R] = 2.50 10^{-4} mol L^{-1}

Injection	Q (μJ)	Corrected Q (μJ)	inj volume (μL)	mol G (mol)	mol R (mol)	mol G / mol R	total volume (μL)
1	-25.6331	-23.3134	3	1.22E-07	2.36E-07	0.515924	945
2	-428.45	-328.001	20	9.29E-07	2.31E-07	4.029781	945
3	-378.526	-287.214	20	1.72E-06	2.26E-07	7.619614	945
4	-337.159	-247.522	20	2.49E-06	2.21E-07	11.28707	945
5	-301.294	-214.334	20	3.25E-06	2.16E-07	15.03381	945
6	-270.68	-184.543	20	3.99E-06	2.12E-07	18.86157	945
7	-250.135	-168.986	20	4.72E-06	2.07E-07	22.77209	945
8	-226.28	-150.207	20	5.43E-06	2.03E-07	26.76716	945
9	-209.695	-130.252	20	6.12E-06	1.98E-07	30.84862	945
10	-190.567	-113.825	20	6.80E-06	1.94E-07	35.01832	945
11	-174.747	-97.6488	20	7.47E-06	1.90E-07	39.27817	945
12	-166.944	-92.464	20	8.12E-06	1.86E-07	43.63013	945
13	-159.098	-83.8265	20	8.76E-06	1.82E-07	48.07619	945

Results page

```
Reagent Reagent
  number   name
    1      R
    2      G

   sigma =   0.00992

 Formation constants   Value    relative    log    standard
                                std devn    beta   deviation
 Beta A refined     0.2043E+03  0.0517    2.3103    0.0225    1  1
 Beta B constant    0.4421E+03            2.6455    0.0676    2  0


 Formation entalpies    Value   standard
                                deviation
-DeltaH A refined      23.8721   0.6050
-DeltaH B refined     101.0108   2.8812


++++++++++++++++++++++++++++++++++++++++++++++++++++++++++++++++++++++++++++++++
 Thermodynamic Functions, kJ/mol


               - DeltaG°                - DeltaH°                 T DeltaS°
   A        13.1871    0.1283         23.8721    0.6050        -10.6850    0.6996
   B        15.1006    0.0628        101.0108    2.8812        -85.9102    2.8819


++++++++++++++++++++++++++++++++++++++++++++++++++++++++++++++++++++++++++++++++


 Correlation coefficients*1000         Run timed at 16.38 on 28 Aug 2020

   2 -689
   3 -261  862
       1    2

 Order of parameters:
   1       Beta   A
   2      -DeltaH A
   3      -DeltaH B
```

Results table

Addition (µL)	Qobs (mJ)	Qcalc (mJ)	residual (mJ)	QTobs (mJ)	QTcalc (mJ)
0.8100	-0.0103				
20.8100	-0.2554	-0.2342	-0.0212	-0.2657	-0.2445
40.8100	-0.2462	-0.2478	1.6000e-3	-0.5119	-0.4923
60.8100	-0.2316	-0.2474	0.0158	-0.7435	-0.7397
80.8100	-0.2205	-0.2387	0.0183	-0.9640	-0.9784
100.8100	-0.2080	-0.2258	0.0178	-1.1720	-1.2042
120.8100	-0.1974	-0.2108	0.0135	-1.3694	-1.4150
140.8100	-0.1934	-0.1955	2.0000e-3	-1.5628	-1.6105
160.8100	-0.1752	-0.1804	5.3000e-3	-1.7379	-1.7909
180.8100	-0.1655	-0.1662	7.0000e-4	-1.9034	-1.9571
200.8100	-0.1526	-0.1530	4.0000e-4	-2.0560	-2.1101
220.8100	-0.1369	-0.1409	3.9000e-3	-2.1929	-2.2509
240.8100	-0.1287	-0.1298	1.1000e-3	-2.3216	-2.3807
1.4200	-0.0233				
21.4200	-0.3280	-0.3169	-0.0111	-0.3513	-0.3402
41.4200	-0.2872	-0.2776	-9.7000e-3	-0.6385	-0.6178
61.4200	-0.2475	-0.2416	-5.9000e-3	-0.8861	-0.8594
81.4200	-0.2143	-0.2102	-4.1000e-3	-1.1004	-1.0696
101.4200	-0.1845	-0.1833	-1.2000e-3	-1.2849	-1.2529
121.4200	-0.1690	-0.1605	-8.5000e-3	-1.4539	-1.4134
141.4200	-0.1502	-0.1412	-9.0000e-3	-1.6041	-1.5546
161.4200	-0.1303	-0.1249	-5.4000e-3	-1.7344	-1.6795
181.4200	-0.1138	-0.1110	-2.8000e-3	-1.8482	-1.7905
201.4200	-0.0976	-0.0992	1.5000e-3	-1.9458	-1.8897
221.4200	-0.0925	-0.0890	-3.4000e-3	-2.0383	-1.9788
241.4200	-0.0838	-0.0803	-3.5000e-3	-2.1221	-2.0590

Titration Plots

Experimental (symbols) and calculated (cross and lines) heats

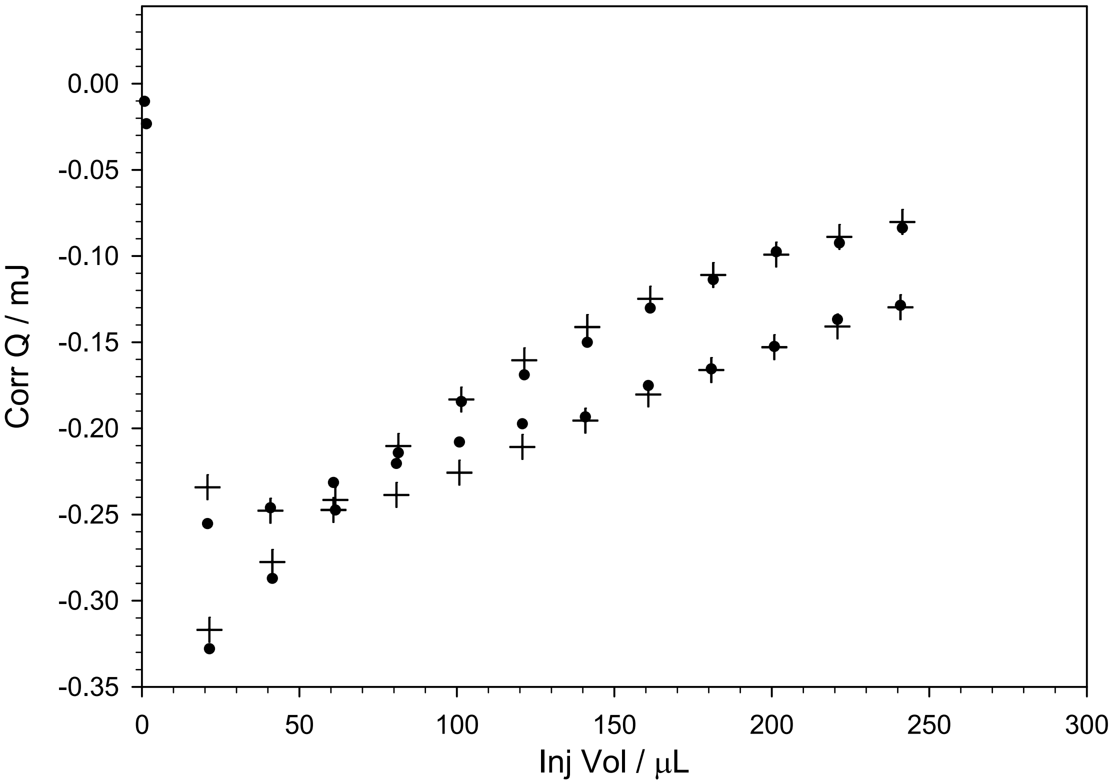

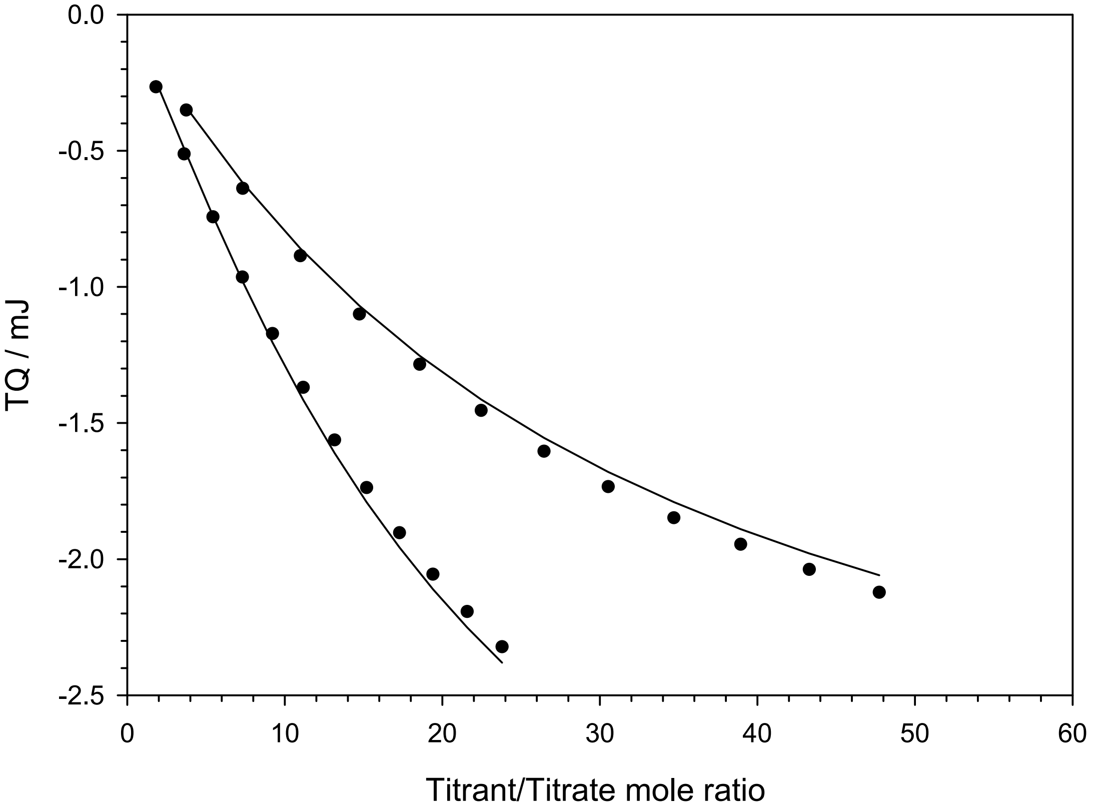

1 + MeβGlcNAc2 (H_2O, pH 7.4, 298 K).

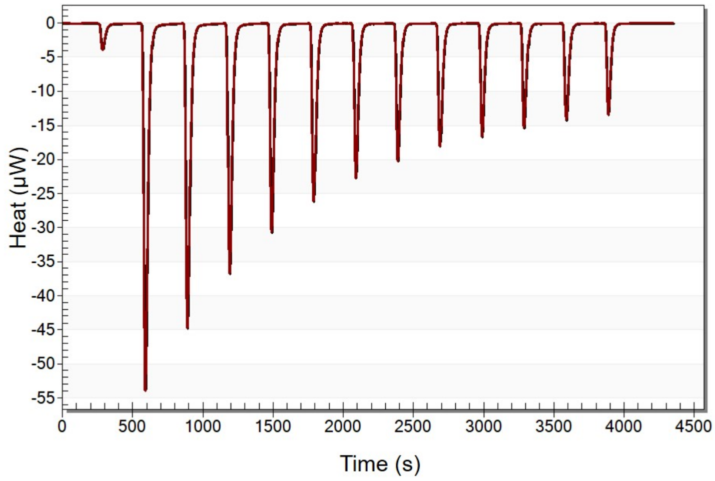

Figure S17. ITC results the titration of **1** ($4.04\ 10^{-4}$ mol L^{-1}) with MeβGlcNAc2 ($1.06\ 10^{-2}$ mol L^{-1}) in H_2O, pH 7.4, at 298 K

Data Table

R = **1** G = MeβGlcNAc2

[G] = $1.06\ 10^{-2}$ mol L^{-1}

Titration: [R] = $4.04\ 10^{-4}$ mol L^{-1}

Injection	Q (µJ)	Corrected Q (µJ)	inj volume (µL)	mol G (mol)	mol R (mol)	mol G / mol R	total volume (µL)
1	-141.752	-126.83	3	3.18E-08	4.18E-07	0.076031	945
2	-1898.54	-1673.16	20	2.43E-07	4.09E-07	0.593867	945
3	-1590.97	-1423.57	20	4.50E-07	4.01E-07	1.122899	945
4	-1294.78	-1286.4	20	6.52E-07	3.92E-07	1.66337	945
5	-1073.32	-1126.42	20	8.51E-07	3.84E-07	2.215527	945
6	-906.664	-957.795	20	1.04E-06	3.76E-07	2.779622	945
7	-781.13	-821.685	20	1.23E-06	3.68E-07	3.355914	945
8	-688.656	-716.257	20	1.42E-06	3.60E-07	3.944666	945
9	-617.208	-636.098	20	1.60E-06	3.52E-07	4.546148	945
10	-564.001	-584.506	20	1.78E-06	3.45E-07	5.160635	945
11	-515.716	-533.281	20	1.95E-06	3.38E-07	5.788408	945
12	-476.9	-488.287	20	2.13E-06	3.31E-07	6.429755	945
13	-435.708	-434.053	20	2.29E-06	3.24E-07	7.084968	945

Results page

```
Reagent Reagent
  number   name
    1      R
    2      G

   sigma =   0.01205

 Formation constants   Value    relative    log    standard
                                std devn    beta   deviation
 Beta A refined     0.3066E+04  0.1560    3.4865    0.0677    1  1
 Beta B refined     0.5192E+08  0.5175    7.7153    0.2247    2  1
 Beta C constant    0.4421E+03            2.6455    0.0676    2  0


 Formation entalpies    Value   standard
                                deviation
-DeltaH A refined      42.4924   4.5528
-DeltaH B refined     -98.6733   7.2566
-DeltaH C refined    -293.3921  16.2613



++++++++++++++++++++++++++++++++++++++++++++++++++++++++++++++++++++++++++++++++

 Thermodynamic Functions, kJ/mol


             - DeltaG°               - DeltaH°                T DeltaS°
   A       19.9011    0.3866        42.4924    4.5528       -22.5913    4.4236
   B       44.0392    1.2829       -98.6733    7.2566       142.7125    8.2500
   C       15.1006    0.2656      -293.3921   16.2613       308.4927   16.2634


++++++++++++++++++++++++++++++++++++++++++++++++++++++++++++++++++++++++++++++++


 Correlation coefficients*1000         Run timed at 10.03 on 20 Oct 2020

   2  952
   3  372  634
   4 -526 -739 -951
   5  646  434 -359  283
       1    2    3    4

 Order of parameters:
   1       Beta   A
   2       Beta   B
   3      -DeltaH A
   4      -DeltaH B
   5      -DeltaH C
```

Results table

Addition (µL)	Qobs (mJ)	Qcalc (mJ)	residual (mJ)	QTobs (mJ)	QTcalc (mJ)
1.5200	-0.1268				
21.5200	-1.6732	-1.6732	0.0000	-1.8000	-1.8000
41.5200	-1.4236	-1.4227	-9.0000e-4	-3.2236	-3.2227
61.5200	-1.2864	-1.2960	9.6000e-3	-4.5100	-4.5187
81.5200	-1.1264	-1.1086	-0.0179	-5.6364	-5.6272
101.5200	-0.9578	-0.9524	-5.4000e-3	-6.5942	-6.5796
121.5200	-0.8217	-0.8286	6.9000e-3	-7.4159	-7.4082
141.5200	-0.7163	-0.7295	0.0133	-8.1321	-8.1378
161.5200	-0.6361	-0.6488	0.0127	-8.7682	-8.7866
181.5200	-0.5845	-0.5819	-2.6000e-3	-9.3527	-9.3685
201.5200	-0.5333	-0.5256	-7.7000e-3	-9.8860	-9.8941
221.5200	-0.4883	-0.4774	-0.0108	-10.3743	-10.3715
241.5200	-0.4341	-0.4359	1.8000e-3	-10.8083	-10.8074

Titration Plots

Experimental (symbols) and calculated (cross and lines) heats

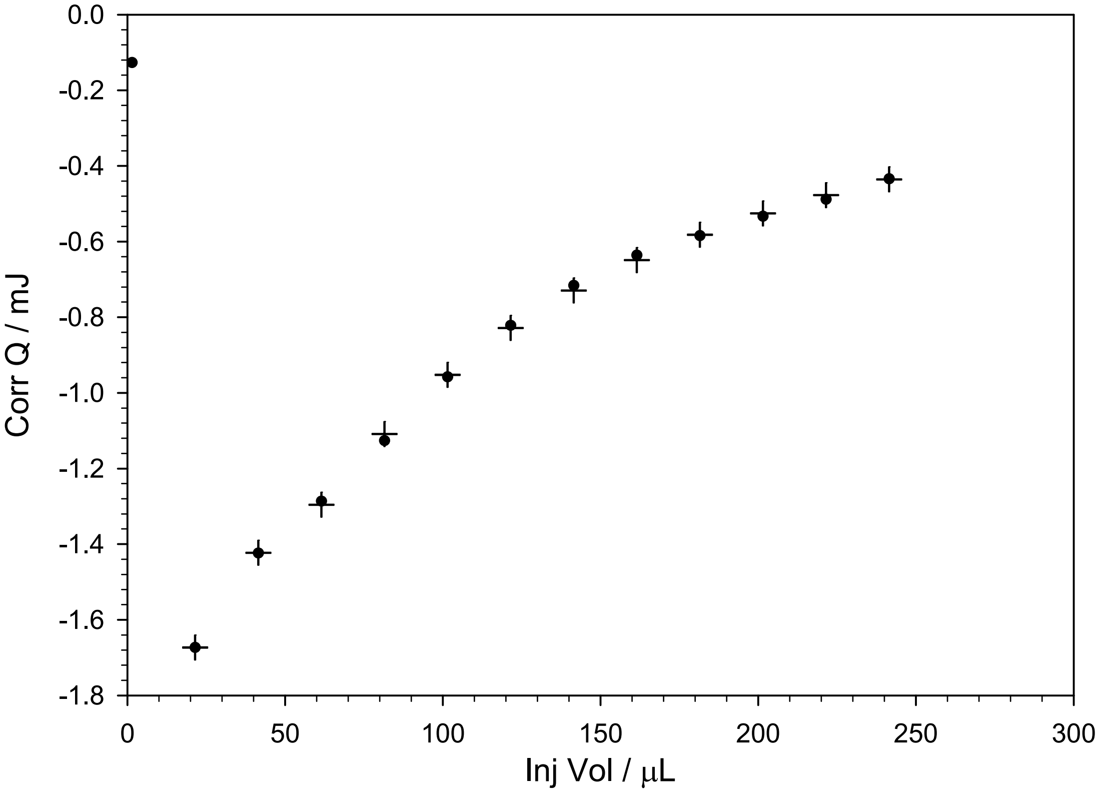

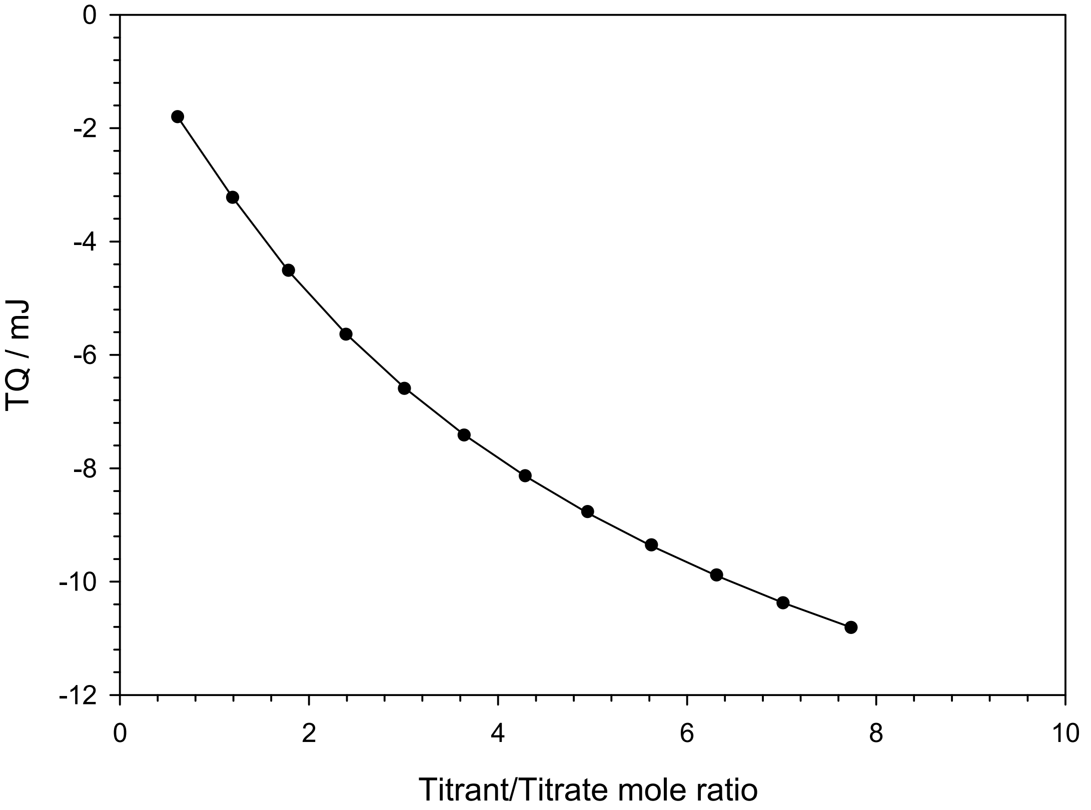

Structural studies.

NMR methods. NMR experiments were performed at 500 MHz in D_2O at pD 11 at 298 K. The experiments on the complex were performed using an equimolar solution of **1** (R) and MeβGlcNAc$_2$ (G). Because of broad signals observed for the complex, due to a medium-exchange equilibrium in the NMR time scale, related to the strong binding, a concentration of 15 mM for both **1** and MeβGlcNAc$_2$ was used to obtain an acceptable signal to noise ratio for NOESY experiments. Alkaline pD was used to obtain sharper signal of the receptor at operative 15 mM concentration. In addition to standard 1D ^{1}H NMR spectra, COSY, TOCSY, HSQC and NOESY experiments (500 ms mixing time) were also acquired to assign the resonances of all the molecular entities and to detect the relevant intramolecular and intermolecular contacts.

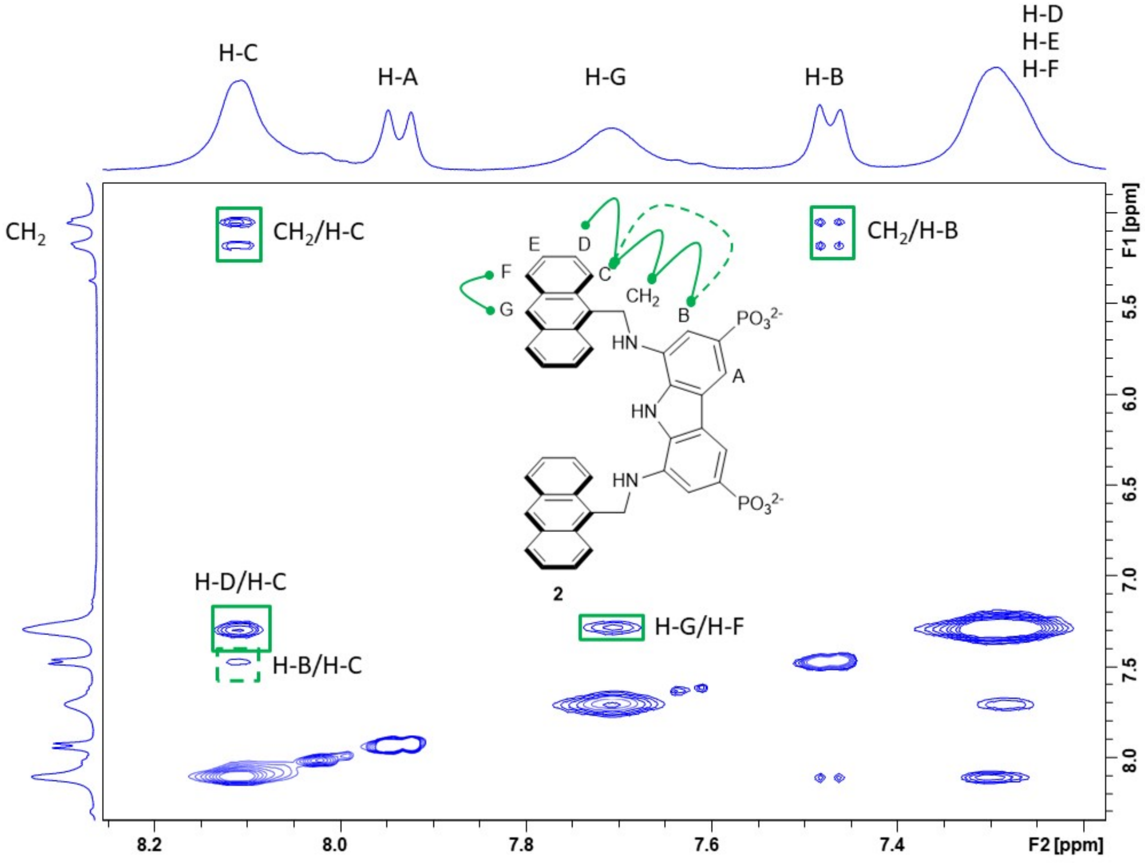

Figure S18. 500 MHz NOESY spectrum of an equimolar mixture of MeβGlcNAc$_2$ and **1** (15 mM each) in D_2O at 298 K. Intramolecular NOE cross peaks of the receptor **1** are indicated by squares and schematically represented (solid and dashed squares and lines were used to indicate strong and medium NOEs).

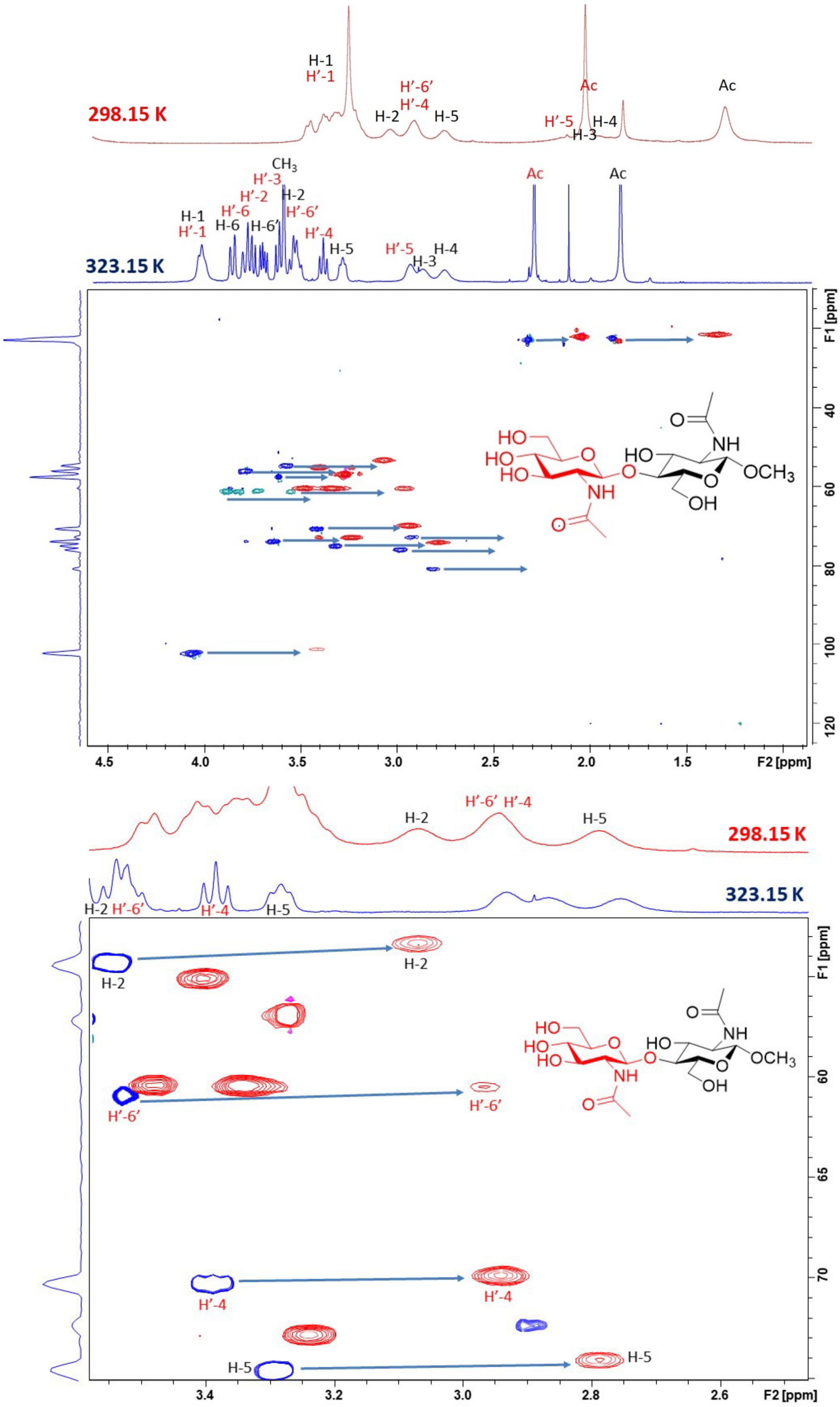

Figure S19. Superposition of 500 MHz HSQC and 1H-NMR spectra acquired at 298.15 K (in red) and 323.15 K (in blue) of an equimolar mixture of MeβGlcNAc$_2$ and **1** (15 mM each) in D$_2$O at 298 K. Temperature-dependent chemical shift variations are indicated by arrows and were used to assign proton signals of broad peaks at 298.15 K.

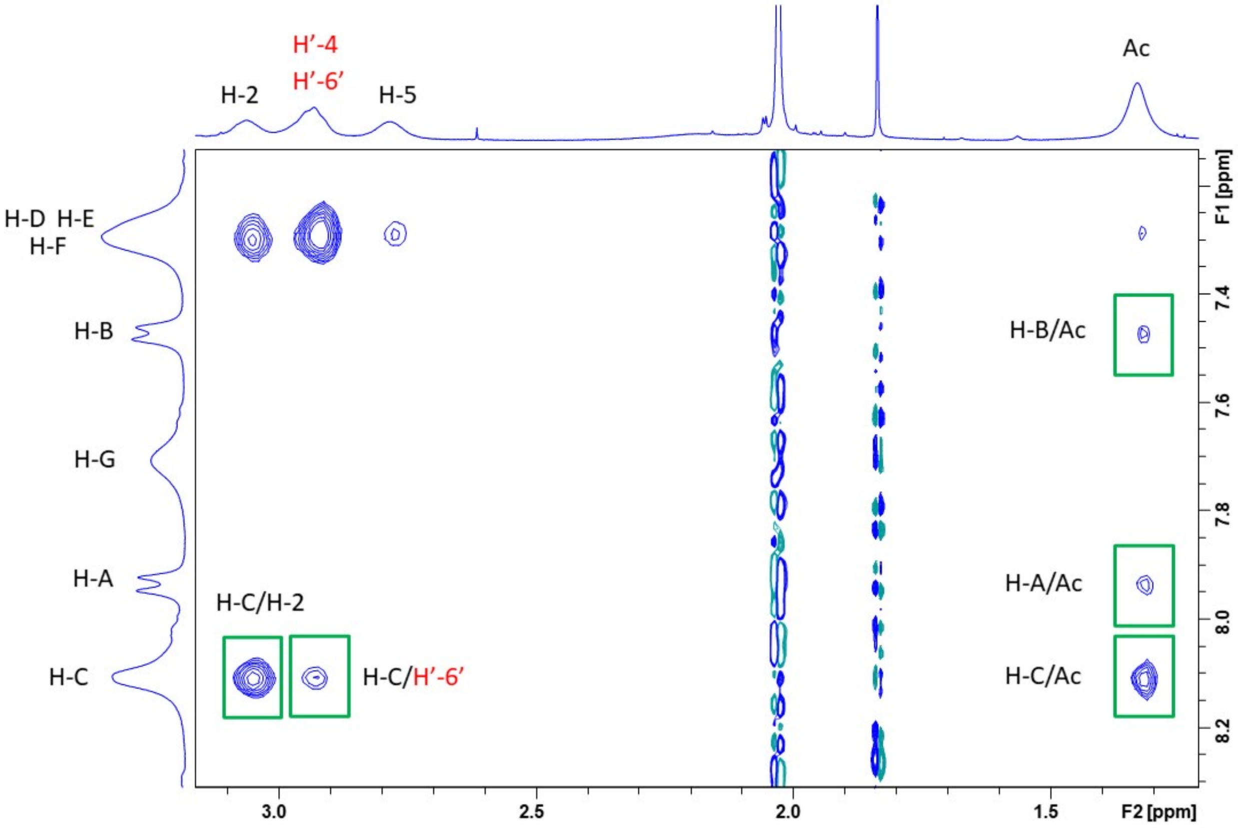

Figure S20. 500 MHz NOESY spectrum of an equimolar mixture of MeβGlcNAc$_2$ and **1** (15 mM each) in D_2O at 298 K. Unambiguous intermolecular NOE cross peaks are indicated by squares.

Molecular modeling methods. Initial structures of MeβGlcNAc$_2$ and receptor **1** were built and minimized using conjugate gradients with the OPLS_2005 force field, water was set as solvent and an extended cutoff was used to treat remote interactions. Each phosphonate group of the receptor was considered monoprotonated, accordingly with the degree of protonation observed for a structurally correlated receptor at neutral pH.[11] A maximum number of 5000 iterations were employed with the Polak-Ribiere Conjugate Gradient (PRCG) scheme, until the convergence energy threshold was 0.05. Once the optimum geometries had been achieved, a conformational search protocol was adopted for the receptors, using a Monte Carlo torsional sampling method (MCMM) with automatic setup during the calculation, energy window of 21 kJ mol^{-1}, 1000 maximum number of steps, and 100 steps per torsion of the bond to be rotated. The best structures obtained from this calculation in terms of energy were chosen and then, the disaccharide was manually docked within the receptors cleft with different starting relative orientations and further minimized. Minimization results afford different structures which were employed as input for further conformational search protocols without any constraints. Several complexes were found to be stable, in which the sugar was located inside the receptor cleft. The lowest energy structures were analyzed to check the agreement with experimental NMR data. The protocol returned a family of structures, containing the minimum energy structure of the conformational search, in agreement with the observed NOE data.

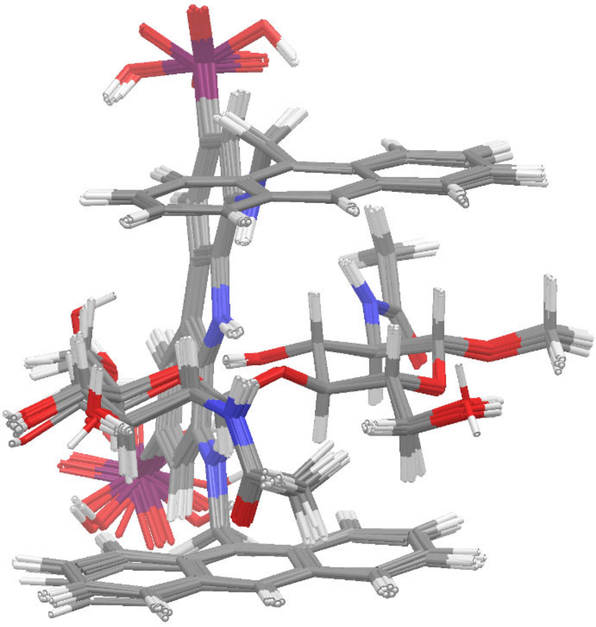

Figure S21. Molecular modelling results from conformational search for the complex of **1** with MeβGlcNAc$_2$. Superposition of the 16 energy minimum structures, within an energy window of 10.0 kJ mol^{-1}, identified among the 156 structures obtained from the calculation.

References.

S1. A. Vacca, C. Nativi, M. Cacciarini, R. Pergoli, S. Roelens, *J. Am. Chem. Soc.* **2004**, *126*, 16456-16465.

S2. C. Frassineti, S. Ghelli, P. Gans, A. Sabatini, M. S. Moruzzi, A. Vacca, *Anal. Biochem.* **1995**, *231*, 374-382.

S3. P. Gans, A. Sabatini, A. Vacca, *J. Solution Chem.* **2008**, *37*, 467-476.
